# Supplementary material for: Evidence for the effectiveness of minimum pricing of alcohol: a systematic review and assessment using the Bradford Hill criteria for causality
Source: BMJ Open. 2017 Jun 6;7(5):e013497. doi: 10.1136/bmjopen-2016-013497 (PMC5777460; doi:10.1136/bmjopen-2016-013497)
Supplement: supplementary data [file bmjopen-2016-013497supp001.pdf]

Online supplementary table: identified studies where full text assessed, then not included in review

Source	Author and year published	Study type	Reason excluded
Ovid search	Aage 2012 (1)	Time series analysis	Affordability, not price
	Ayyagari 2013 (2)	Econometric analysis of price and consumption data	Price elasticities of demand only
	Bellis 2009 (3)	Cross-sectional surveys	Association between drink type and price, and drink type and harms, but not directly reported in paper
	Bennetts 2008 (4)	Review (not systematic)	Review of a report (not a research study)
	Burton 2016 (5)	Rapid evidence review (not systematic)	Review of effectiveness and cost-effectiveness of range of policy options (including MUP), not systematic
	Callinan 2015 (6)	Editorial (not a research study)	Editorial about different pricing, taxation and afforability measures, not a research study
	Casswell 2014 (7)	Cross-sectional survey	Studies price paid for alcohol, but not in relation to a proposed minimum price (price simply dichotomised into either high or low)
	Chalmers 2013 (8)	Commentary (not a research study)	Is a commentary about challenges to MUP e.g. industry and the law
	Chaloupka 2002 (9)	Review (not systematic)	Review of previous economic studies (not systematic)
	Chick 2012 (10)	Editorial (not a research study)	Is about bans on multi-buys, not minimum price
	Cook 2014 (11)	Analysis of cross-sectional survey data and alcohol policies	Affordability not price (GDP PPP)
	Duffy 1981 (12)	Econometric time series analysis	Talks about elasticities in relation to duty rises not MUP. NB is M duffy, not J duffy
	Giesbrecht 2003 (13)	Review (not systematic)	Review of previous studies (not systematic)
	Giesbrecht 2015 (14)	Policy analysis	Does not relate price intervention to consumption or harm
	Giesbrecht 2016 (15)	Comparison of alcohol policies in different Canadian provinces	Does not relate different prices to different outcomes in terms of consumption or harm

	Gilligan 2012 (16)	Cross-sectional survey and policy analysis	Price changes, not minimum prices
	Grossman 2004 (17)	Economic analysis	Tax (federal excise tax increase) not MUP
	Hadland 2015 (18)	Policy analysis	Effects of numerous alcohol policies considered together
	Heeb 2003 (19)	Natural experiment: longitudinal survey 3 months before and 3 months after price change	Tax, not price intervention
	Hogan 2006 (20)	Natural experiment/evaluation	Tax, not price intervention
	Jónsson 2013 (21)	Cross-sectional surveys and routine data	Policies included tax and other supply side policies, but not MUP
	Katikireddi 2012 (22)	Commentary (not a research study)	Is a commentary about legal considerations of MUP, not effectiveness
	Katikireddi 2014 (23)	Qualitative study	Stakeholder views of MUP, not effectiveness
	Khaltourina 2015 (24)	Cross-sectional surveys and routine data	Tax, not price intervention
	Knibbe 2014 (25)	Analysis of cross-sectional survey data and alcohol policies	No price intervention studied, price investigated more as a covariate
	Lindeman 2013 (26)	Cross-sectional surveys and routine data	Tax, not price intervention
	Lonsdale 2012 (27)	Qualitative study	Public opinion of policy, nothing about effectiveness
	Mäkelä 2009 (28)	Review (not systematic) and analysis of routine data	Tax, not price intervention
	McCambridge 2014 (29)	Document analysis and qualitative interviews	Corporate lobbying, not the effectiveness of MUP
	Meng 2014 (30)	Econometric analysis using pseudo panel	Price elasticities of demand only
	Nelson 2013 (31)	Review (not systematic)	Conflict of interest (funded by International Center for Alcohol Policies).
	Nelson 2014 (affordability) (32)	Analysis of routine data and economic modelling	Affordability not price, and conflict of interest (funded by International Center for Alcohol Policies).
	Nelson 2014 (elasticities) (33)	Economic modelling	Conflict of interest (funded by International Center for Alcohol Policies).

	Nelson 2014 (gender) (34)	Systematic review	Conflict of interest (funded by International Center for Alcohol Policies).
	Nelson & McNall 2016 (35)	Review (not systematic)	Conflict of interest (funded by International Alliance for Responsible Drinking)
	Nicholls & Greenaway 2015 (36)	Policy analysis	Is about policy framing, not the effectiveness of MUP
	Record 2009 (37)	Modelling study	Conflict of interest – both authors were members of the Alcohol Health Alliance which is campaigning for MUP
	Rush 1986 (38)	Analysis of routine data	Affordability, not price
	Shi 2011 (39)	Econometric analysis of price and consumption data	Price elasticities of demand only
	Skov 2011 (40)	Natural experiment	Tax, not price intervention
	Sloan 1994 (41)	Analysis of routine data 1982-1988	Price changes, not minimum prices
	Stockwell 2012 (42)	Letter to journal (not a research study)	Letter concerning homelessness and price paid for alcohol
	Stockwell 2013 (43)	Letter to journal (not a research study)	Letter to editor about UK industry criticism of Canadian research on MUP, not a research study
	Sutton & Godfrey 1995 (44)	Cross-sectional survey	Price changes, not minimum prices
	Tian 2011 (45)	Econometric analysis of price and consumption data	Price elasticities of demand only
	Wall & Casswell 2013 (46)	Time series analysis and econometric modelling	Price changes, not minimum prices
	Xuan 2015 (47)	Analysis of cross-sectional survey data and alcohol policies	Price and tax considered together
	Yashkin 2013 (48)	Econometric analysis of price and consumption data	Price elasticities of demand only
Hand searching &	Ally 2014 (49)	Analysis of VAT and price data	Tax, not price intervention
	Anderson 2009 (50)	Lancet 'Series' article – a	Not an original research study.

grey literature		commentary/review	
	Byrnes 2013 (51)	Repeated cross-sectional survey	Price changes, not minimum prices
	Centre for Economics and Business Research 2010 (52)	Report (not a research study)	Conflict of interest (funded by SAB Miller). Is a critique of Sheffield modelling, not a research study.
	Craven 2013 (53)	Economic report	Published in the journal of the Institute of Economic Affairs, which has a conflict of interest.
	Duffy & Snowdon 2012 (54)	Report chapters (not a research study)	Is a critique of Sheffield modelling, not a research study.
	Fitzgerald & Angus 2015 (55)	Report (not a research study)	Is a report about use of evidence in policymaking, not about effectiveness of MUP. Also not a research study.
	Gallet 2007 (56)	Meta-analysis	Is looking at elasticities of demand for alcohol, not the effectiveness of MUP.
	Gray 2000 (57)	Natural experiment	Restrictions do not include any price interventions
	Gruenewald 2006 (58)	Time series analysis/modelling	Price changes, not minimum prices, but included in narrative
	Hilton 2014 (59)	Content analysis of UK newsprint	Media representations of MUP, not effectiveness
	Home Office 2011 (60)	'Summary review'/report (not a research study)	Replicates what we have elsewhere, but in less detail. Also not a research study.
	Institute for Fiscal Studies 2011 (61)	Report (not a research study)	Is not about effectiveness of MUP
	Institute for Fiscal Studies 2013 (62)	Report (not a research study)	Is not looking at effectiveness of MUP, is comparing Govt and industry revenue under MUP and higher tax
	Institute of Alcohol Studies (Goodliffe) 2014 (63)	Webpage	Is discussing legal issues, not effectiveness of MUP
	Kisely & Lawence 2015 (64)	Natural experiment	Tax, not price intervention
	Ludbrook 2010 (65)	Secondary analysis of Expenditure and Food Survey	Describes purchasing patterns of low price alcohol in Scotland. Not about MUP.
	Ornstein 1983 (66)	Literature review book chapter (not systematic)	Price elasticities of demand only

	Rabinovch 2012 (67)	Technical report (contains data)	Does not assess effectiveness of MUP
	Radaev 2015 (68)	Time series analysis	Paper explored the effect of price interventions on consumption of homemade alcohol, not the effectiveness of MUP
	Robinson 2013 (69)	Letter to journal (not a research study)	Letter to editor about evidence for MUP, not a research study
	Snowdon 2015 (70)	Book chapter	Is a critique of Sheffield modelling, not a research study. Institute of Economic Affairs also has a conflict of interest.
	Wine and Spirits Trade Association n.d. (71)	Press release	Press release critiquing Zhao 2013 paper, not a research study
	World Health Organisation 2014 (72)	Report	Does not discuss effectiveness of MUP

References

1. Aage H. Alcohol in Greenland 1951–2010: consumption, mortality, prices. Int J Circumpolar Health [Internet]. 2012 Dec 17 [cited 2016 Feb 27];71. Available from: http://www.ncbi.nlm.nih.gov/pmc/articles/PMC3525923/

2. Ayyagari P, Deb P, Fletcher J, Gallo W, Sindelar JL. Understanding heterogeneity in price elasticities in the demand for alcohol for older individuals. Health Econ. 2013 Jan;22(1):89–105.

3. Bellis MA, Phillips-Howard PA, Hughes K, Hughes S, Cook PA, Morleo M, et al. Teenage drinking, alcohol availability and pricing: a cross-sectional study of risk and protective factors for alcohol-related harms in school children. BMC Public Health. 2009 Oct 9;9(1):1–12.

4. Bennetts R. Russell Bennetts. Alcohol: Price, Policy and Public Health. Alcohol Alcohol. 2008 Mar 1;43(2):123–123.

5. Burton R, Henn C, Lavoie D, O'Connor R, Perkins C, Sweeney K, et al. A rapid evidence review of the effectiveness and cost-effectiveness of alcohol control policies: an English perspective. The Lancet [Internet]. 2016 Dec 2 [cited 2017 Feb 17];0(0). Available from: http://www.thelancet.com/journals/lancet/article/PIIS0140-6736(16)32420-5/abstract

6. Callinan S, Room R, Dietze P. Alcohol Price Policies as an Instrument of Health Equity: Differential Effects of Tax and Minimum Price Measures. Alcohol Alcohol Oxf Oxfs. 2015 Nov;50(6):629–30.

7. Casswell S, Huckle T, Wall M, Yeh LC. International alcohol control study: pricing data and hours of purchase predict heavier drinking. Alcohol Clin Exp Res. 2014 May;38(5):1425–31.

8. Chalmers J, Carragher N, Davoren S, O'Brien P. Real or perceived impediments to minimum pricing of alcohol in Australia: Public opinion, the industry and the law. Int J Drug Policy. 2013 Nov;24(6):517–23.

9. Chaloupka FJ, Grossman M, Saffer H. The effects of price on alcohol consumption and alcohol-related problems. Alcohol Res Health J Natl Inst Alcohol Abuse Alcohol. 2002;26(1):22–34.

10. Chick J. 16 for the Price of 10: Effects of a Ban on Multi-Buy Alcohol. Alcohol Alcohol. 2012 Feb 22;47(2):83–83.

11. Cook WK, Bond J, Greenfield TK. Are alcohol policies associated with alcohol consumption in low- and middle-income countries? Addict Abingdon Engl. 2014 Jul;109(7):1081–90.

12. Duffy M. The influence of prices, consumer incomes and advertising upon the demand for alcoholic drink in the United Kindgom: an econometric study. Alcohol Alcohol. 1981 Sep 21;16(4):200–9.

13. Giesbrecht N, Greenfield TK. Preventing Alcohol-Related Problems in the US Through Policy: Media Campaigns, Regulatory Approaches and Environmental Interventions. J Prim Prev. 2003 Sep;24(1):63–104.

14. Giesbrecht N, Wettlaufer A, Thomas G, Stockwell T, Thompson K, April N, et al. Pricing of alcohol in Canada: A comparison of provincial policies and harm-reduction opportunities. Drug Alcohol Rev. 2015 Nov 4;

15. Giesbrecht N, Wettlaufer A, Thomas G, Stockwell T, Thompson K, April N, et al. Pricing of alcohol in Canada: A comparison of provincial policies and harm-reduction opportunities. Drug Alcohol Rev. 2016 May;35(3):289–97.

16. Gilligan C, Kuntsche E, Gmel G. Adolescent drinking patterns across countries: associations with alcohol policies. Alcohol Alcohol Oxf Oxfs. 2012 Dec;47(6):732–7.

17. Grossman M. Individual Behaviors and Substance Use: The Role of Price [Internet]. National Bureau of Economic Research; 2004 Dec [cited 2016 Feb 27]. Report No.: 10948. Available from: http://www.nber.org/papers/w10948

18. Hadland SE, Xuan Z, Blanchette JG, Heeren TC, Swahn MH, Naimi TS. Alcohol Policies and Alcoholic Cirrhosis Mortality in the United States. Prev Chronic Dis [Internet]. 2015 Oct 15 [cited 2016 Feb 27];12. Available from: http://www.cdc.gov/pcd/issues/2015/15_0200.htm

19. Heeb J-L, Gmel G, Zurbrügg C, Kuo M, Rehm J. Changes in alcohol consumption following a reduction in the price of spirits: a natural experiment in Switzerland. Addict Abingdon Engl. 2003 Oct;98(10):1433–46.

20. Hogan E, BOFFA J, ROSEWARNE C, BELL S, CHEE DA. What price do we pay to prevent alcohol-related harms in Aboriginal communities? The Alice Springs trial of liquor licensing restrictions. Drug Alcohol Rev. 2006;25(3):207–212.

21. Jónsson RM, Kristjánsson S. Alcohol policy and public opinion in Iceland, 1989–2012. Nord Stud Alcohol Drugs. 2013;30(6):539–549.

22. Katikireddi SV, McLean JA. Introducing a minimum unit price for alcohol in Scotland: considerations under European Law and the implications for European public health. Eur J Public Health. 2012 Aug 1;22(4):457–8.

23. Katikireddi SV, Bond L, Hilton S. Perspectives on econometric modelling to inform policy: a UK qualitative case study of minimum unit pricing of alcohol. Eur J Public Health. 2014 Jun 1;24(3):490–5.

24. Khaltourina D, Korotayev A. Effects of Specific Alcohol Control Policy Measures on Alcohol-Related Mortality in Russia from 1998 to 2013. Alcohol Alcohol. 2015 Sep 1;50(5):588–601.

25. Knibbe RA, Derickx M, Allamani A, Massini G. Alcohol Consumption and its Related Harms in the Netherlands Since 1960: Relationships With Planned and Unplanned Factors. Subst Use Misuse. 2014 Oct 15;49(12):1589–600.

26. Lindeman M, Karlsson T, Österberg E. Public opinions, alcohol consumption and policy changes in Finland, 1993–2013. Nord Stud Alcohol Drugs. 2013;30(6):507–524.

27. Lonsdale AJ, Hardcastle SJ, Hagger MS. A minimum price per unit of alcohol: A focus group study to investigate public opinion concerning UK government proposals to introduce new price controls to curb alcohol consumption. BMC Public Health. 2012 Nov 23;12(1):1023.

28. Mäkelä P, Osterberg E. Weakening of one more alcohol control pillar: a review of the effects of the alcohol tax cuts in Finland in 2004. Addict Abingdon Engl. 2009 Apr;104(4):554–63.

29. McCambridge J, Hawkins B, Holden C. Vested Interests in Addiction Research and Policy. The challenge corporate lobbying poses to reducing society's alcohol problems: insights from UK evidence on minimum unit pricing. Addiction. 2014 Feb 1;109(2):199–205.

30. Meng Y, Brennan A, Purshouse R, Hill-McManus D, Angus C, Holmes J, et al. Estimation of own and cross price elasticities of alcohol demand in the UK--A pseudo-panel approach using the Living Costs and Food Survey 2001-2009. J Health Econ. 2014 Mar;34:96–103.

31. Nelson JP. Does Heavy Drinking by Adults Respond to Higher Alcohol Prices and Taxes? A Survey and Assessment. Econ Anal Policy. 2013 Dec;43(3):265–91.

32. Nelson JP. Alcohol Affordability and Alcohol Demand: Cross-Country Trends and Panel Data Estimates, 1975 to 2008. Alcohol Clin Exp Res. 2014 Apr 1;38(4):1167–75.

33. Nelson JP. Estimating the Price Elasticity of Beer: Meta-Analysis of Data with Heterogeneity, Dependence, and Publication Bias [Internet]. Rochester, NY: Social Science Research Network; 2013 Jan [cited 2016 Feb 27]. Report No.: ID 2200492. Available from: http://papers.ssrn.com/abstract=2200492

34. Nelson JP. Gender differences in alcohol demand: a systematic review of the role of prices and taxes. Health Econ. 2014 Oct;23(10):1260–80.

35. Nelson JP, McNall AD. What happens to drinking when alcohol policy changes? A review of five natural experiments for alcohol taxes, prices, and availability. Eur J Health Econ. 2016 Apr 7;1–18.

36. Nicholls J, Greenaway J. What is the problem?: Evidence, politics and alcohol policy in England and Wales, 2010–2014. Drugs Educ Prev Policy. 2015 Mar 4;22(2):135–42.

37. Record C, Day C. Britain's alcohol market: how minimum alcohol prices could stop moderate drinkers subsidising those drinking at hazardous and harmful levels. Clin Med. 2009 Oct 1;9(5):421–5.

38. Rush B, Steinberg M, Brook R. The relationships among alcohol availability, alcohol consumption and alcohol-related damage in the Province of Ontario and the State of Michigan 1955-1982. Adv Alcohol Subst Abuse. 1986;5(4):33–45.

39. Shi Y. Three Essays on Economics of Health Behavior in China [Internet]. 2011 [cited 2016 Mar 20]. Available from: http://www.rand.org/pubs/rgs_dissertations/RGSD287.html

40. Skov SJ, Chikritzhs TN, Kypri K, Miller PG, Hall WD, Daube MM, et al. Is the "alcopops" tax working? Probably yes but there is a bigger picture. Med J Aust [Internet]. 2011 [cited 2015 Nov

9];195(2). Available from: https://www.mja.com.au/journal/2011/195/2/alcopops-tax-working-probably-yes-there-bigger-picture

41. Sloan FA, Reilly BA, Schenzler C. Effects of prices, civil and criminal sanctions, and law enforcement on alcohol-related mortality. J Stud Alcohol. 1994 Jul;55(4):454–65.

42. Stockwell T, Williams N, Pauly B. Working and waiting: Homeless drinkers responses to less affordable alcohol. Drug Alcohol Rev. 2012 Sep 1;31(6):823–4.

43. Stockwell T, Zhao J, Martin G, Macdonald S, Vallance K, Treno A, et al. Misleading UK alcohol industry criticism of Canadian research on minimum pricing. Addiction. 2013 Jun 1;108(6):1172–3.

44. Sutton M, Godfrey C. A grouped data regression approach to estimating economic and social influences on individual drinking behaviour. Health Econ. 1995 May 1;4(3):237–47.

45. Tian G, Liu F. Is the demand for alcoholic beverages in developing countries sensitive to price? Evidence from China. Int J Environ Res Public Health. 2011 Jun;8(6):2124–31.

46. Wall M, Casswell S. Affordability of alcohol as a key driver of alcohol demand in New Zealand: a co-integration analysis. Addict Abingdon Engl. 2013 Jan;108(1):72–9.

47. Xuan Z, Blanchette J, Nelson TF, Heeren T, Oussayef N, Naimi TS. The alcohol policy environment and policy subgroups as predictors of binge drinking measures among US adults. Am J Public Health. 2015 Apr;105(4):816–22.

48. Yashkin A. The Dynamics of Alcohol Consumption in the Russian Federation: Implications of Using Price Related Policies to Control Alcohol Use. Grad Theses Diss [Internet]. 2013 Jan 1; Available from: http://scholarcommons.usf.edu/etd/4968

49. Ally AK, Meng Y, Chakraborty R, Dobson PW, Seaton JS, Holmes J, et al. Alcohol tax pass-through across the product and price range: do retailers treat cheap alcohol differently? Addiction. 2014 Dec 1;109(12):1994–2002.

50. Anderson P, Chisholm D, Fuhr DC. Effectiveness and cost-effectiveness of policies and programmes to reduce the harm caused by alcohol. Lancet Lond Engl. 2009 Jun 27;373(9682):2234–46.

51. Byrnes J, Shakeshaft A, Petrie D, Doran C. Can harms associated with high-intensity drinking be reduced by increasing the price of alcohol? Drug Alcohol Rev. 2013 Jan;32(1):27–30.

52. Centre for Economics and Business Research. Minimum Alcohol Pricing: A targeted measure? Report to the Scottish Parliamentary Health and Sport Committee [Internet]. London; 2010 Aug. Available from: http://www.ias.org.uk/uploads/pdf/Price%20docs/Updated-Sheffield-Scotland-v2-August-20103.pdf

53. Craven BM, Marlow ML, Shiers AF. The Economics of Minimum Pricing for Alcohol. Econ Aff. 2013 Jun 1;33(2):174–89.

54. Duffy J, Snowdon C. The minimal evidence for minimum pricing [Internet]. Adam Smith Institute; Available from: http://www.adamsmith.org/sites/default/files/research/files/ASI_SAPM.pdf

55. Fitzgerald N, Angus C. Four Nations: How Evidence-based are Alcohol Policies and Programmes across the UK?. London: [Internet]. London: Alliance for Useful Evidence/Alcohol Health Alliance.; 2015. Available from: http://www.alliance4usefulevidence.org/assets/Four-Nations-v3.pdf

56. Gallet CA. The Demand for Alcohol: A Meta-Analysis of Elasticities [Internet]. Rochester, NY: Social Science Research Network; 2007 May [cited 2016 Jan 6]. Report No.: ID 985689. Available from: http://papers.ssrn.com/abstract=985689

57. Gray D, Saggers S, Atkinson D, Sputore B, Bourbon D. Beating the grog: an evaluation of the Tennant Creek liquor licensing restrictions. Aust N Z J Public Health. 2000 Feb;24(1):39–44.

58. Gruenewald PJ, Ponicki WR, Holder HD, Romelsjö A. Alcohol prices, beverage quality, and the demand for alcohol: quality substitutions and price elasticities. Alcohol Clin Exp Res. 2006 Jan;30(1):96–105.

59. Hilton S, Wood K, Patterson C, Katikireddi SV. Implications for alcohol minimum unit pricing advocacy: What can we learn for public health from UK newsprint coverage of key claim-makers in the policy debate? Soc Sci Med. 2014 Feb;102:157–64.

60. Home Office. The likely impacts of increasing alcohol price: a summary review of the evidence base [Internet]. 2011 Jan. Available from: https://www.gov.uk/government/uploads/system/uploads/attachment_data/file/98100/impacts-alcohol-price-review.pdf

61. Institute for Fiscal Studies. Alcohol pricing and taxation policies. IFS Briefing Note BN124. 2011.

62. Institute for Fiscal Studies. Price-based measures to reduce alcohol consumption. IFS Briefing Note BN138. 2013.

63. Goodliffe J. Applying a minimum price to alcohol (Institute of Alcohol Studies) [Internet]. 2014. Available from: http://www.ias.org.uk/What-we-do/Publication-archive/Alcohol-Alert/October-2014/Applying-a-minimum-price-to-alcohol.aspx

64. Kisely S, Lawrence D. A time series analysis of alcohol-related presentations to emergency departments in Queensland following the increase in alcopops tax. J Epidemiol Community Health. 2015 Sep 16;jech-2015-205666.

65. Ludbrook A. Purchasing Patterns for Low Price Off Sales Alcohol: Evidence from the Expenditure and Food Survey [Internet]. Available from: http://www.shaap.org.uk/images/UserFiles/File/Reports%20and%20Briefings/Briefing%20-%20Purchase%20of%20low-price%20alcohol%20analysis.pdf

66. Ornstein SI, Levy D. Price and Income Elasticities of Demand for Alcoholic Beverages. In: Galanter M, Begleiter H, Cicero T, Deitrich R, Goodwin DW, Gottheil E, et al., editors. Genetics

Behavioral Treatment Social Mediators and Prevention Current Concepts in Diagnosis [Internet]. Boston, MA: Springer US; 1983. p. 303–45. Available from: http://dx.doi.org/10.1007/978-1-4613-3617-4_18

67. Rabinovich L, Brutscher P-B, de Vries H, Tiessen J, Clift J, Reding A. The affordability of alcoholic beverages in the European Union. 2012;

68. Radaev V. Impact of a New Alcohol Policy on Homemade Alcohol Consumption and Sales in Russia. Alcohol Alcohol. 2015 May 1;50(3):365–72.

69. Robinson M, McCartney G, Beeston C. What is convincing evidence on alcohol pricing? BMJ. 2013 Aug 20;347:f5102.

70. Snowdon C. Chapter 10. Minimum unit pricing. In: Flaws and Ceilings: Price controls and the damage they cause [Internet]. [cited 2015 Nov 9]. p. 177–97. Available from: http://www.iea.org.uk/publications/research/flaws-and-ceilings-price-controls-and-the-damage-they-cause

71. Wine and Spirits Trade Association. New claims that increasing the cost of alcohol saves lives are misleading [Internet]. Available from: http://www.wsta.co.uk/press/634-new-claims-that-increasing-the-cost-of-alcohol-saves-lives-are-misleading

72. World Health Organization. European status report on alcohol and health 2014. Pricing policies [Internet]. World Health Organization; 2014. Available from: http://www.euro.who.int/__data/assets/pdf_file/0003/244902/Pricing-policies.pdf
