# Supplementary material for: Evidence for the effectiveness of minimum pricing of alcohol: a systematic review and assessment using the Bradford Hill criteria for causality
Source: BMJ Open. 2017 Jun 6;7(5):e013497. doi: 10.1136/bmjopen-2016-013497 (PMC5777460; doi:10.1136/bmjopen-2016-013497)
Supplement: Author's manuscript [file bmjopen-2016-013497.draft_revisions.pdf]

Evidence for the effectiveness of minimum pricing of alcohol: a systematic review and assessment using the Bradford Hill criteria for causality

Journal:	*BMJ Open*
Manuscript ID	bmjopen-2016-013497
Article Type:	Research
Date Submitted by the Author:	15-Jul-2016
Complete List of Authors:	Boniface, Sadie; King's College London, National Addiction Centre, Institute of Psychiatry, Psychology and Neuroscience<br>Scannell, Jack; University of Edinburgh, Innogen Institute, School of Social and Political Sciences<br>Marlow, Sally; King's College London, National Addiction Centre, Institute of Psychiatry, Psychology and Neuroscience
**Primary Subject Heading**:	Public health
Secondary Subject Heading:	Addiction
Keywords:	alcohol, policy, minimum unit pricing, PUBLIC HEALTH, Bradford Hill

SCHOLARONE™
Manuscripts

For peer review only

Evidence for the effectiveness of minimum pricing of alcohol: a systematic review and assessment using the Bradford Hill criteria for causality

Sadie Boniface,[1] Jack W Scannell,[2] Sally Marlow[1]

[1]National Addiction Centre, Institute of Psychiatry, Psychology and Neuroscience, King's College London, London, UK

[2]School of Social and Political Science, University of Edinburgh, Edinburgh, UK

Corresponding author: Dr Sadie Boniface, National Addiction Centre, Addiction Sciences Building (PO 48) Institute of Psychiatry, Psychology and Neuroscience, 16 De Crespigny Park, King's College London, SE5 8AF. sadie.boniface@kcl.ac.uk 020 7848 5097

Keywords: alcohol, policy, minimum unit pricing, public health, Bradford Hill

Word count: 3,128 (excluding title page, abstract, references, figures and tables)

For peer review only

ABSTRACT

Objectives: To assess the evidence for price-based alcohol interventions to determine whether minimum unit pricing policies are likely to be effective.

Design: Systematic review and assessment of studies according to PRISMA guidelines, against the Bradford Hill criteria for causality. Three electronic databases were searched from inception to February 2016. Additional articles were found through hand searching and grey literature searches.

Criteria for selecting studies: We included any study design that reported on the effect of price changes and price-based interventions on alcohol consumption or alcohol-related morbidity, mortality, and wider harms. Studies reporting on the effects of taxation or affordability, and studies that only investigated price elasticity of demand were beyond the scope of this review. Studies with any conflict of interest were excluded. All studies were appraised for methodological quality.

Results: Of 756 studies assessed, 35 studies were included: 28 peer-reviewed research studies and 7 from the grey literature. All nine of the Bradford Hill criteria were met, although different types of study satisfied different criteria. For example, modelling studies complied with the consistency and specificity criteria, time series analyses demonstrated the temporality and experiment criteria, and the analogy criterion was fulfilled by comparing the findings with the wider literature on taxation and affordability.

Conclusions: Overall, the Bradford-Hill criteria for causality were satisfied. There was very little evidence that alcohol price was not associated with consumption or subsequent harms. However the overall quality of the evidence was variable, a large proportion of the evidence base has been produced by a small number of research teams, and the quantitative uncertainty in many estimates or forecasts is poorly communicated in the literature. None the less, price-based alcohol policy interventions such as minimum unit pricing are likely to reduce alcohol consumption, alcohol-related morbidity and mortality.

ARTICLE SUMMARY

Strengths and limitations of this study

- This review adds to an emerging literature of systematic reviews synthesising findings using the Bradford Hill criteria for causality in research areas where traditional meta-analyses of randomised controlled trials are not possible or appropriate
- A range of study designs were included, allowing for a comprehensive review of a disparate evidence base to investigate whether minimum unit pricing of alcohol is likely to reduce alcohol consumption and alcohol-related harm
- Studies examining the effects of alcohol taxation or changes in alcohol affordability, or studies solely reporting on price elasticity of demand, were not included
- Methodological quality of studies was variable

For peer review only

INTRODUCTION

Alcohol-related harm costs the NHS £3.5bn each year and the estimated cost to society is £21 billion per year (1). The latest annual figures for England show over one million alcohol-related hospital admissions (2013/14) and six and a half thousand alcohol-related deaths (2013); and these figures represent increases compared with a decade previously of 115% and 10% respectively (1). There are many policies and programmes that aim to reduce harms from alcohol (2), and one of these is minimum pricing. Minimum pricing for alcohol has been introduced in a number of countries around the world including Canada (3), Belarus, Kyrgyzstan, the Republic of Moldova, the Russian Federation and Ukraine (4). In 2012 the UK coalition Government cited support for minimum unit pricing (MUP) in its alcohol strategy (5), and legislation to have a minimum price of £0.50 per unit (one UK unit = 10ml or 8g ethanol) was passed in Scotland the same year (4). In England and Wales there has been a ban on alcohol being sold at below cost (the total amount of 'duty plus VAT') since May 2014 (6); and the first conviction for selling alcohol below this level recently took place (7). Duty plus VAT is equivalent to a 70cl bottle of vodka (37.5% ABV) costing a minimum of £8.72 (8), whereas under a minimum price of 50 pence per unit this would cost £13.13.

Subsequent to the publication of the UK Alcohol Strategy, the Government has withdrawn its support for MUP. There have also been discussions in the Scottish courts between health organisations and the alcohol industry around the legality of MUP, proportionality (that the same objective cannot be met through increased taxation), and whether there is sufficient evidence. In a recent report about the extent to which UK alcohol policies are evidence-based, Fitzgerald and Angus wrote that "there are also a number of notable instances of policies being rejected due to 'insufficient evidence' with little indication of what level of evidence would be considered to be 'sufficient'" (9).

Taxation and price interventions are sometimes considered analogous, however it is at the retailers' discretion whether or not to pass on tax increases to consumers, but this is not the case for MUP. In this paper, we assess the effect of price and price-based interventions as MUP is currently being considered as a policy option in the United Kingdom. We systematically review the literature on the effect of price changes or policies such as MUP on alcohol consumption, alcohol-related morbidity and mortality, and wider harms. We use the nine Bradford Hill criteria for causality as a framework with the aim of assessing the likely effectiveness of MUP as a policy to reduce alcohol consumption and alcohol-related harm.

METHODS

A systematic literature search was performed according to Preferred Reporting Items for Systematic Reviews and Meta-Analyses (PRISMA) guidance (see Figure 1 for PRISMA flow diagram and online supplementary file for excluded studies).

Identification of studies

Three electronic databases were searched for titles or abstracts containing '"minimum unit pric$" OR "minimum pric$"' OR "floor pric$" OR "pric$ AND policy" AND alcohol. The databases were PsycINFO (1806 to February Week 1 2016), Embase (1974 to 2016 Week 07), Ovid Medline (1946 to

For peer review only

February Week 1 2016). We also searched the websites of five alcohol charities for publications or reports related to "price", and also searched 20 leading UK think tanks for "alcohol" or "addiction".

Inclusion criteria were: any study design; population level studies exploring at least one aspect of the effect of changes in the price of alcohol, including but not limited to changes in alcohol sales, consumption, morbidity and mortality; individual level studies exploring differences in price paid for alcohol, and alcohol purchasing, consumption, morbidity and mortality; written in English.

Exclusion criteria were: studies about taxation, affordability and price elasticity of demand for alcohol(there is a large literature on each of these already and reviewing all of these studies was beyond the scope of this review); studies about public perceptions of MUP; studies where a conflict of interest was evident, whether in favour of or against MUP.

All 37 studies that met the inclusion criteria were assessed against the Bradford Hill criteria for causality and the methodological quality appraised. These included 28 original research studies, 2 systematic reviews, and 7 studies from the grey literature. Of the 28 research studies, there were 12 cross sectional surveys, 10 time series analyses or similar, 4 econometric modelling studies, one qualitative study, and one trial.

Analysis of included studies

Quality of included studies was assessed independently by two reviewers and using validated tools. Due to the wide variation in study designs among the included studies, the Effective Public Health Practice Project's (EPHPP) tool was used for assessing all quantitative studies, as recommended by the Cochrane Handbook for assessing studies in public health (10). Qualitative studies (n=1) and systematic reviews (n=2) included in this review were not covered by the EPHPP tool and so were assessed using the Critical Appraisal Skills Programme (CASP) tools specific to these study designs.

Nine criteria in order to determine causality were suggested by Bradford Hill in an influential 1965 paper (11). Increasingly, the Bradford Hill criteria are a standard framework to assess the impact of interventions where it is not ethical or practical to conduct randomised controlled trials. Our interpretation of the Bradford Hill criteria for the purpose of this review is listed in Table 1. Two reviewers assessed each study against each of the nine criteria and agreed which studies provided relevant evidence for or against each criterion.

For peer review only

Table 1: Bradford Hill criteria for assessing causation and the definitions used in this review

Criterion	Bradford Hill criteria (1965)	Application in this review
1. Strength of the association	The strength of a supposed association between an intervention and an outcome is determined by the appropriate statistic used to measure the protective effect of an intervention (e.g. relative risk or odds ratio). This is the most important factor determining causation	A statistically significant change (P<0.05) in alcohol consumption or alcohol related harms, in the expected direction. The exact magnitude of the association was assessed on a study by study basis
2. Consistency	Has it been repeatedly observed by different persons, in different places, circumstances and times?	Whether different studies conducted in different locations, in different populations, by different investigators and at different times have reported similar findings
3. Specificity	Specificity is present when the intervention is exclusive to the outcome and when the outcome has no other known cause or associated risk factors; cautions that this criterion should not be overemphasized and that if specificity is not apparent this does not preclude causation	If pricing was the only reason that alcohol consumption or alcohol-related harm could have fallen, this adds to the argument for causality. However if a price intervention was one of a number of alcohol policy interventions, then this criterion is not satisfied
4. Temporality	Refers to temporal relationship of association between exposure and disease outcome; to infer causality, exposure must precede outcome	The pricing intervention studied must have taken place before a change in alcohol consumption or harm was observed
5. Dose-response/biological gradient	If the association is one in which a dose-response curve or biological gradient can be observed, this adds to the case for causality	If interventions leading to a larger increase in prices had a greater effect on alcohol consumption and alcohol related harm than interventions where the price change was small, or if studies demonstrate that different prices have differing effects, in the expected direction
6. Plausibility (biological)	A likely biological mechanism linking the intervention to the observed findings helps to explain causality, plausibility depends on biological knowledge of the day	It is well-established that alcohol consumption causes health and social harm. Studies that found an association between price and alcohol-related harms could demonstrate plausibility
7. Coherence	When the evidence from different disciplines sources "hangs well together" and does not conflict with other generally known facts, this criterion is met	Describes whether studies conducted in different settings or disciplines had complementary findings. Will not be demonstrated by a single study in isolation but rather the evidence base as a whole
8. Experiment	Experimental evidence from laboratory studies or RCTs could potentially provide strongest support for causation	In addition to laboratory studies and RCTs, natural experiments with before-and-after measures could also show the

For peer review only

	This criterion often provides the strongest support for causation and describes whether there is empirical evidence for the association	effectiveness of minimum unit pricing in a 'real world' setting
9. Analogy	Causality is supported by analogy if there are similar associations or causal relationships in other areas of relevance, weakest form of evidence of causality	Other areas of relevance include whether higher taxation on alcohol is associated with reduced alcohol consumption and alcohol related harm, and may require drawing on additional literature outside of the main systematic review

For peer review only

RESULTS

The included studies that are published in peer-reviewed journals (28 research studies and two systematic reviews) are listed in Table 2 with study characteristics and methodological quality. Of the research studies, the methodological quality was rated as 'strong' in 12 studies, 'moderate' in 12 studies, and 'weak' in 4 studies. Both of the systematic reviews were rated 'strong'. The seven reports from the grey literature are listed in Table 3. Five of the seven were rated as of 'strong' methodological quality, with the remaining two not appropriate to rate using our critical appraisal tool.

For peer review only

Table 2: Studies published in peer-reviewed journals included in Bradford Hill criteria assessment

	First author and year published	Study characteristics					Study assessment			Bradford Hill criteria met*
		Country	Study design	Population or participants	Pricing intervention studied	Outcomes studied	Peer-reviewe d	Conflict of interest	Quality rating	
Original research studies	Babor 1978 (12)	USA	Trial (not randomised)	34 male volunteers in live-in research facility	'Happy Hour' with a reduction in price of alcohol for one group of participants	Alcohol consumption	Yes	Not stated	Weak	SA, CON, SP, TE, CO, EX
	Bhattachary a 2013 (13)	Russia	Time series analysis of panel data set	Populations of 77 Russian oblasts (provinces), 1970-2000	Substantial increases in alcohol prices 1985-1988, along with 6 other anti-alcohol measures	Mortality	Yes	Not stated	Strong	SA, CON, TE, PL, CO, EX,
	Black 2011(14)	Scotland	Cross-sectional survey	377 hospital patients with serious alcohol problems	Mean price paid per unit	Alcohol consumption	Yes	None	Moderat e	SA CON, DR, CO,
	Brennan 2014 (15)	England	Modelling study using SAPM	UK national surveys of general population (subgroups of moderate, harmful, hazardous)	MUP of £0.40, £0.45 and £0.50. Ban on below cost selling	Alcohol consumption, consumer spending, 47 health harms, QALYs	Yes	None	Strong	CON, SP, DR, PL, CO
	Byrnes 2013	Australia	Repeated	79,545 adults	Modelled 1%	Alcohol	Yes	Not	Moderat	SA (low

For peer review only - http://bmjopen.bmj.com/site/about/guidelines.xhtml

First author and year published	Study characteristics					Study assessment			Bradford Hill criteria met*
	Country	Study design	Population or participants	Pricing intervention studied	Outcomes studied	Peer-reviewe d	Conflict of interest	Quality rating	
(16)		cross-sectional survey		increase in the price of alcohol	consumption (with a focus on high intensity drinking)		stated	e	drinking intensity only), CON (counter)
Callinan 2015 (17)	Australia	Cross-sectional survey	Drinkers 18+ participating in Australian International Alcohol Control study (n=1,681)	Price paid for alcohol	Alcohol consumption	Yes	Not stated	Moderat e	SA, CON, DR, CO
Casswell 2014 (18)	New Zealand	Cross-sectional survey	Drinkers 18+ participating in NZ International Alcohol Control study (n=1,900)	Price paid per drink in on and off trade	Alcohol consumption	Yes	Not stated	Strong	SA, CON, CO
Crawford 2012 (19)	England	Cross-sectional survey	515 members of the public	Median price paid per unit	AUDIT score	Yes	None	Moderat e	SA, CON, CO
Falkner 2015 (20)	New Zealand	Cross-sectional survey	115 adults undergoing alcohol detoxification	Price paid for alcohol	Alcohol consumption	Yes	No	Moderat e	SA, CON, CO
Forsyth 2014 (21)	Scotland	Cross-sectional survey	Shopkeepers of 144 off licences in Glasgow	MUP of £0.50	Products affected, and hospital admissions	Yes	None	Weak	CON, PL (weakly), CO
Gilligan 2012 (22)	40 European	Cross-sectional	Adolescents aged 15-16 from	Alcohol price as a % of EU	Weekly drinking and	Yes	Not stated	Weak	SA, CON, DR, CO

For peer review only

For peer review only - http://bmjopen.bmj.com/site/about/guidelines.xhtml

	First author and year published	Study characteristics					Study assessment			Bradford Hill criteria met*
		Country	Study design	Population or participants	Pricing intervention studied	Outcomes studied	Peer-reviewe d	Conflict of interest	Quality rating	
		and North American countries	survey and policy analysis	two large cross-country surveys (HBSC & ESPAD)	average	drunkenness				(some counter findings)
	Herttua 2015 (23)	Finland	Time series analysis	General population using population registry	Modelled 1% increase in the average minimum price of all alcoholic beverages based on actual price increases	Alcohol related mortality	Yes	None	Strong	SA (not universal findings – subgroup only), CON (counter) , TE, PL, CO, EX
	Holmes 2014 (24)	England	Modelling study using SAPM	UK national surveys of general population (subgroups of moderate, harmful, hazardous)	MUP of 45p	Alcohol consumption, consumer spending, 47 health harms, QALYs	Yes	None	Strong	CON, SP, PL, CO
	Ludbrook 2012 (25)	UK	Cross-sectional survey	Expenditure and Food Survey data from 2006-8 (n=18,624)	Purchasers of alcohol less than £0.45 per unit	Income of purchasers of cheap alcohol	Yes	Not stated	Moderat e	SA, CON, CO
	Meier 2009 (26)	UK	Modelling study using SAPM	UK national surveys of general population (subgroups of	10 pricing policy options, including different levels of MUP (of 33	Alcohol consumption, consumer spending, 47 health harms,	Yes	None	Strong	CON, SP, DR, PL, CO

For peer review only

For peer review only - http://bmjopen.bmj.com/site/about/guidelines.xhtml

	First author and year published	Study characteristics					Study assessment			Bradford Hill criteria met*
		Country	Study design	Population or participants	Pricing intervention studied	Outcomes studied	Peer-reviewed	Conflict of interest	Quality rating	
				moderate, harmful, hazardous)	analysed)	crime, employment				
	Purshouse 2010 (27)	England	Modelling study using SAPM	UK national surveys of general population (subgroups of moderate, harmful, hazardous)	18 different pricing policies	Alcohol consumption, consumer spending, 47 health harms, QALYs	Yes	None	Strong	CON, SP, DR, PL, CO
	Seaman 2013 (28)	Scotland	Qualitative study	130 participants aged 16-30	Hypothetical price increases	Alcohol consumption and substitution with other substances	Yes	None	Moderate	CON, CO
	Sharma 2014 (29)	Australia	Cross-sectional survey	Representative sample of households (n=885) completing shopping survey	MUP of A$1, and taxation	Alcohol consumption (measured by projected sales)	Yes	None	Moderate	SA, CON, DR, CO
	Sheron 2014 (30)	UK	Cross-sectional survey	Adult patients in a liver unit of a hospital (n=204)	Median and mean price paid per unit	Alcohol consumption	Yes	Not stated	Moderate	SA, CON, DR, CO
	Sloan 1994 (31)	USA	Analysis of routine data 1982-1988	Population of USA	Real price (based on off-trade price data)	Alcohol related mortality	Yes	Not stated	Moderate	SA, CON, CO (partially)

For peer review only

For peer review only - http://bmjopen.bmj.com/site/about/guidelines.xhtml

	First author and year published	Study characteristics					Study assessment			Bradford Hill criteria met*
		Country	Study design	Population or participants	Pricing intervention studied	Outcomes studied	Peer-reviewed	Conflict of interest	Quality rating	
	Stockwell 2012 (3)	Canada	Cross-section versus time series analysis of ecological data	Population of British Columbia	Actual minimum price increased over a 20 year period. Study modelled a 10% increase in the average minimum price of all alcoholic beverages	Alcohol consumption (measured by sales)	Yes	None	Strong	SA, CON, TE, DR, CO, EX
	Stockwell 2012 (32)	Canada	Cross-section versus time series analysis of ecological data	Population of Saskatchewan	Actual minimum price increased over a 7 year period. Study modelled a 10% increase in the average minimum price of all alcoholic beverages	Alcohol consumption (measured by sales)	Yes	Not stated	Strong	SA, CON, TE, DR, CO, EX
	Stockwell 2013 (33)	Canada	Cross-section versus time series analysis of ecological data	Populations of 89 geographic areas in British Columbia	Actual minimum price increased over a 20 year period. 7Study modelled 10% increase in the average minimum price of all alcoholic	Alcohol-attributable hospital admissions	Yes	Not stated	Strong	SA, CON, TE, DR, PL, CO, EX

For peer review only - http://bmjopen.bmj.com/site/about/guidelines.xhtml

First author and year published	Study characteristics					Study assessment			Bradford Hill criteria met*
	Country	Study design	Population or participants	Pricing intervention studied	Outcomes studied	Peer-reviewe d	Conflict of interest	Quality rating	
				beverages					
Sutton 1995 (34)	UK	Cross-sectional survey	Participants in national General Household Survey, 1978-1990	National expenditure figures on alcohol price	Alcohol consumption	Yes	Not stated	Moderat e	CON, CO
Treisman 2010 (35)	Russia	Secondary analysis of historical data with focus on price changes 1990-1994	Population of Russia	Decrease in price of vodka in early 1990s - in 1993 real price of vodka was around 25% of that in 1990	Mortality	Yes	Not stated	Strong	SA, CON, TE, PL, CO, EX
Wald 1984 (36)	Poland	Analysis of routine data 1970-1981	Population of Poland	Poor harvest led to high prices, rationing and illegal sales	Alcohol consumption and alcohol-related hospital admissions	Yes	Not stated	Weak	CON, TE, PL, CO, EX
Wall 2013 (37)	New Zealand	Time series + econometri c modelling	Population of New Zealand	Real price (and also affordability)	Alcohol consumption	Yes	None	Moderat e	CON, CO (counter)
Zhao 2013 (38)	Canada	Cross-section versus time series	Populations of 16 Health Service Delivery Areas in British	Actual minimum price increased over a 20 year period. Study	Acute, chronic and wholly alcohol	Yes	None	Strong	SA, CON, TE, DR, PL, CO, EX

For peer review only - http://bmjopen.bmj.com/site/about/guidelines.xhtml

	First author and year published	Study characteristics					Study assessment			Bradford Hill criteria met*
		Country	Study design	Population or participants	Pricing intervention studied	Outcomes studied	Peer-reviewed	Conflict of interest	Quality rating	
			analysis of ecological data	Columbia, Canada	modelled 10% increase in the average minimum price of all alcoholic beverages. Also looked at outlet density	attributable mortality				
Systematic reviews	Wagenaar 2009 (39)	Worldwide	Systematic review and meta-analysis	Studies tended to cover general population	Alcohol price and taxation interventions studied together	Alcohol consumption (measured by alcohol sales or self-reported consumption)	Yes	None	Strong	AN
	Wagenaar 2010 (40)	Worldwide	Systematic review and meta-analysis	Studies tended to cover general population	Alcohol price and taxation interventions studied together	Alcohol-related morbidity (disease, injury, suicide, traffic crashes, sexually transmitted diseases, other drug use, crime and	Yes	Not stated	Strong	AN

For peer review only - http://bmjopen.bmj.com/site/about/guidelines.xhtml

	First author and year published	Study characteristics					Study assessment			Bradford Hill criteria met*
		Country	Study design	Population or participants	Pricing intervention studied	Outcomes studied	Peer-reviewe d	Conflict of interest	Quality rating	
						misbehaviour) and mortality				

*Abbreviations used for the Bradford Hill criteria: strength of the association (SA), consistency (CON), specificity (SP), temporality (TE), dose-response (DR), plausibility (PL), coherence (CO), experiment (EX), analogy (AN)

For peer review only - http://bmjopen.bmj.com/site/about/guidelines.xhtml

Table 3: Studies published in the grey literature included in Bradford Hill criteria assessment

	Study characteristics					Study assessment			Bradford Hill criteria met*
**Author and year published**	**Country**	**Study design**	**Population or participants**	**Pricing intervention studied**	**Outcomes studied**	**Peer-reviewed?**	**Conflict of interest?**	**Quality rating**	
Angus 2016 (41)	Scotland	Modelling study using SAPM	Scottish general population survey (subgroups of moderate, harmful, hazardous)	MUP of 30p, 40p, 50p, 60p and 70p, compared with taxation interventions	Alcohol consumption, consumer spending, exchequer and retail revenue, 47 health harms	Not stated	None	Strong	CON, SP, DR, PL, CO
Booth 2008 (42)	Worldwide	Review of reviews and systematic review	Studies tended to cover general population	Various minimum unit prices and taxation interventions	Alcohol consumption and various measures of alcohol harm	Yes	None	Strong	AN
Brennan 2008 (43)	England	Modelling study using SAPM	Adults in England	General price increases. MUP of £0.20, £0.25, £0.30, £0.35, £0.40, £0.45, £0.50, £0.60 and £0.70. Restrictions on off-trade price promotions.	Alcohol consumption, consumer spending, sales duty and VAT, 47 health harms, crime, and employment	Not stated	None	Strong	CON, SP, DR, PL, CO
Hill McManus 2012 (44)	Canada	Modelling study using SAPM	Adults in two Canadian provinces (Ontario and British Columbia)	MUP of C$1.50	Alcohol consumption, consumer spending, hospital admissions, mortality, crime	Not stated	None	Strong	CON, SP, PL, CO
Institute for Fiscal Studies	Great Britain	Economic modelling study	Shopping data from 25,248 British households	MUP of £0.45	Alcohol consumption	Not stated	Not stated	Not possible to rate	CON, SP, CO

For peer review only - http://bmjopen.bmj.com/site/about/guidelines.xhtml

	Study characteristics					Study assessment			Bradford Hill criteria met*
**Author and year published**	**Country**	**Study design**	**Population or participants**	**Pricing intervention studied**	**Outcomes studied**	**Peer-reviewed?**	**Conflict of interest?**	**Quality rating**	
2010 (45)		using market research data							
Institute for Fiscal Studies 2013 (46)	Great Britain	Economic analysis	Population of Great Britain	MUP of £0.45 and increased alcohol taxation	Alcohol consumption	Not stated	Not stated	Not possible to rate	CON, SP, CO
Meng 2010 (47)	Scotland	Modelling study using SAPM	Adults in Scotland	MUP of £0.20, £0.25, £0.30, £0.35, £0.40, £0.45, £0.50, £0.60 and £0.70. Restrictions on off-trade price promotions.	Alcohol consumption, consumer spending, 47 health harms, crime, employment	Not stated	None	Strong	CON, SP, DR, PL, CO

*Abbreviations used for the Bradford Hill criteria: strength of the association (SA), consistency (CON), specificity (SP), temporality (TE), dose-response (DR), plausibility (PL), coherence (CO), experiment (EX), analogy (AN)

For peer review only - http://bmjopen.bmj.com/site/about/guidelines.xhtml

Consideration against the Bradford Hill criteria for determining causality

1. Strength of the association

In 19/28 studies published in peer-reviewed journals, strength of the association between pricing and alcohol consumption or alcohol related harm was evidenced by a summary statistic such as an odds ratio, and by a test of the statistical significance of the association. As well as the statistical significance of the summary statistic, the magnitude of the effect was also considered, as a larger effect size corresponds to a greater population health impact. Studies in Canada found that 10% increases in minimum prices were associated with reductions in alcohol consumption of 10-16% (3,32), reductions in alcohol attributable hospital admissions of 9% (33), and reductions in alcohol-related mortality of 32%, each of which was statistically significant (38). Cross-sectional studies in the UK, Australia and New Zealand (14,17–20,25,29,30) and one trial from the USA (12) found statistically significant associations between cheaper alcohol and heavier drinking. The magnitude of the association varied across these studies but due to different study measures and outcomes the results are not all comparable. As an indication the odds ratio (OR) for buying cheap alcohol among heavier drinkers was 1.34 in Crawford's study (19) and 1.70 in Callinan's study (17), and in Casswell's study the odds of being a heavier drinker were roughly double among cheap alcohol purchasers (18). Other studies however, had significant results in subgroups only or for particular outcomes (e.g. (16), (31)), and there was not any evidence to support this criterion from the grey literature. Overall there is reasonably good support for the strength of the association criterion.

2. Consistency

This criterion requires looking across all the studies included in the review to see whether similar conclusions have been drawn. Inverse associations between alcohol pricing and alcohol consumption or harm have been documented in countries in Europe, North America and Australia, and although most studies are from the last 10 years, there are studies from the 1970s and 1980s as well. There is evidence from different research teams and different types of study including cross-sectional studies, time series analyses and econometric modelling studies. Support for the consistency criterion is very strong.

3. Specificity

The specificity criterion relates to whether changes in alcohol consumption or harm could be attributed to anything other than the price intervention. Many studies included have statistically adjusted for confounding factors, however the best support for the specificity criterion comes from the econometric modelling studies because there is no risk of residual confounding. The Sheffield Alcohol Policy Model is one such model and has been applied in England (15,24,26,27,43), Scotland (41,47) and Canada (44) and provides very strong support for the specificity criterion. Further support is provided by other different modelling studies in the UK (45,46) and a trial in the US (12). Thus support for the specificity criteria is very strong.

4. Temporality

It is important that price changes or price interventions take place before changes to alcohol consumption and harm in order to attribute causality. Strong support for this criterion comes from research following the introduction of MUP in Canada, where price increases preceded reductions in

For peer review only - http://bmjopen.bmj.com/site/about/guidelines.xhtml

alcohol consumption (3,32), alcohol attributable hospital admissions (33), and alcohol-related mortality (38). Studies where price changes preceded the expected changes in alcohol consumption or harm have also been conducted in Russia (13,35), Poland (36), and Finland (23). Overall there is very strong support for the temporality criterion.

5. Dose-response/biological gradient

This criterion is supported if different price levels have been found to have differing effects on consumption or harm. Many of the studies using the Sheffield Alcohol Policy Model explore the impact of a range of potential MUP options (15,26,27,41,47), and these consistently suggest that the higher the MUP, the greater the reductions in alcohol consumption or alcohol-related harms. The Canadian studies of minimum pricing lend further support for this criterion because the analysis presents the effect on consumption or harm of a modelled 1% increase in price, meaning dose-response can be inferred (3,32,33,38). Dose response is supported to a lesser extent by evidence from cross-sectional studies that heavier drinkers pay less for their alcohol (14,17,29,30), that weekly drinking is more prevalent in adolescents living in countries where alcohol is cheaper (22). Overall, there is strong support for the dose-response criterion, although the relationship is difficult to quantify.

6. Biological plausibility

This criterion addresses the effects of alcohol. That there is a relationship between alcohol consumption and harm is indisputable, although there is some debate about beneficial health effects of moderate drinking. Alcohol consumption causes many different physical and psychological conditions and these can be acute or chronic. See (48) for a comprehensive list of acute and chronic adverse health effects of alcohol. There is evidence from 11/28 research studies and 4/7 studies in the grey literature that the price of alcohol is inversely related to alcohol-related morbidity, hospital admissions, or mortality. This provides strong support for the plausibility criterion.

7. Coherence

This criterion refers to whether studies from different disciplines have had complementary findings and whether these fit or ‘hang’ well together. It is different to consistency, which is more concerned with reproducibility of findings. The findings of the majority of studies supported the coherence criterion in that they suggest that real-world minimum unit pricing (e.g. (3,32,33,38)) or price increases (e.g. (13,23,36)) led to reductions in alcohol consumption and alcohol-related harm, modelling studies suggest heavier drinkers will be most affected by MUP (e.g. (24)), and cross sectional surveys find that it is the heavier drinkers that are drinking the cheapest alcohol (e.g. (14,30)). There are however a small number of studies which had counter findings; an Australian study found that high intensity drinking was not affected by higher prices (16) and a worldwide study of adolescents found no significant effect of price on drunkenness (22). Two studies provided weak support for MUP in finding that affordability had a greater influence than price (37), and that only a small number of specific alcohol-related harms were actually affected by price (31). Although the support for the coherence criterion was not unanimous, overall the evidence base provides strong support for this criterion.

8. Experiment

For peer review only - http://bmjopen.bmj.com/site/about/guidelines.xhtml

We have not identified any randomised controlled trials of minimum pricing or price-based interventions to reduce alcohol consumption. There is a small (and not randomised) trial from the 1970s (12) which found participants living in controlled conditions and offered a daily 'Happy Hour' discount drank significantly more alcohol than those who were not offered the discount. There is however substantial evidence in support of the experiment criterion from time series analyses or natural experiments, for example: where minimum pricing was introduced in Canada (3,32,33,38) and where prices fluctuated in the late 1980s and early 1990s in Russia (13,35), and to a lesser extent in Finland, where price increases were associated with reduced mortality only among men with a basic education (23). These studies provide tentative support for the experiment criterion.

9. Analogy

To address the analogy criterion areas related to alcohol pricing must be considered. There is evidence from literature on the affordability of alcohol (49) that consumption and harm are very responsive to the affordability of alcohol. Large systematic reviews have investigated the price elasticity of demand for alcohol (50), and have found that higher alcohol pricing and taxation (considered together) are associated with reductions in alcohol consumption, alcohol-related morbidity and mortality (39,40,42). There are a number of arguments favouring minimum pricing as an intervention over increased taxation. One of these is that increases in taxation can sometimes be absorbed by retailers or only 'passed-through' to consumers of more expensive products, keeping cheap alcohol at a low price (51). Overall the support for the analogy criterion is very strong, although Bradford Hill describes this as the weakest evidence for causality.

DISCUSSION

We assessed 28 research studies and two systematic reviews, plus a further seven studies from the grey literature in this review of the evidence for priced based interventions – such as MUP - to reduce alcohol consumption and alcohol-related harm. All nine of the Bradford Hill criteria for causality were met. However, the evidence for two of the criteria, although present, was not as strong as it was for the other criteria. These criteria were strength of the association (criterion 1) and experiment (criterion 8), and according to Bradford Hill, these are the two criteria that can provide the strongest evidence for causality. There were also a small minority of studies that found price-based interventions to be only minimally effective or effective only in population subgroups. Therefore although all of the criteria were supported, we conclude that it is highly probable, but not definite, that introducing MUP would reduce alcohol consumption and alcohol-related harms.

Strengths of this study are that this is the first to have systematically reviewed the literature relevant specifically to alcohol minimum pricing policies. We had broad inclusion criteria with regards to study design, price intervention and outcome measure, allowing for a comprehensive review of the evidence base. Application of the Bradford Hill criteria as part of a narrative systematic literature review is a useful and emergent technique for identifying causality: a PubMed search for systematic reviews with Bradford Hill' mentioned in the title or abstract yielded 28 results, 90% of which were published in the last five years. The limitations of this systematic review relate mainly to the broad range of studies included. It was not possible to conduct any kind of meta-analysis and therefore we do not present a pooled estimate for the likely effect of MUP on certain outcomes. There were also challenges with the quality appraisal. The EPHPP quality assessment tool was used to assess studies. However it was not possible to appraise two of the studies from the grey literature using this tool,

For peer review only - http://bmjopen.bmj.com/site/about/guidelines.xhtml

and there were some challenges assessing the econometric modelling studies against this framework. However overall we think that our quality appraisal across the different studies is broadly comparable. It should also be noted that although a number of studies were rated as 'strong', this is in relation to their respective study designs and does not reflect the position of the study type in the hierarchy of evidence framework.

This is the first systematic review that has addressed the effectiveness of price-based interventions for alcohol such as MUP using the Bradford Hill criteria. Previous systematic reviews of alcohol price and consumption (39) and alcohol-related harm (40) have tended to consider the effect of price increases and increased taxation together. These reviews found significant effects on consumption and morbidity and mortality. Although price regulation and taxation are closely related policy options, evidence from surveys (51) and modelling studies (41) suggests that the effects of each are somewhat different, and this is why we chose to study only price in this review. It is important to highlight that a considerable proportion of included studies were produced by a small number of research teams. Also, with regards to the econometric modelling studies, uncertainty in estimates or forecasts is often poorly communicated outside of the academic literature. The overall risk of bias in the included studies was minimised by excluding studies with a conflict of interest (either for or against MUP). It was not possible to assess publication bias using an analytical technique such as a funnel plot due to the narrative nature of the review, however we anticipate that by including grey literature in this review we have mitigated publication bias as far as reasonably possible.

Overall the findings of this review lend strong support for policies such as MUP in reducing alcohol consumption and alcohol-related harm, with all nine of the Bradford Hill criteria met and little by way of counter findings. As it is unlikely to be feasible to conduct randomised controlled trials (RCTs) of MUP, the decision whether or not to introduce MUP will not be based on a systematic review and meta-analysis of RCTs, and therefore this synthesis of the evidence base according to the Bradford Hill criteria is of value.

Unanswered questions about the effectiveness of MUP remain, and in Scotland in particular, there are opportunities to address these. The decision on whether to introduce MUP in Scotland currently lies with the Scottish Courts of Session in Edinburgh (NB to be updated as situation progresses throughout the summer). If Scotland rules to implement MUP, then it would be possible to evaluate the validity of the Sheffield Alcohol Policy Model studies conducted using Scottish data. It would also be possible to conduct a longitudinal study to evaluate the effectiveness of MUP in reducing alcohol consumption and alcohol-related morbidity and mortality. The findings of this natural experiment would have relevance elsewhere within and outside the UK.

ACKNOWLEDGEMENTS

We would like to thank Rebecca McDonald for advice on using the Bradford Hill criteria in a systematic review, Dr James Nicholls for advice on study interpretation, and Dr Daniel Stahl for statistical advice on some of the included studies.

CONTRIBUTIONS

SM conceived the idea. SB conducted the initial search. SM and SM contributed to independently reviewing abstracts, hand-searching reference lists, completing data extraction, and conducting

For peer review only - http://bmjopen.bmj.com/site/about/guidelines.xhtml

quality appraisal. All authors contributed to the analysis and interpretation of the results and contributed to writing the manuscript. SB is guarantor.

FUNDING

This research received no specific grant from any funding agency in the public, commercial or not-for-profit sectors.

COMPETING INTERESTS

All authors have completed the ICMJE uniform disclosure form at www.icmje.org/coi_disclosure.pdf and declare: no support from any organisation for the submitted work; no financial relationships with any organisations that might have an interest in the submitted work in the previous three years; no other relationships or activities that could appear to have influenced the submitted work.

Two of the authors work at King's College London, which as an institution is listed as a member of the Alcohol Health Alliance. SM has received funding indirectly from UKCTAS, which as an institution is also listed as a member of the Alcohol Health Alliance. However none of the authors have any relationship with the Alcohol Health Alliance.

DATA SHARING

There is no dataset or any unpublished data relating to this systematic review.

For peer review only

For peer review only - http://bmjopen.bmj.com/site/about/guidelines.xhtml

REFERENCES

1. Lifestyles Statistics Team,, Health and Social Care Information Centre. Statistics on Alcohol. England, 2015 [Internet]. 2015 [cited 2016 Jun 3]. Available from: http://www.hscic.gov.uk/catalogue/PUB17712/alc-eng-2015-rep.pdf

2. Anderson P, Chisholm D, Fuhr DC. Effectiveness and cost-effectiveness of policies and programmes to reduce the harm caused by alcohol. Lancet Lond Engl. 2009 Jun 27;373(9682):2234–46.

3. Stockwell T, Auld MC, Zhao J, Martin G. Does minimum pricing reduce alcohol consumption? The experience of a Canadian province. Addiction. 2012 May 1;107(5):912–20.

4. World Health Organization. European status report on alcohol and health 2014. Pricing policies [Internet]. World Health Organization; 2014. Available from: http://www.euro.who.int/__data/assets/pdf_file/0003/244902/Pricing-policies.pdf

5. HM Government. The Government's Alcohol Strategy [Internet]. 2012 [cited 2016 Jun 3]. Available from: https://www.gov.uk/government/uploads/system/uploads/attachment_data/file/224075/alcohol-strategy.pdf

6. Home Office. Guidance on banning the sale of alcohol below the cost of duty plus VAT. For suppliers of alcohol and enforcement authorities in England and Wales [Internet]. 2015 [cited 2016 Jun 3]. Available from: https://www.gov.uk/government/uploads/system/uploads/attachment_data/file/415522/HO_Guidance_on_BBCS.pdf

7. Off Licence News. First conviction for selling alcohol "below cost" sees retailer fined [Internet]. 2016 [cited 2016 Jun 3]. Available from: http://www.offlicencenews.co.uk/news/fullstory.php/aid/15750/First_conviction_for_selling_alcohol__below_cost__sees_retailer_fined.html

8. Home Office. Duty plus VAT permitted price calculator (2015) [Internet]. 2016. Available from: https://www.gov.uk/government/publications/banning-the-sale-of-alcohol-below-the-cost-of-duty-plus-vat-march-2015

9. Fitzgerald N, Angus C. Four Nations: How Evidence-based are Alcohol Policies and Programmes across the UK?. London: [Internet]. London: Alliance for Useful Evidence/Alcohol Health Alliance.; 2015. Available from: http://www.alliance4usefulevidence.org/assets/Four-Nations-v3.pdf

10. Cochrane Handbook. 21.4 Assessment of study quality and risk of bias [Internet]. [cited 2016 Jun 2]. Available from: http://handbook.cochrane.org/chapter_21/21_4_assessment_of_study_quality_and_risk_of_bias.htm

11. Hill AB. The Environment and Disease: Association or Causation? Proc R Soc Med. 1965 May;58(5):295–300.

12. Babor TF, Mendelson JH, Greenberg I, Kuehnle J. Experimental analysis of the 'happy hour": effects of purchase price on alcohol consumption. Psychopharmacology (Berl). 1978 Jun 15;58(1):35–41.

For peer review only

For peer review only - http://bmjopen.bmj.com/site/about/guidelines.xhtml

13. Bhattacharya J, Gathmann C, Miller G. The Gorbachev Anti-Alcohol Campaign and Russia's Mortality Crisis. Am Econ J Appl Econ. 2013;5(2):232–60.

14. Black H, Gill J, Chick J. The price of a drink: levels of consumption and price paid per unit of alcohol by Edinburgh's ill drinkers with a comparison to wider alcohol sales in Scotland. Addiction. 2011 Apr 1;106(4):729–36.

15. Brennan A, Meng Y, Holmes J, Hill-McManus D, Meier PS. Potential benefits of minimum unit pricing for alcohol versus a ban on below cost selling in England 2014: modelling study. The BMJ. 2014 Sep 30;349:g5452.

16. Byrnes J, Shakeshaft A, Petrie D, Doran C. Can harms associated with high-intensity drinking be reduced by increasing the price of alcohol? Drug Alcohol Rev. 2013 Jan;32(1):27–30.

17. Callinan S, Room R, Livingston M, Jiang H. Who Purchases Low-Cost Alcohol in Australia? Alcohol Alcohol. 2015 Jun 24;agv066.

18. Casswell S, Huckle T, Wall M, Yeh LC. International alcohol control study: pricing data and hours of purchase predict heavier drinking. Alcohol Clin Exp Res. 2014 May;38(5):1425–31.

19. Crawford MJ, Parry AMH, Weston ARW, Seretis D, Zauter-Tutt M, Hussain A, et al. Relationship between price paid for off-trade alcohol, alcohol consumption and income in England: a cross-sectional survey. Alcohol Alcohol Oxf Oxfs. 2012 Dec;47(6):738–42.

20. Falkner C, Christie G. The effect of alcohol price on dependent drinkers' alcohol consumption. N Z Med J. 2015;128(1427).

21. Forsyth AJM, Ellaway A, Davidson N. How Might the Alcohol Minimum Unit Pricing (MUP) Impact upon Local Off-Sales Shops and the Communities Which They Serve? Alcohol Alcohol. 2014 Jan 1;49(1):96–102.

22. Gilligan C, Kuntsche E, Gmel G. Adolescent drinking patterns across countries: associations with alcohol policies. Alcohol Alcohol Oxf Oxfs. 2012 Dec;47(6):732–7.

23. Herttua K, Mäkelä P, Martikainen P. Minimum Prices for Alcohol and Educational Disparities in Alcohol-related Mortality. Epidemiology. 2015 May;26(3):337–43.

24. Holmes J, Meng Y, Meier PS, Brennan A, Angus C, Campbell-Burton A, et al. Effects of minimum unit pricing for alcohol on different income and socioeconomic groups: a modelling study. The Lancet. 2014 May 16;383(9929):1655–64.

25. Ludbrook PA, Petrie D, McKenzie L, Farrar S. Tackling alcohol misuse: Purchasing patterns affected by minimum pricing for alcohol. Appl Health Econ Health Policy. 2012 Aug 6;10(1):51–63.

26. Meier PS, Purshouse R, Brennan A. Policy options for alcohol price regulation: the importance of modelling population heterogeneity. Addiction. 2010 Mar 1;105(3):383–93.

27. Purshouse RC, Meier PS, Brennan A, Taylor KB, Rafia R. Estimated effect of alcohol pricing policies on health and health economic outcomes in England: an epidemiological model. The Lancet. 2010 Apr;375(9723):1355–64.

For peer review only - http://bmjopen.bmj.com/site/about/guidelines.xhtml

28. Seaman P, Edgar F, Ikegwuonu T. The role of alcohol price in young adult drinking cultures in Scotland. Drugs Educ Prev Policy. 2013 Aug 1;20(4):278–85.

29. Sharma A, Vandenberg B, Hollingsworth B. Minimum Pricing of Alcohol versus Volumetric Taxation: Which Policy Will Reduce Heavy Consumption without Adversely Affecting Light and Moderate Consumers? PLoS ONE. 2014 Jan 22;9(1):e80936.

30. Sheron N, Chilcott F, Matthews L, Challoner B, Thomas M. Impact of minimum price per unit of alcohol on patients with liver disease in the UK. Clin Med. 2014 Aug 1;14(4):396–403.

31. Sloan FA, Reilly BA, Schenzler C. Effects of prices, civil and criminal sanctions, and law enforcement on alcohol-related mortality. J Stud Alcohol. 1994 Jul;55(4):454–65.

32. Stockwell T, Zhao J, Giesbrecht N, Macdonald S, Thomas G, Wettlaufer A. The Raising of Minimum Alcohol Prices in Saskatchewan, Canada: Impacts on Consumption and Implications for Public Health. Am J Public Health. 2012 Oct 18;102(12):e103–10.

33. Stockwell T, Zhao J, Martin G, Macdonald S, Vallance K, Treno A, et al. Minimum Alcohol Prices and Outlet Densities in British Columbia, Canada: Estimated Impacts on Alcohol-Attributable Hospital Admissions. Am J Public Health. 2013 Apr 18;103(11):2014–20.

34. Sutton M, Godfrey C. A grouped data regression approach to estimating economic and social influences on individual drinking behaviour. Health Econ. 1995 May 1;4(3):237–47.

35. Treisman D. Death and prices. Econ Transit. 2010 Apr 1;18(2):281–331.

36. Wald I, Moskalewicz J. Alcohol policy in a crisis situation. Br J Addict. 1984 Sep;79(3):331–5.

37. Wall M, Casswell S. Affordability of alcohol as a key driver of alcohol demand in New Zealand: a co-integration analysis. Addict Abingdon Engl. 2013 Jan;108(1):72–9.

38. Zhao J, Stockwell T, Martin G, Macdonald S, Vallance K, Treno A, et al. The relationship between minimum alcohol prices, outlet densities and alcohol-attributable deaths in British Columbia, 2002–09. Addiction. 2013 Jun 1;108(6):1059–69.

39. Wagenaar AC, Salois MJ, Komro KA. Effects of beverage alcohol price and tax levels on drinking: a meta-analysis of 1003 estimates from 112 studies. Addict Abingdon Engl. 2009 Feb;104(2):179–90.

40. Wagenaar AC, Tobler AL, Komro KA. Effects of alcohol tax and price policies on morbidity and mortality: a systematic review. Am J Public Health. 2010 Nov;100(11):2270–8.

41. Angus C, Holmes J, Pryce R, Meier P, Brennan A. Model-based appraisal of the comparative impact of Minimum Unit Pricing and taxation policies in Scotland An adaptation of the Sheffield Alcohol Policy Model version 3 [Internet]. ScHARR, University of Sheffield; 2016 Apr [cited 2016 Apr 7]. Available from: https://www.shef.ac.uk/polopoly_fs/1.565373!/file/Scotland_report_2016.pdf

42. Booth A, Brennan A, Meier PS, O'Reilly D, Purshouse R, Stockwell T, et al. Independent review of the effects of alcohol pricing and promotion: part A – systematic reviews. Project Report for the Department of Health September 2008. ScHARR University of Sheffield; 2008.

For peer review only

For peer review only - http://bmjopen.bmj.com/site/about/guidelines.xhtml

43. Brennan A, Purshouse R, Taylor K, Rafia R. Independent review of the effects of alcohol pricing and promotion: part B. Modelling the Potential Impact of Pricing and Promotion Policies for Alcohol in England: Results from the Sheffield Alcohol Policy Model Version 2008(1-1) [Internet]. ScHARR University of Sheffield; 2008. Available from: https://www.shef.ac.uk/polopoly_fs/1.95621!/file/PartB.pdf

44. Hill McManus D, Brennan A, Stockwell T, Giesbrecht N, Thomas G, Zhao J, et al. Model-based appraisal of alcohol minimum pricing in Ontario and British Columbia: A Canadian adaptation of the Sheffield Alcohol Policy Model Version 2 [Internet]. 2012. Available from: http://www.uvic.ca/research/centres/carbc/assets/docs/report-model-based-appraisal.pdf

45. Institute for Fiscal Studies. The Impact of Introducing a Minimum Price on Alcohol in Britain. IFS Briefing Note BN109. 2010.

46. Institute for Fiscal Studies. Price-based measures to reduce alcohol consumption. IFS Briefing Note BN138. 2013.

47. Meng Y, Purshouse R, Brennan A, Meier PS. Model-based appraisal of alcohol minimum pricing and off-licensed trade discount bans in Scotland using the Sheffield Alcohol Policy Model (v.2): Second update based on newly available data [Internet]. 2010. Available from: http://www.shef.ac.uk/polopoly_fs/1.96510!/file/scotlandupdate.pdf

48. Department of Health. Table 1.1 Acute adverse effects associated with the use of alcohol and Table 1.2 Chronic adverse effects associated with the use of alcohol. In A summary of the health harms of drugs [Internet]. Leeds: Department of Health; 2011 [cited 2016 May 24]. Available from: http://www.nta.nhs.uk/uploads/healthharmsfinal-v1.pdf

49. Rabinovich L, Brutscher P-B, de Vries H, Tiessen J, Clift J, Reding A. The affordability of alcoholic beverages in the European Union. 2012;

50. Gallet CA. The Demand for Alcohol: A Meta-Analysis of Elasticities [Internet]. Rochester, NY: Social Science Research Network; 2007 May [cited 2016 Jan 6]. Report No.: ID 985689. Available from: http://papers.ssrn.com/abstract=985689

51. Ally AK, Meng Y, Chakraborty R, Dobson PW, Seaton JS, Holmes J, et al. Alcohol tax pass-through across the product and price range: do retailers treat cheap alcohol differently? Addiction. 2014 Dec 1;109(12):1994–2002.

For peer review only

For peer review only - http://bmjopen.bmj.com/site/about/guidelines.xhtml

PRISMA 2009 Flow Diagram

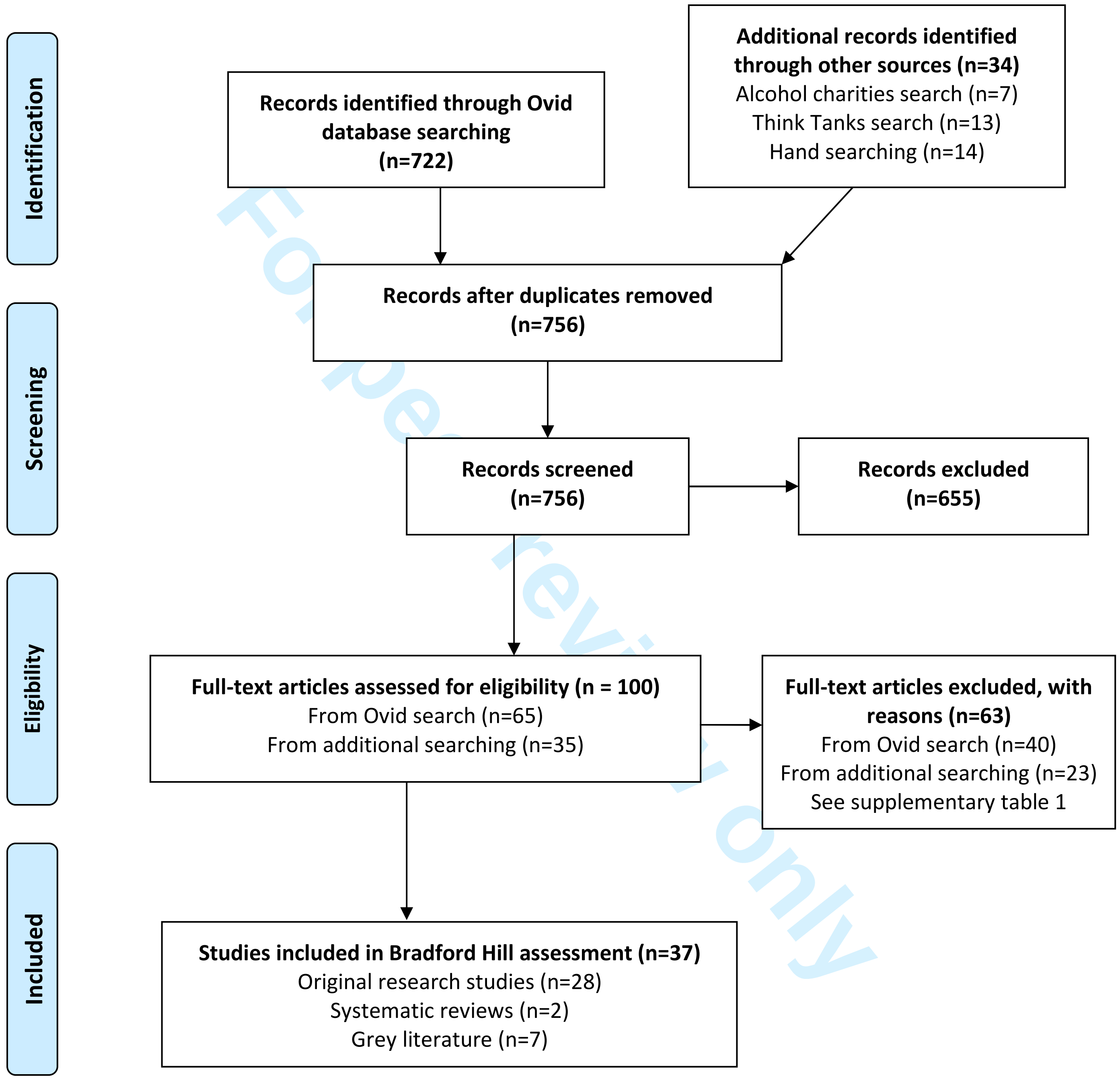

From: Moher D, Liberati A, Tetzlaff J, Altman DG, The PRISMA Group (2009). *P*referred *R*eporting *I*tems for *S*ystematic Reviews and *M*eta-*A*nalyses: The PRISMA Statement. PLoS Med 6(6): e1000097. doi:10.1371/journal.pmed1000097

For more information, visit www.prisma-statement.org.

For peer review only - http://bmjopen.bmj.com/site/about/guidelines.xhtml

Online supplementary table: identified studies where full text assessed, then not included in review

Source	Author and year published	Study type	Reason excluded
Ovid search	Aage 2012 (1)	Time series analysis	Affordability, not price
	Ayyagari 2013 (2)	Econometric analysis of price and consumption data	Price elasticities of demand only
	Bellis 2009 (3)	Cross-sectional surveys	Association between drink type and price, and drink type and harms, but not directly reported in paper
	Bennetts 2008 (4)	Review (not systematic)	Review of a report (not a research study)
	Callinan 2015 (5)	Editorial (not a research study)	Editorial about different pricing, taxation and afforability measures, not a research study
	Chalmers 2013 (6)	Commentary (not a research study)	Is a commentary about challenges to MUP e.g. industry and the law
	Chaloupka 2002 (7)	Review (not systematic)	Review of previous economic studies (not systematic)
	Chick 2012 (8)	Editorial (not a research study)	Is about bans on multi-buys, not minimum price
	Cook 2014 (9)	Analysis of cross-sectional survey data and alcohol policies	Affordability not price (GDP PPP)
	Duffy 1981 (10)	Econometric time series analysis	Talks about elasticities in relation to duty rises not MUP. NB is M duffy, not J duffy
	Giesbrecht 2003 (11)	Review (not systematic)	Review of previous studies (not systematic)
	Giesbrecht 2015 (12)	Policy analysis	Does not relate price intervention to consumption or harm
	Grossman 2004 (13)	Economic analysis	Tax (federal excise tax increase) not MUP
	Hadland 2015 (14)	Policy analysis	Effects of numerous alcohol policies considered together
	Heeb 2003 (15)	Natural experiment: longitudinal survey 3 months before and 3 months after price change	Tax, not price intervention
	Hogan 2006 (16)	Natural experiment/evaluation	Tax, not price intervention

For peer review only - http://bmjopen.bmj.com/site/about/guidelines.xhtml

	Jónsson 2013 (17)	Cross-sectional surveys and routine data	Policies included tax and other supply side policies, but not MUP
	Katikireddi 2012 (18)	Commentary (not a research study)	Is a commentary about legal considerations of MUP, not effectiveness
	Katikireddi 2014 (19)	Qualitative study	Stakeholder views of MUP, not effectiveness
	Khaltourina 2015 (20)	Cross-sectional surveys and routine data	Tax, not price intervention
	Knibbe 2014 (21)	Analysis of cross-sectional survey data and alcohol policies	No price intervention studied, price investigated more as a covariate
	Lindeman 2013 (22)	Cross-sectional surveys and routine data	Tax, not price intervention
	Lonsdale 2012 (23)	Qualitative study	Public opinion of policy, nothing about effectiveness
	Mäkelä 2009 (24)	Review (not systematic) and analysis of routine data	Tax, not price intervention
	McCambridge 2014 (25)	Document analysis and qualitative interviews	Corporate lobbying, not the effectiveness of MUP
	Meng 2014 (26)	Econometric analysis using pseudo panel	Price elasticities of demand only
	Nelson 2013 (27)	Review (not systematic)	Conflict of interest (funded by International Center for Alcohol Policies).
	Nelson 2014 (affordability) (28)	Analysis of routine data and economic modelling	Affordability not price, and conflict of interest (funded by International Center for Alcohol Policies).
	Nelson 2014 (elasticities) (29)	Economic modelling	Conflict of interest (funded by International Center for Alcohol Policies).
	Nelson 2014 (gender) (30)	Systematic review	Conflict of interest (funded by International Center for Alcohol Policies).
	Nicholls & Greenaway 2015 (31)	Policy analysis	Is about policy framing, not the effectiveness of MUP
	Record 2009 (32)	Modelling study	Conflict of interest – both authors were members of the Alcohol Health Alliance which is campaigning for MUP
	Rush 1986 (33)	Analysis of routine data	Affordability, not price
	Shi 2011 (34)	Econometric analysis of price and	Price elasticities of demand only

For peer review only - http://bmjopen.bmj.com/site/about/guidelines.xhtml

		consumption data	
	Skov 2011 (35)	Natural experiment	Tax, not price intervention
	Stockwell 2012 (36)	Letter to journal (not a research study)	Letter concerning homelessness and price paid for alcohol
	Stockwell 2013 (37)	Letter to journal (not a research study)	Letter to editor about UK industry criticism of Canadian research on MUP, not a research study
	Tian 2011 (38)	Econometric analysis of price and consumption data	Price elasticities of demand only
	Xuan 2015 (39)	Analysis of cross-sectional survey data and alcohol policies	Price and tax considered together
	Yashkin 2013 (40)	Econometric analysis of price and consumption data	Price elasticities of demand only
Hand searching & grey literature	Ally 2014 (41)	Analysis of VAT and price data	Tax, not price intervention
	Anderson 2009 (42)	Lancet 'Series' article – a commentary/review	Not an original research study.
	Centre for Economics and Business Research 2010 (43)	Report (not a research study)	Conflict of interest (funded by SAB Miller). Is a critique of Sheffield modelling, not a research study.
	Craven 2013 (44)	Economic report	Published in the journal of the Institute of Economic Affairs, which has a conflict of interest.
	Duffy & Snowdon 2012 (45)	Report chapters (not a research study)	Is a critique of Sheffield modelling, not a research study.
	Fitzgerald & Angus 2015 (46)	Report (not a research study)	Is a report about use of evidence in policymaking, not about effectiveness of MUP. Also not a research study.
	Gallet 2007 (47)	Meta-analysis	Is looking at elasticities of demand for alcohol, not the effectiveness of MUP.
	Gray 2000 (48)	Natural experiement	Restrictions do not include any price interventions
	Gruenewald 2006 (49)	Time series analysis/modelling	Tax, not price intervention
	Hilton 2014 (50)	Content analysis of UK newsprint	Media representations of MUP, not effectiveness

For peer review only - http://bmjopen.bmj.com/site/about/guidelines.xhtml

	Home Office 2011 (51)	'Summary review'/report (not a research study)	Replicates what we have elsewhere, but in less detail. Also not a research study.
	Institute for Fiscal Studies 2011 (52)	Report (not a research study)	Is not about effectiveness of MUP
	Institute for Fiscal Studies 2013 (53)	Report (not a research study)	Is not looking at effectiveness of MUP, is comparing Govt and industry revenue under MUP and higher tax
	Institute of Alcohol Studies (Goodliffe) 2014 (54)	Webpage	Is discussing legal issues, not effectiveness of MUP
	Kisely & Lawence 2015 (55)	Natural experiment	Tax, not price intervention
	Ludbrook 2010 (56)	Secondary analysis of Expenditure and Food Survey	Describes purchasing patterns of low price alcohol in Scotland. Not about MUP.
	Ornstein 1983 (57)	Literature review book chapter (not systematic)	Price elasticities of demand only
	Rabinovch 2012 (58)	Technical report (contains data)	Does not assess effectiveness of MUP
	Radaev 2015 (59)	Time series analysis	Paper explored the effect of price interventions on consumption of homemade alcohol, not the effectiveness of MUP
	Robinson 2013 (60)	Letter to journal (not a research study)	Letter to editor about evidence for MUP, not a research study
	Snowdon 2015 (61)	Book chapter	Is a critique of Sheffield modelling, not a research study. Institute of Economic Affairs also has a conflict of interest.
	Wine and Spirits Trade Association n.d. (62)	Press release	Press release critiquing Zhao 2013 paper, not a research study
	World Health Organisation 2014 (63)	Report	Does not discuss effectiveness of MUP

For peer review only - http://bmjopen.bmj.com/site/about/guidelines.xhtml

References

1. Aage H. Alcohol in Greenland 1951–2010: consumption, mortality, prices. Int J Circumpolar Health [Internet]. 2012 Dec 17 [cited 2016 Feb 27];71. Available from: http://www.ncbi.nlm.nih.gov/pmc/articles/PMC3525923/

2. Ayyagari P, Deb P, Fletcher J, Gallo W, Sindelar JL. Understanding heterogeneity in price elasticities in the demand for alcohol for older individuals. Health Econ. 2013 Jan;22(1):89–105.

3. Bellis MA, Phillips-Howard PA, Hughes K, Hughes S, Cook PA, Morleo M, et al. Teenage drinking, alcohol availability and pricing: a cross-sectional study of risk and protective factors for alcohol-related harms in school children. BMC Public Health. 2009 Oct 9;9(1):1–12.

4. Bennetts R. Russell Bennetts. Alcohol: Price, Policy and Public Health. Alcohol Alcohol. 2008 Mar 1;43(2):123–123.

5. Callinan S, Room R, Dietze P. Alcohol Price Policies as an Instrument of Health Equity: Differential Effects of Tax and Minimum Price Measures. Alcohol Alcohol Oxf Oxfs. 2015 Nov;50(6):629–30.

6. Chalmers J, Carragher N, Davoren S, O'Brien P. Real or perceived impediments to minimum pricing of alcohol in Australia: Public opinion, the industry and the law. Int J Drug Policy. 2013 Nov;24(6):517–23.

7. Chaloupka FJ, Grossman M, Saffer H. The effects of price on alcohol consumption and alcohol-related problems. Alcohol Res Health J Natl Inst Alcohol Abuse Alcohol. 2002;26(1):22–34.

8. Chick J. 16 for the Price of 10: Effects of a Ban on Multi-Buy Alcohol. Alcohol Alcohol. 2012 Feb 22;47(2):83–83.

9. Cook WK, Bond J, Greenfield TK. Are alcohol policies associated with alcohol consumption in low- and middle-income countries? Addict Abingdon Engl. 2014 Jul;109(7):1081–90.

10. Duffy M. The influence of prices, consumer incomes and advertising upon the demand for alcoholic drink in the United Kindgom: an econometric study. Alcohol Alcohol. 1981 Sep 21;16(4):200–9.

11. Giesbrecht N, Greenfield TK. Preventing Alcohol-Related Problems in the US Through Policy: Media Campaigns, Regulatory Approaches and Environmental Interventions. J Prim Prev. 2003 Sep;24(1):63–104.

12. Giesbrecht N, Wettlaufer A, Thomas G, Stockwell T, Thompson K, April N, et al. Pricing of alcohol in Canada: A comparison of provincial policies and harm-reduction opportunities. Drug Alcohol Rev. 2015 Nov 4;

13. Grossman M. Individual Behaviors and Substance Use: The Role of Price [Internet]. National Bureau of Economic Research; 2004 Dec [cited 2016 Feb 27]. Report No.: 10948. Available from: http://www.nber.org/papers/w10948

For peer review only - http://bmjopen.bmj.com/site/about/guidelines.xhtml

14. Hadland SE, Xuan Z, Blanchette JG, Heeren TC, Swahn MH, Naimi TS. Alcohol Policies and Alcoholic Cirrhosis Mortality in the United States. Prev Chronic Dis [Internet]. 2015 Oct 15 [cited 2016 Feb 27];12. Available from: http://www.cdc.gov/pcd/issues/2015/15_0200.htm

15. Heeb J-L, Gmel G, Zurbrügg C, Kuo M, Rehm J. Changes in alcohol consumption following a reduction in the price of spirits: a natural experiment in Switzerland. Addict Abingdon Engl. 2003 Oct;98(10):1433–46.

16. Hogan E, BOFFA J, ROSEWARNE C, BELL S, CHEE DA. What price do we pay to prevent alcohol-related harms in Aboriginal communities? The Alice Springs trial of liquor licensing restrictions. Drug Alcohol Rev. 2006;25(3):207–12.

17. Jónsson RM, Kristjánsson S. Alcohol policy and public opinion in Iceland, 1989–2012. Nord Stud Alcohol Drugs. 2013;30(6):539–49.

18. Katikireddi SV, McLean JA. Introducing a minimum unit price for alcohol in Scotland: considerations under European Law and the implications for European public health. Eur J Public Health. 2012 Aug 1;22(4):457–8.

19. Katikireddi SV, Bond L, Hilton S. Perspectives on econometric modelling to inform policy: a UK qualitative case study of minimum unit pricing of alcohol. Eur J Public Health. 2014 Jun 1;24(3):490–5.

20. Khaltourina D, Korotayev A. Effects of Specific Alcohol Control Policy Measures on Alcohol-Related Mortality in Russia from 1998 to 2013. Alcohol Alcohol. 2015 Sep 1;50(5):588–601.

21. Knibbe RA, Derickx M, Allamani A, Massini G. Alcohol Consumption and its Related Harms in the Netherlands Since 1960: Relationships With Planned and Unplanned Factors. Subst Use Misuse. 2014 Oct 15;49(12):1589–600.

22. Lindeman M, Karlsson T, Österberg E. Public opinions, alcohol consumption and policy changes in Finland, 1993–2013. Nord Stud Alcohol Drugs. 2013;30(6):507–24.

23. Lonsdale AJ, Hardcastle SJ, Hagger MS. A minimum price per unit of alcohol: A focus group study to investigate public opinion concerning UK government proposals to introduce new price controls to curb alcohol consumption. BMC Public Health. 2012 Nov 23;12(1):1023.

24. Mäkelä P, Osterberg E. Weakening of one more alcohol control pillar: a review of the effects of the alcohol tax cuts in Finland in 2004. Addict Abingdon Engl. 2009 Apr;104(4):554–63.

25. McCambridge J, Hawkins B, Holden C. Vested Interests in Addiction Research and Policy. The challenge corporate lobbying poses to reducing society's alcohol problems: insights from UK evidence on minimum unit pricing. Addiction. 2014 Feb 1;109(2):199–205.

26. Meng Y, Brennan A, Purshouse R, Hill-McManus D, Angus C, Holmes J, et al. Estimation of own and cross price elasticities of alcohol demand in the UK--A pseudo-panel approach using the Living Costs and Food Survey 2001-2009. J Health Econ. 2014 Mar;34:96–103.

For peer review only - http://bmjopen.bmj.com/site/about/guidelines.xhtml

27. Nelson JP. Does Heavy Drinking by Adults Respond to Higher Alcohol Prices and Taxes? A Survey and Assessment. Econ Anal Policy. 2013 Dec;43(3):265–91.

28. Nelson JP. Alcohol Affordability and Alcohol Demand: Cross-Country Trends and Panel Data Estimates, 1975 to 2008. Alcohol Clin Exp Res. 2014 Apr 1;38(4):1167–75.

29. Nelson JP. Estimating the Price Elasticity of Beer: Meta-Analysis of Data with Heterogeneity, Dependence, and Publication Bias [Internet]. Rochester, NY: Social Science Research Network; 2013 Jan [cited 2016 Feb 27]. Report No.: ID 2200492. Available from: http://papers.ssrn.com/abstract=2200492

30. Nelson JP. Gender differences in alcohol demand: a systematic review of the role of prices and taxes. Health Econ. 2014 Oct;23(10):1260–80.

31. Nicholls J, Greenaway J. What is the problem?: Evidence, politics and alcohol policy in England and Wales, 2010–2014. Drugs Educ Prev Policy. 2015 Mar 4;22(2):135–42.

32. Record C, Day C. Britain's alcohol market: how minimum alcohol prices could stop moderate drinkers subsidising those drinking at hazardous and harmful levels. Clin Med. 2009 Oct 1;9(5):421–5.

33. Rush B, Steinberg M, Brook R. The relationships among alcohol availability, alcohol consumption and alcohol-related damage in the Province of Ontario and the State of Michigan 1955-1982. Adv Alcohol Subst Abuse. 1986;5(4):33–45.

34. Shi Y. Three Essays on Economics of Health Behavior in China [Internet]. 2011 [cited 2016 Mar 20]. Available from: http://www.rand.org/pubs/rgs_dissertations/RGSD287.html

35. Skov SJ, Chikritzhs TN, Kypri K, Miller PG, Hall WD, Daube MM, et al. Is the "alcopops" tax working? Probably yes but there is a bigger picture. Med J Aust [Internet]. 2011 [cited 2015 Nov 9];195(2). Available from: https://www.mja.com.au/journal/2011/195/2/alcopops-tax-working-probably-yes-there-bigger-picture

36. Stockwell T, Williams N, Pauly B. Working and waiting: Homeless drinkers responses to less affordable alcohol. Drug Alcohol Rev. 2012 Sep 1;31(6):823–4.

37. Stockwell T, Zhao J, Martin G, Macdonald S, Vallance K, Treno A, et al. Misleading UK alcohol industry criticism of Canadian research on minimum pricing. Addiction. 2013 Jun 1;108(6):1172–3.

38. Tian G, Liu F. Is the demand for alcoholic beverages in developing countries sensitive to price? Evidence from China. Int J Environ Res Public Health. 2011 Jun;8(6):2124–31.

39. Xuan Z, Blanchette J, Nelson TF, Heeren T, Oussayef N, Naimi TS. The alcohol policy environment and policy subgroups as predictors of binge drinking measures among US adults. Am J Public Health. 2015 Apr;105(4):816–22.

40. Yashkin A. The Dynamics of Alcohol Consumption in the Russian Federation: Implications of Using Price Related Policies to Control Alcohol Use. Grad Theses Diss [Internet]. 2013 Jan 1; Available from: http://scholarcommons.usf.edu/etd/4968

For peer review only - http://bmjopen.bmj.com/site/about/guidelines.xhtml

41. Ally AK, Meng Y, Chakraborty R, Dobson PW, Seaton JS, Holmes J, et al. Alcohol tax pass-through across the product and price range: do retailers treat cheap alcohol differently? Addiction. 2014 Dec 1;109(12):1994–2002.

42. Anderson P, Chisholm D, Fuhr DC. Effectiveness and cost-effectiveness of policies and programmes to reduce the harm caused by alcohol. Lancet Lond Engl. 2009 Jun 27;373(9682):2234–46.

43. Centre for Economics and Business Research. Minimum Alcohol Pricing: A targeted measure? Report to the Scottish Parliamentary Health and Sport Committee [Internet]. London; 2010 Aug. Available from: http://www.ias.org.uk/uploads/pdf/Price%20docs/Updated-Sheffield-Scotland-v2-August-20103.pdf

44. Craven BM, Marlow ML, Shiers AF. The Economics of Minimum Pricing for Alcohol. Econ Aff. 2013 Jun 1;33(2):174–89.

45. Duffy J, Snowdon C. The minimal evidence for minimum pricing [Internet]. Adam Smith Institute; Available from: http://www.adamsmith.org/sites/default/files/research/files/ASI_SAPM.pdf

46. Fitzgerald N, Angus C. Four Nations: How Evidence-based are Alcohol Policies and Programmes across the UK?. London: [Internet]. London: Alliance for Useful Evidence/Alcohol Health Alliance.; 2015. Available from: http://www.alliance4usefulevidence.org/assets/Four-Nations-v3.pdf

47. Gallet CA. The Demand for Alcohol: A Meta-Analysis of Elasticities [Internet]. Rochester, NY: Social Science Research Network; 2007 May [cited 2016 Jan 6]. Report No.: ID 985689. Available from: http://papers.ssrn.com/abstract=985689

48. Gray D, Saggers S, Atkinson D, Sputore B, Bourbon D. Beating the grog: an evaluation of the Tennant Creek liquor licensing restrictions. Aust N Z J Public Health. 2000 Feb;24(1):39–44.

49. Gruenewald PJ, Ponicki WR, Holder HD, Romelsjö A. Alcohol prices, beverage quality, and the demand for alcohol: quality substitutions and price elasticities. Alcohol Clin Exp Res. 2006 Jan;30(1):96–105.

50. Hilton S, Wood K, Patterson C, Katikireddi SV. Implications for alcohol minimum unit pricing advocacy: What can we learn for public health from UK newsprint coverage of key claim-makers in the policy debate? Soc Sci Med. 2014 Feb;102:157–64.

51. Home Office. The likely impacts of increasing alcohol price: a summary review of the evidence base [Internet]. 2011 Jan. Available from: https://www.gov.uk/government/uploads/system/uploads/attachment_data/file/98100/impacts-alcohol-price-review.pdf

52. Institute for Fiscal Studies. Alcohol pricing and taxation policies. IFS Briefing Note BN124. 2011.

53. Institute for Fiscal Studies. Price-based measures to reduce alcohol consumption. IFS Briefing Note BN138. 2013.

For peer review only - http://bmjopen.bmj.com/site/about/guidelines.xhtml

54. Goodliffe J. Applying a minimum price to alcohol (Institute of Alcohol Studies) [Internet]. 2014. Available from: http://www.ias.org.uk/What-we-do/Publication-archive/Alcohol-Alert/October-2014/Applying-a-minimum-price-to-alcohol.aspx

55. Kisely S, Lawrence D. A time series analysis of alcohol-related presentations to emergency departments in Queensland following the increase in alcopops tax. J Epidemiol Community Health. 2015 Sep 16;jech – 2015–205666.

56. Ludbrook A. Purchasing Patterns for Low Price Off Sales Alcohol: Evidence from the Expenditure and Food Survey [Internet]. Available from: http://www.shaap.org.uk/images/UserFiles/File/Reports%20and%20Briefings/Briefing%20-%20Purchase%20of%20low-price%20alcohol%20analysis.pdf

57. Ornstein SI, Levy D. Price and Income Elasticities of Demand for Alcoholic Beverages. In: Galanter M, Begleiter H, Cicero T, Deitrich R, Goodwin DW, Gottheil E, et al., editors. Genetics Behavioral Treatment Social Mediators and Prevention Current Concepts in Diagnosis [Internet]. Boston, MA: Springer US; 1983. p. 303–45. Available from: http://dx.doi.org/10.1007/978-1-4613-3617-4_18

58. Rabinovich L, Brutscher P-B, de Vries H, Tiessen J, Clift J, Reding A. The affordability of alcoholic beverages in the European Union. 2012;

59. Radaev V. Impact of a New Alcohol Policy on Homemade Alcohol Consumption and Sales in Russia. Alcohol Alcohol. 2015 May 1;50(3):365–72.

60. Robinson M, McCartney G, Beeston C. What is convincing evidence on alcohol pricing? BMJ. 2013 Aug 20;347:f5102.

61. Snowdon C. Chapter 10. Minimum unit pricing. In: Flaws and Ceilings: Price controls and the damage they cause [Internet]. [cited 2015 Nov 9]. p. 177–97. Available from: http://www.iea.org.uk/publications/research/flaws-and-ceilings-price-controls-and-the-damage-they-cause

62. Wine and Spirits Trade Association. New claims that increasing the cost of alcohol saves lives are misleading [Internet]. Available from: http://www.wsta.co.uk/press/634-new-claims-that-increasing-the-cost-of-alcohol-saves-lives-are-misleading

63. World Health Organization. European status report on alcohol and health 2014. Pricing policies [Internet]. World Health Organization; 2014. Available from: http://www.euro.who.int/__data/assets/pdf_file/0003/244902/Pricing-policies.pdf

For peer review only - http://bmjopen.bmj.com/site/about/guidelines.xhtml

PRISMA 2009 Checklist

Section/topic	#	Checklist item	Reported on page #
**TITLE**			
Title	1	Identify the report as a systematic review, meta-analysis, or both.	1
**ABSTRACT**			
Structured summary	2	Provide a structured summary including, as applicable: background; objectives; data sources; study eligibility criteria, participants, and interventions; study appraisal and synthesis methods; results; limitations; conclusions and implications of key findings; systematic review registration number.	2
**INTRODUCTION**			
Rationale	3	Describe the rationale for the review in the context of what is already known.	3
Objectives	4	Provide an explicit statement of questions being addressed with reference to participants, interventions, comparisons, outcomes, and study design (PICOS).	3-4
**METHODS**			
Protocol and registration	5	Indicate if a review protocol exists, if and where it can be accessed (e.g., Web address), and, if available, provide registration information including registration number.	no published protocol
Eligibility criteria	6	Specify study characteristics (e.g., PICOS, length of follow-up) and report characteristics (e.g., years considered, language, publication status) used as criteria for eligibility, giving rationale.	3-4
Information sources	7	Describe all information sources (e.g., databases with dates of coverage, contact with study authors to identify additional studies) in the search and date last searched.	3
Search	8	Present full electronic search strategy for at least one database, including any limits used, such that it could be repeated.	3
Study selection	9	State the process for selecting studies (i.e., screening, eligibility, included in systematic review, and, if applicable, included in the meta-analysis).	3-4
Data collection process	10	Describe method of data extraction from reports (e.g., piloted forms, independently, in duplicate) and any processes for obtaining and confirming data from investigators.	4
Data items	11	List and define all variables for which data were sought (e.g., PICOS, funding sources) and any assumptions and simplifications made.	Tables 2+3
Risk of bias in individual studies	12	Describe methods used for assessing risk of bias of individual studies (including specification of whether this was done at the study or outcome level), and how this information is to be used in any data synthesis.	4
Summary measures	13	State the principal summary measures (e.g., risk ratio, difference in means).	Not stated, as all summary measures were included
Synthesis of results	14	Describe the methods of handling data and combining results of studies, if done, including measures of consistency (e.g., I^2) for each meta-analysis.	4, and Table 1

For peer review only - http://bmjopen.bmj.com/site/about/guidelines.xhtml

PRISMA 2009 Checklist

Section/topic	#	Checklist item	Reported on page #
Risk of bias across studies	15	Specify any assessment of risk of bias that may affect the cumulative evidence (e.g., publication bias, selective reporting within studies).	4
Additional analyses	16	Describe methods of additional analyses (e.g., sensitivity or subgroup analyses, meta-regression), if done, indicating which were pre-specified.	4
**RESULTS**			
Study selection	17	Give numbers of studies screened, assessed for eligibility, and included in the review, with reasons for exclusions at each stage, ideally with a flow diagram.	Figure 1 and online supplementary file
Study characteristics	18	For each study, present characteristics for which data were extracted (e.g., study size, PICOS, follow-up period) and provide the citations.	Tables 2+3
Risk of bias within studies	19	Present data on risk of bias of each study and, if available, any outcome level assessment (see item 12).	Tables 2+3
Results of individual studies	20	For all outcomes considered (benefits or harms), present, for each study: (a) simple summary data for each intervention group (b) effect estimates and confidence intervals, ideally with a forest plot.	Tables 2+3, p18-20
Synthesis of results	21	Present results of each meta-analysis done, including confidence intervals and measures of consistency.	No meta-analysis, discussed p18-20
Risk of bias across studies	22	Present results of any assessment of risk of bias across studies (see Item 15).	discussed p21
Additional analysis	23	Give results of additional analyses, if done (e.g., sensitivity or subgroup analyses, meta-regression [see Item 16]).	18-20
**DISCUSSION**			
Summary of evidence	24	Summarize the main findings including the strength of evidence for each main outcome; consider their relevance to key groups (e.g., healthcare providers, users, and policy makers).	20-21
Limitations	25	Discuss limitations at study and outcome level (e.g., risk of bias), and at review-level (e.g., incomplete retrieval of identified research, reporting bias).	20-21
Conclusions	26	Provide a general interpretation of the results in the context of other evidence, and implications for future research.	20
**FUNDING**			
Funding	27	Describe sources of funding for the systematic review and other support (e.g., supply of data); role of funders for the systematic review.	22

From: Moher D, Liberati A, Tetzlaff J, Altman DG, The PRISMA Group (2009). Preferred Reporting Items for Systematic Reviews and Meta-Analyses: The PRISMA Statement. PLoS Med 6(7): e1000097. doi:10.1371/journal.pmed1000097

For more information, visit: **www.prisma-statement.org**.

For peer review only - http://bmjopen.bmj.com/site/about/guidelines.xhtml

BMJ Open

Evidence for the effectiveness of minimum pricing of alcohol: a systematic review and assessment using the Bradford Hill criteria for causality

Journal:	*BMJ Open*
Manuscript ID	bmjopen-2016-013497.R1
Article Type:	Research
Date Submitted by the Author:	21-Nov-2016
Complete List of Authors:	Boniface, Sadie; King's College London, National Addiction Centre, Institute of Psychiatry, Psychology and Neuroscience<br>Scannell, Jack; University of Edinburgh, Innogen Institute, School of Social and Political Sciences<br>Marlow, Sally; King's College London, National Addiction Centre, Institute of Psychiatry, Psychology and Neuroscience
**Primary Subject Heading**:	Public health
Secondary Subject Heading:	Addiction
Keywords:	alcohol, policy, minimum unit pricing, PUBLIC HEALTH, Bradford Hill

SCHOLARONE™
Manuscripts

For peer review only

Evidence for the effectiveness of minimum pricing of alcohol: a systematic review and assessment using the Bradford Hill criteria for causality

Sadie Boniface,[1] Jack W Scannell,[2] Sally Marlow[1]

[1]National Addiction Centre, Institute of Psychiatry, Psychology and Neuroscience, King's College London, London, UK

[2]School of Social and Political Science, University of Edinburgh, Edinburgh, UK

Corresponding author: Dr Sadie Boniface, National Addiction Centre, Addiction Sciences Building (PO 48) Institute of Psychiatry, Psychology and Neuroscience, 16 De Crespigny Park, King's College London, SE5 8AF. sadie.boniface@kcl.ac.uk 020 7848 5097

Keywords: alcohol, policy, minimum unit pricing, public health, Bradford Hill

Word count: 3,224 (excluding title page, abstract, references, figures and tables)

For peer review only

ABSTRACT

Objectives: To assess the evidence for price-based alcohol interventions to determine whether minimum unit pricing policies are likely to be effective.

Design: Systematic review and assessment of studies according to PRISMA guidelines, against the Bradford Hill criteria for causality. Three electronic databases were searched from inception to February 2016. Additional articles were found through hand searching and grey literature searches.

Criteria for selecting studies: We included any study design that reported on the effect of price changes and price-based interventions on alcohol consumption or alcohol-related morbidity, mortality, and wider harms. Studies reporting on the effects of taxation or affordability, and studies that only investigated price elasticity of demand were beyond the scope of this review. Studies with any conflict of interest were excluded. All studies were appraised for methodological quality.

Results: Of 756 studies assessed, 30 studies were included: 23 peer-reviewed research studies and 7 from the grey literature. All nine of the Bradford Hill criteria were met, although different types of study satisfied different criteria. For example, modelling studies complied with the consistency and specificity criteria, time series analyses demonstrated the temporality and experiment criteria, and the analogy criterion was fulfilled by comparing the findings with the wider literature on taxation and affordability.

Conclusions: Overall, the Bradford-Hill criteria for causality were satisfied. There was very little evidence that alcohol price was not associated with consumption or subsequent harms. However the overall quality of the evidence was variable, a large proportion of the evidence base has been produced by a small number of research teams, and the quantitative uncertainty in many estimates or forecasts is poorly communicated in the literature. None the less, price-based alcohol policy interventions such as minimum unit pricing are likely to reduce alcohol consumption, alcohol-related morbidity and mortality.

ARTICLE SUMMARY

Strengths and limitations of this study

- This review adds to an emerging literature of systematic reviews synthesising findings using the Bradford Hill criteria for causality in research areas where traditional meta-analyses of randomised controlled trials are not possible or appropriate
- A range of study designs were included, allowing for a comprehensive review of a disparate evidence base to investigate whether minimum unit pricing of alcohol is likely to reduce alcohol consumption and alcohol-related harm
- Studies examining the effects of alcohol taxation or changes in alcohol affordability, or studies solely reporting on price elasticity of demand, were not included
- Methodological quality of studies was variable

For peer review only

INTRODUCTION

Alcohol-related harm costs the NHS £3.5bn each year and the estimated cost to society is £21 billion per year (1). The latest annual figures for England (population of 54 million) show over one million alcohol-related hospital admissions (2013/14) and six and a half thousand alcohol-related deaths (2013); and these figures represent increases compared with a decade previously of 115% and 10% respectively (1). There are many policies and programmes that aim to reduce harms from alcohol (2), and one of these is minimum pricing. Minimum pricing for alcohol has been introduced in a number of countries around the world including Canada (3), Belarus, Kyrgyzstan, the Republic of Moldova, the Russian Federation and Ukraine (4). In 2012 the UK coalition Government cited support for minimum unit pricing (MUP) in its alcohol strategy (5), and legislation to have a minimum price of £0.50 per unit (one UK unit = 10ml or 8g ethanol) was passed in Scotland the same year (4). In England and Wales there has been a ban on alcohol being sold at below cost (the total amount of 'duty plus VAT') since May 2014 (6); and the first conviction for selling alcohol below this level recently took place (7). Duty plus VAT is equivalent to a 70cl bottle of vodka (37.5% ABV) costing a minimum of £8.72 (8), whereas under a minimum price of 50 pence per unit this would cost £13.13.

Following the change from a Conservative and Liberal Democrat coalition Government to a Conservative majority Government in 2015, it is unclear whether there is still central Government support for MUP. There have been discussions in the Scottish courts between health organisations and the alcohol industry around the legality of MUP, proportionality (that the same objective cannot be met through increased taxation), and whether there is sufficient evidence. In a recent report about the extent to which UK alcohol policies are evidence-based, Fitzgerald and Angus wrote that "there are also a number of notable instances of policies being rejected due to 'insufficient evidence' with little indication of what level of evidence would be considered to be 'sufficient'" (9).

Taxation and price interventions are sometimes considered analogous, however it is at the retailers' discretion whether or not to pass on tax increases to consumers, but this is not the case for MUP. In this paper, we assess the effect of price and price-based interventions as MUP is currently being considered as a policy option in the United Kingdom. We systematically review the literature on the effect of price changes or policies such as MUP on alcohol consumption, alcohol-related morbidity and mortality, and wider harms. We use the nine Bradford Hill criteria for causality as a framework with the aim of assessing the likely effectiveness of MUP as a policy to reduce alcohol consumption and alcohol-related harm.

METHODS

A systematic literature search was performed according to Preferred Reporting Items for Systematic Reviews and Meta-Analyses (PRISMA) guidance (see Figure 1 for PRISMA flow diagram and online supplementary file for excluded studies).

[figure 1 to go here]

Identification of studies

Three electronic databases were searched for titles or abstracts containing '"minimum unit pric$" OR "minimum pric$"' OR "floor pric$" OR "pric$ AND policy" AND alcohol. The databases were

For peer review only

PsycINFO (1806 to February Week 1 2016), Embase (1974 to 2016 Week 07), Ovid Medline (1946 to February Week 1 2016). We also searched the websites of five alcohol charities for publications or reports related to "price", and also searched 20 leading UK think tanks for "alcohol" or "addiction".

Inclusion criteria were: any study design; population level studies exploring at least one aspect of the effect of changes in the minimum price of alcohol, including but not limited to changes in alcohol sales, consumption, morbidity and mortality; individual level studies exploring differences in price paid for alcohol, and alcohol purchasing, consumption, morbidity and mortality; written in English.

Exclusion criteria were: studies about taxation, affordability and price elasticity of demand for alcohol (there is a large literature on each of these already and reviewing all of these studies was beyond the scope of this review); studies about public perceptions of MUP; studies where a conflict of interest was reported in the paper, whether this was in favour of or against MUP.

All 32 studies that met the inclusion criteria were assessed against the Bradford Hill criteria for causality and the methodological quality appraised. These included 23 original research studies, 2 systematic reviews, and 7 studies from the grey literature. Of the 23 research studies, there were 9 cross sectional surveys, 8 time series analyses or similar, 4 econometric modelling studies, one qualitative study, and one trial.

Analysis of included studies

Quality of included studies was assessed independently by two reviewers and using validated tools. Due to the wide variation in study designs among the included studies, the Effective Public Health Practice Project's (EPHPP) tool was used for assessing all quantitative studies, as recommended by the Cochrane Handbook for assessing studies in public health (10). Qualitative studies (n=1) and systematic reviews (n=2) included in this review were not covered by the EPHPP tool and so were assessed using the Critical Appraisal Skills Programme (CASP) tools specific to these study designs.

Nine criteria in order to determine causality were suggested by Bradford Hill in an influential 1965 paper (11). Increasingly, the Bradford Hill criteria are a standard framework to assess the impact of interventions where it is not ethical or practical to conduct randomised controlled trials. Our interpretation of the Bradford Hill criteria for the purpose of this review is listed in Table 1. Two reviewers assessed each study against each of the nine criteria and agreed which studies provided relevant evidence for or against each criterion.

For peer review only

Table 1: Bradford Hill criteria for assessing causation and the definitions used in this review

Criterion	Bradford Hill criteria (1965)	Application in this review
1. Strength of the association	The strength of a supposed association between an intervention and an outcome is determined by the appropriate statistic used to measure the protective effect of an intervention (e.g. relative risk or odds ratio). This is the most important factor determining causation	A statistically significant change (P<0.05) in alcohol consumption or alcohol related harms, in the expected direction. The exact magnitude of the association was assessed on a study by study basis
2. Consistency	Has it been repeatedly observed by different persons, in different places, circumstances and times?	Whether different studies conducted in different locations, in different populations, by different investigators and at different times have reported similar findings
3. Specificity	Specificity is present when the intervention is exclusive to the outcome and when the outcome has no other known cause or associated risk factors; cautions that this criterion should not be overemphasized and that if specificity is not apparent this does not preclude causation	If pricing was the only reason that alcohol consumption or alcohol-related harm could have fallen, this adds to the argument for causality. However if a price intervention was one of a number of alcohol policy interventions, then this criterion is not satisfied
4. Temporality	Refers to temporal relationship of association between exposure and disease outcome; to infer causality, exposure must precede outcome	The pricing intervention studied must have taken place before a change in alcohol consumption or harm was observed
5. Dose-response	If the association is one in which a dose-response curve or biological gradient can be observed, this adds to the case for causality	If interventions leading to a larger increase in prices had a greater effect on alcohol consumption and alcohol related harm than interventions where the price change was small, or if studies demonstrate that different prices have differing effects, in the expected direction
6. Plausibility	A likely biological mechanism linking the intervention to the observed findings helps to explain causality, plausibility depends on biological knowledge of the day	Studies that found an association between price and population-level alcohol consumption and that heavier drinkers tend to purchase the cheapest alcohol could demonstrate plausibility
7. Coherence	When the evidence from different disciplines sources “hangs well together” and does not conflict with other generally known facts, this criterion is met	Describes whether studies conducted in different settings or disciplines had complementary findings. Will not be demonstrated by a single study in isolation but rather the evidence base as a whole
8. Experiment	Experimental evidence from laboratory studies or RCTs could potentially provide strongest support for causation	In addition to laboratory studies and RCTs, natural experiments with before-and-after measures could also show the effectiveness

For peer review only

	This criterion often provides the strongest support for causation and describes whether there is empirical evidence for the association	of minimum unit pricing in a 'real world' setting
9. Analogy	Causality is supported by analogy if there are similar associations or causal relationships in other areas of relevance, weakest form of evidence of causality	Other areas of relevance include whether higher taxation on alcohol is associated with reduced alcohol consumption and alcohol related harm, and may require drawing on additional literature outside of the main systematic review

For peer review only

RESULTS

The included studies that are published in peer-reviewed journals (23 research studies and two systematic reviews) are listed by study type in Table 2 with information on study characteristics and methodological quality. Of the research studies, the methodological quality was rated as 'strong' in 12 studies, 'moderate' in 8 studies, and 'weak' in 3 studies. Both of the systematic reviews were rated 'strong'. The seven reports from the grey literature are listed in Table 3. Five of the seven were rated as of 'strong' methodological quality, with the remaining two not appropriate to rate using our critical appraisal tool.

For peer review only

Table 2: Studies published in peer-reviewed journals included in Bradford Hill criteria assessment

Study type	First author and year published	Study characteristics					Study assessment			Bradford Hill criteria met*
		Country	Study design	Population or participants	Pricing intervention studied	Outcomes studied	Peer-reviewe d	Conflict of interes t	Quality rating	
Natural experiment s and time series analyses	Bhattachary a 2013 (12)	Russia	Time series analysis of panel data set	Populations of 77 Russian oblasts (provinces), 1970-2000	Substantial increases in administratively -set alcohol prices 1985-1988, along with 6 other anti-alcohol measures	Mortality	Yes	Not stated	Strong	SA, CON, TE, PL, CO, EX,
	Herttua 2015 (13)	Finland	Time series analysis	General population using population registry	Modelled 1% increase in the average minimum price of all alcoholic beverages based on actual price increases adjusted for inflation using Consumer Price Index	Alcohol related mortality	Yes	None	Strong	SA (not universal findings – subgrou p only), CON (counter findings) TE, PL, CO, EX
	Stockwell 2012 (3)	Canada	Cross-section versus time series analysis of	Population of British Columbia	Actual minimum price increased over a 20 year period. Study modelled a 10%	Alcohol consumption (measured by sales)	Yes	None	Strong	SA, CON, TE, DR, CO, EX

For peer review only - http://bmjopen.bmj.com/site/about/guidelines.xhtml

Study type	First author and year published	Study characteristics					Study assessment			Bradford Hill criteria met*
		Country	Study design	Population or participants	Pricing intervention studied	Outcomes studied	Peer-reviewe d	Conflict of interes t	Quality rating	
			ecological data		increase in the average minimum price of all alcoholic beverages adjusted by monthly Consumer Price Index adjusted by monthly Consumer Price Index					
	Stockwell 2012 (14)	Canada	Cross-section versus time series analysis of ecological data	Population of Saskatchewan	Actual minimum price increased over a 7 year period. Study modelled a 10% increase in the average minimum price of all alcoholic beverages adjusted by monthly Consumer Price Index adjusted by monthly Consumer Price	Alcohol consumption (measured by sales)	Yes	Not stated	Strong	SA, CON, TE, DR, CO, EX

For peer review only - http://bmjopen.bmj.com/site/about/guidelines.xhtml

Study type	First author and year published	Study characteristics					Study assessment			Bradford Hill criteria met*
		Country	Study design	Population or participants	Pricing intervention studied	Outcomes studied	Peer-reviewed	Conflict of interest	Quality rating	
					Index					
	Stockwell 2013 (15)	Canada	Cross-section versus time series analysis of ecological data	Populations of 89 geographic areas in British Columbia	Actual minimum price increased over a 20 year period. Study modelled 10% increase in the average minimum price of all alcoholic beverages adjusted by monthly Consumer Price Index	Alcohol-attributable hospital admissions	Yes	Not stated	Strong	SA, CON, TE, DR, PL, CO, EX
	Treisman 2010 (16)	Russia	Secondary analysis of historical data with focus on price changes 1990-1994	Population of Russia	Price liberalisation of vodka in early 1990s - in 1993 real price of vodka was around 25% of that in 1990	Mortality	Yes	Not stated	Strong	SA, CON, TE, PL, CO, EX
	Wald 1984 (17)	Poland	Analysis of routine data 1970-1981	Population of Poland	Poor harvest led to high prices, rationing and illegal sales	Alcohol consumption and alcohol-related hospital	Yes	Not stated	Weak	CON, TE, PL, CO, EX

For peer review only - http://bmjopen.bmj.com/site/about/guidelines.xhtml

Study type	First author and year published	Study characteristics					Study assessment			Bradford Hill criteria met*
		Country	Study design	Population or participants	Pricing intervention studied	Outcomes studied	Peer-reviewed	Conflict of interest	Quality rating	
						admissions				
	Zhao 2013 (18)	Canada	Cross-section versus time series analysis of ecological data	Populations of 16 Health Service Delivery Areas in British Columbia, Canada	Actual minimum price increased over a 20 year period. Study modelled 10% increase in the average minimum price of all alcoholic beverages adjusted by monthly Consumer Price Index. Also looked at outlet density	Acute, chronic and wholly alcohol attributable mortality	Yes	None	Strong	SA, CON, TE, DR, PL, CO, EX
Modelling studies	Brennan 2014 (19)	England	Modelling study using SAPM	UK national surveys of general population (subgroups of moderate, harmful, hazardous)	MUP of £0.40, £0.45 and £0.50. Ban on below cost selling	Alcohol consumption, consumer spending, 47 health harms, QALYs	Yes	None	Strong	CON, SP, DR, PL, CO
	Holmes 2014 (20)	England	Modelling study using SAPM	UK national surveys of general	MUP of 45p	Alcohol consumption, consumer	Yes	None	Strong	CON, SP, PL, CO

For peer review only - http://bmjopen.bmj.com/site/about/guidelines.xhtml

Study type	First author and year published	Study characteristics					Study assessment			Bradford Hill criteria met*
		Country	Study design	Population or participants	Pricing intervention studied	Outcomes studied	Peer-reviewed	Conflict of interest	Quality rating	
				population (subgroups of moderate, harmful, hazardous)		spending, 47 health harms, QALYs				
	Meier 2009 (21)	UK	Modelling study using SAPM	UK national surveys of general population (subgroups of moderate, harmful, hazardous)	10 pricing policy options, including different levels of MUP (of 33 analysed)	Alcohol consumption, consumer spending, 47 health harms, crime, employment	Yes	None	Strong	CON, SP, DR, PL, CO
	Purshouse 2010 (22)	England	Modelling study using SAPM	UK national surveys of general population (subgroups of moderate, harmful, hazardous)	18 different pricing policies	Alcohol consumption, consumer spending, 47 health harms, QALYs	Yes	None	Strong	CON, SP, DR, PL, CO
Cross-sectional studies	Black 2011(23)	Scotland	Cross-sectional survey	377 hospital patients with serious alcohol problems	Mean price paid per unit	Alcohol consumption	Yes	None	Moderate	SA CON, DR, PL, CO
	Callinan 2015 (24)	Australia	Cross-sectional survey	Drinkers 18+ participating in Australian	Price paid for alcohol	Alcohol consumption	Yes	Not stated	Moderate	SA, CON, DR, PL, CO

For peer review only - http://bmjopen.bmj.com/site/about/guidelines.xhtml

Study type	First author and year published	Study characteristics					Study assessment			Bradford Hill criteria met*
		Country	Study design	Population or participants	Pricing intervention studied	Outcomes studied	Peer-reviewed	Conflict of interest	Quality rating	
				International Alcohol Control study (n=1,681)						
	Casswell 2014 (25)	New Zealand	Cross-sectional survey	Drinkers 18+ participating in NZ International Alcohol Control study (n=1,900)	Price paid per drink in on and off trade	Alcohol consumption	Yes	Not stated	Strong	SA, CON, PL, CO
	Crawford 2012 (26)	England	Cross-sectional survey	515 members of the public	Median price paid per unit	AUDIT score	Yes	None	Moderate	SA, CON, PL, CO
	Falkner 2015 (27)	New Zealand	Cross-sectional survey	115 adults undergoing alcohol detoxification	Price paid for alcohol	Alcohol consumption	Yes	No	Moderate	SA, CON, PL, CO
	Forsyth 2014 (28)	Scotland	Cross-sectional survey	Shopkeepers of 144 off licences in Glasgow	MUP of £0.50	Products affected, and hospital admissions	Yes	None	Weak	CON, PL (weakly), CO
	Ludbrook 2012 (29)	UK	Cross-sectional survey	Expenditure and Food Survey data from 2006-8 (n=18,624)	Purchasers of alcohol less than £0.45 per unit	Income of purchasers of cheap alcohol	Yes	Not stated	Moderate	SA, CON, PL, CO

For peer review only - http://bmjopen.bmj.com/site/about/guidelines.xhtml

Study type	First author and year published	Study characteristics					Study assessment			Bradford Hill criteria met*
		Country	Study design	Population or participants	Pricing intervention studied	Outcomes studied	Peer-reviewed	Conflict of interest	Quality rating	
	Sharma 2014 (30)	Australia	Cross-sectional survey	Representative sample of households (n=885) completing shopping survey	MUP of A$1, and taxation	Alcohol consumption (measured by projected sales)	Yes	None	Moderate	SA, CON, DR, PL, CO
	Sheron 2014 (31)	UK	Cross-sectional survey	Adult patients in a liver unit of a hospital (n=204)	Median and mean price paid per unit	Alcohol consumption	Yes	Not stated	Moderate	SA, CON, DR, PL, CO
Intervention studies	Babor 1978 (32)	USA	Trial (not randomised)	34 male volunteers in live-in research facility	'Happy Hour' with a reduction in price of alcohol for one group of participants	Alcohol consumption	Yes	Not stated	Weak	SA, CON, SP, TE, CO, EX
Qualitative studies	Seaman 2013 (33)	Scotland	Qualitative study	130 participants aged 16-30	Hypothetical price increases	Alcohol consumption and substitution with other substances	Yes	None	Moderate	CON, CO
Systematic reviews	Wagenaar 2009 (34)	Worldwide	Systematic review and meta-analysis	Studies tended to cover general population	Alcohol price and taxation interventions studied together	Alcohol consumption (measured by alcohol sales or self-	Yes	None	Strong	AN

For peer review only - http://bmjopen.bmj.com/site/about/guidelines.xhtml

Study type	First author and year published	Study characteristics					Study assessment			Bradford Hill criteria met*
		Country	Study design	Population or participants	Pricing intervention studied	Outcomes studied	Peer-reviewed	Conflict of interest	Quality rating	
						reported consumption)				
	Wagenaar 2010 (35)	Worldwide	Systematic review and meta-analysis	Studies tended to cover general population	Alcohol price and taxation interventions studied together	Alcohol-related morbidity (disease, injury, suicide, traffic crashes, sexually transmitted diseases, other drug use, crime and misbehaviour) and mortality	Yes	Not stated	Strong	AN

*Abbreviations used for the Bradford Hill criteria: strength of the association (SA), consistency (CON), specificity (SP), temporality (TE), dose-response (DR), plausibility (PL), coherence (CO), experiment (EX), analogy (AN)

For peer review only - http://bmjopen.bmj.com/site/about/guidelines.xhtml

Table 3: Studies published in the grey literature included in Bradford Hill criteria assessment

	Study characteristics					Study assessment			Bradford Hill criteria met*
**Author and year published**	**Country**	**Study design**	**Population or participants**	**Pricing intervention studied**	**Outcomes studied**	**Peer-reviewed?**	**Conflict of interest?**	**Quality rating**	
Angus 2016 (36)	Scotland	Modelling study using SAPM	Scottish general population survey (subgroups of moderate, harmful, hazardous)	MUP of 30p, 40p, 50p, 60p and 70p, compared with taxation interventions	Alcohol consumption, consumer spending, exchequer and retail revenue, 47 health harms	Not stated	None	Strong	CON, SP, DR, PL, CO
Booth 2008 (37)	Worldwide	Review of reviews and systematic review	Studies tended to cover general population	Various minimum unit prices and taxation interventions	Alcohol consumption and various measures of alcohol harm	Yes	None	Strong	AN
Brennan 2008 (38)	England	Modelling study using SAPM	Adults in England	General price increases. MUP of £0.20, £0.25, £0.30, £0.35, £0.40, £0.45, £0.50, £0.60 and £0.70. Restrictions on off-trade price promotions.	Alcohol consumption, consumer spending, sales duty and VAT, 47 health harms, crime, and employment	Not stated	None	Strong	CON, SP, DR, PL, CO
Hill McManus 2012 (39)	Canada	Modelling study using SAPM	Adults in two Canadian provinces (Ontario and British Columbia)	MUP of C$1.50	Alcohol consumption, consumer spending, hospital admissions, mortality, crime	No	None	Strong	CON, SP, PL, CO
Institute for Fiscal Studies	Great Britain	Economic modelling study	Shopping data from 25,248 British households	MUP of £0.45	Alcohol consumption	Not stated	Not stated	Not possible to rate	CON, SP, CO

For peer review only - http://bmjopen.bmj.com/site/about/guidelines.xhtml

	Study characteristics					Study assessment			Bradford Hill criteria met*
**Author and year published**	**Country**	**Study design**	**Population or participants**	**Pricing intervention studied**	**Outcomes studied**	**Peer-reviewed?**	**Conflict of interest?**	**Quality rating**	
2010 (40)		using market research data							
Institute for Fiscal Studies 2013 (41)	Great Britain	Economic analysis	Population of Great Britain	MUP of £0.45 and increased alcohol taxation	Alcohol consumption	Not stated	Not stated	Not possible to rate	CON, SP, CO
Meng 2010 (42)	Scotland	Modelling study using SAPM	Adults in Scotland	MUP of £0.20, £0.25, £0.30, £0.35, £0.40, £0.45, £0.50, £0.60 and £0.70. Restrictions on off-trade price promotions.	Alcohol consumption, consumer spending, 47 health harms, crime, employment	Not stated	None	Strong	CON, SP, DR, PL, CO

*Abbreviations used for the Bradford Hill criteria: strength of the association (SA), consistency (CON), specificity (SP), temporality (TE), dose-response (DR), plausibility (PL), coherence (CO), experiment (EX), analogy (AN)

For peer review only - http://bmjopen.bmj.com/site/about/guidelines.xhtml

Consideration against the Bradford Hill criteria for determining causality

1. Strength of the association

In 16/23 studies published in peer-reviewed journals, strength of the association between pricing and alcohol consumption or alcohol related harm was evidenced by a summary statistic such as an odds ratio, and by a test of the statistical significance of the association. As well as the statistical significance of the summary statistic, the magnitude of the effect was also considered, as a larger effect size corresponds to a greater population health impact. Studies in Canada found that 10% increases in minimum prices were associated with reductions in alcohol consumption of 3.4-8.4% (3,14), reductions in alcohol attributable hospital admissions of 9% (15), and reductions in alcohol-related mortality of 32%, each of which was statistically significant (18). Cross-sectional studies in the UK, Australia and New Zealand (23–27,29–31) and one trial from the USA (32) found statistically significant associations between cheaper alcohol and heavier drinking. The magnitude of the association varied across these studies but due to different study measures and outcomes the results are not all comparable. As an indication the odds ratio (OR) for buying cheap alcohol among heavier drinkers was 1.34 in Crawford's study (26) and 1.70 in Callinan's study (24), and in Casswell's study the odds of being a heavier drinker were roughly double among cheap alcohol purchasers (25). There was not any evidence to support this criterion from the grey literature. Overall there is reasonably good support for the strength of the association criterion.

2. Consistency

This criterion requires looking across all the studies included in the review to see whether similar conclusions have been drawn. Inverse associations between alcohol pricing and alcohol consumption or harm have been documented in countries in Europe, North America and Australia, and although most studies are from the last 10 years, there are studies from the 1970s and 1980s as well. There is evidence from different research teams and different types of study including cross-sectional studies, time series analyses and econometric modelling studies. Support for the consistency criterion is very strong.

3. Specificity

The specificity criterion relates to whether changes in alcohol consumption or harm could be attributed to anything other than the price intervention. Many studies included have statistically adjusted for confounding factors, however the best support for the specificity criterion comes from the econometric modelling studies because there is no risk of residual confounding. The Sheffield Alcohol Policy Model is one such model and has been applied in England (19–22,38), Scotland (36,42) and Canada (39) and provides very strong support for the specificity criterion. Further support is provided by other different modelling studies in the UK (40,41) and a trial in the US (32). Thus support for the specificity criteria is very strong.

4. Temporality

It is important that price changes or price interventions take place before changes to alcohol consumption and harm in order to attribute causality. Strong support for this criterion comes from research following the introduction of MUP in Canada, where price increases preceded reductions in alcohol consumption (3,14), alcohol attributable hospital admissions (15), and alcohol-related

For peer review only - http://bmjopen.bmj.com/site/about/guidelines.xhtml

mortality (18). Studies where price changes preceded the expected changes in alcohol consumption or harm have also been conducted in Russia (12,16), Poland (17), and Finland (13). Overall there is very strong support for the temporality criterion.

5. Dose-response/biological gradient

This criterion is supported if different price levels have been found to have differing effects on consumption or harm. Many of the studies using the Sheffield Alcohol Policy Model explore the impact of a range of potential MUP options (19,21,22,36,42), and these consistently suggest that the higher the MUP, the greater the reductions in alcohol consumption or alcohol-related harms. The Canadian studies of minimum pricing lend further support for this criterion because the analysis presents the effect on consumption or harm of a modelled 1% increase in price, meaning dose-response can be inferred (3,14,15,18). Dose response is supported to a lesser extent by evidence from cross-sectional studies that heavier drinkers pay less for their alcohol (23,24,30,31). Overall, there is strong support for the dose-response criterion, although the relationship is difficult to quantify.

6. Plausibility

This criterion refers to whether there is evidence that alcohol price can be used as an economic mechanism to influence consumption at a population level, and whether heavy drinkers tend to purchase cheaper alcohol. There is evidence from 11/28 research studies and 4/7 studies in the grey literature that the price of alcohol is inversely related to alcohol-related morbidity, hospital admissions, or mortality. Moreover, there is also evidence from numerous cross-sectional studies in the UK, Australia and New Zealand (23–27,29–31) and one trial from the USA (32) that heavier drinking was significantly associated with cheaper alcohol, suggesting that economic mechanisms such as minimum pricing would particularly affect the heaviest drinkers. This provides strong support for the plausibility criterion.

7. Coherence

This criterion refers to whether studies from different disciplines have had complementary findings and whether these fit or 'hang' well together. It is different to consistency, which is more concerned with reproducibility of findings. The findings of the majority of studies supported the coherence criterion in that they suggest that real-world minimum unit pricing (e.g. (3,14,15,18)) or price increases (e.g. (12,13,17)) led to reductions in alcohol consumption and alcohol-related harm, modelling studies suggest heavier drinkers will be most affected by MUP (e.g. (20)), and cross sectional surveys find that it is the heavier drinkers that are drinking the cheapest alcohol (e.g. (23,31)). Overall the evidence base provides strong support for this criterion.

8. Experiment

We have not identified any randomised controlled trials of minimum pricing or price-based interventions to reduce alcohol consumption. There is a small (and not randomised) trial from the 1970s (32) which found participants living in controlled conditions and offered a daily 'Happy Hour' discount drank significantly more alcohol than those who were not offered the discount. There is however substantial evidence in support of the experiment criterion from time series analyses or natural experiments, for example: where minimum pricing was introduced in Canada (3,14,15,18)

For peer review only - http://bmjopen.bmj.com/site/about/guidelines.xhtml

and where prices fluctuated in the late 1980s and early 1990s in Russia (12,16), and to a lesser extent in Finland, where price increases were associated with reduced mortality only among men with a basic education (13). These studies provide tentative support for the experiment criterion.

9. Analogy

To address the analogy criterion areas related to alcohol minimum pricing must be considered. There is evidence from literature on the affordability of alcohol (43) that consumption and harm are very responsive to the affordability of alcohol. Large systematic reviews have investigated the price elasticity of demand for alcohol (44), and have found that higher alcohol pricing and taxation (considered together) are associated with reductions in alcohol consumption, alcohol-related morbidity and mortality (34,35,37). There are a number of arguments favouring minimum pricing as an intervention over increased taxation. One of these is that increases in taxation can sometimes be absorbed by retailers or only 'passed-through' to consumers of more expensive products, keeping cheap alcohol at a low price (45). Overall the support for the analogy criterion is very strong, although Bradford Hill describes this as the weakest evidence for causality.

DISCUSSION

We assessed 23 research studies and two systematic reviews, plus a further seven studies from the grey literature in this review of the evidence for priced based interventions – such as MUP - to reduce alcohol consumption and alcohol-related harm. All nine of the Bradford Hill criteria for causality were met. However, the evidence for two of the criteria, although present, was not as strong as it was for the other criteria. These criteria were strength of the association (criterion 1) and experiment (criterion 8), and according to Bradford Hill, these are the two criteria that can provide the strongest evidence for causality. Only a small minority of studies offered weak support for price-based alcohol policy interventions . Therefore although all of the criteria were supported, we conclude that it is highly probable, but not definite, that introducing MUP would reduce alcohol consumption and alcohol-related harms. It is also of note that different types of study tended to satisfy different Bradford Hill criteria, and that different study designs also produced evidence of the effectiveness of minimum pricing in relation to different outcomes. This is summarised in Figure 2. This underlines the importance of including a variety of study designs in this review.

[figure 2 to go here]

Strengths of this study are that this is the first to have systematically reviewed the literature relevant specifically to alcohol minimum pricing policies. We had broad inclusion criteria with regards to study design, price intervention and outcome measure, allowing for a comprehensive review of the evidence base. Application of the Bradford Hill criteria as part of a narrative systematic literature review is a useful and emergent technique for identifying causality: a PubMed search for systematic reviews with Bradford Hill' mentioned in the title or abstract yielded 28 results, 90% of which were published in the last five years. The limitations of this systematic review relate mainly to the broad range of studies included. It was not possible to conduct any kind of meta-analysis and therefore we do not present a pooled estimate for the likely effect of MUP on certain outcomes. Occasionally, minimum pricing has been implemented as part of a range of measures (e.g. (12)), and these studies were considered alongside studies where MUP was implemented in isolation. This emphasises the importance of the specificity criterion. There were also challenges with the quality appraisal. The

For peer review only - http://bmjopen.bmj.com/site/about/guidelines.xhtml

EPHPP quality assessment tool was used to assess studies. However it was not possible to appraise two of the studies from the grey literature using this tool, and there were some challenges assessing the econometric modelling studies against this framework. However overall we think that our quality appraisal across the different studies is broadly comparable. It should also be noted that although a number of studies were rated as 'strong', this is in relation to their respective study designs and does not reflect the position of the study type in the hierarchy of evidence framework.

This is the first systematic review that has addressed the effectiveness of price-based interventions for alcohol such as MUP using the Bradford Hill criteria. It was beyond the scope of this review to study the impact of generalised increases in alcohol prices (as opposed to minimum prices). However where such studies have been done, a minimum price or floor price has been recommended, for example in Gruenewald's 2006 study in Sweden which found that the lowest quality (the cheapest) alcohol has the highest price elasticity (46). Previous systematic reviews of alcohol price and consumption (34) and alcohol-related harm (35) have tended to consider the effect of price increases and increased taxation together. These reviews found significant effects on consumption and morbidity and mortality. Although price regulation and taxation are closely related policy options, evidence from surveys (45) and modelling studies (36) suggests that the effects of each are somewhat different, and this is why we chose to study only price in this review. It is important to highlight that a considerable proportion of included studies were produced by a small number of research teams. Also, with regards to the econometric modelling studies, uncertainty in estimates or forecasts is often poorly communicated outside of the academic literature. The overall risk of bias in the included studies was minimised by excluding studies with a conflict of interest (either for or against MUP). It was not possible to assess publication bias using an analytical technique such as a funnel plot due to the narrative nature of the review, however we anticipate that by including grey literature in this review we have mitigated publication bias as far as reasonably possible.

Overall the findings of this review lend strong support for policies such as MUP in reducing alcohol consumption and alcohol-related harm, with all nine of the Bradford Hill criteria met and little by way of counter findings. As it is unlikely to be feasible to conduct randomised controlled trials (RCTs) of MUP, the decision whether or not to introduce MUP will not be based on a systematic review and meta-analysis of RCTs, and therefore this synthesis of the evidence base according to the Bradford Hill criteria is of value.

Unanswered questions about the effectiveness of MUP remain, and in Scotland in particular, there are opportunities to address these. The Scottish Courts of Session in Edinburgh recently stated that minimum pricing is a proportionate measure and does not contravene EU law (47), however the Scotch Whisky Association intends to appeal to the Supreme Court (48).. If Scotland now rules to implement MUP, then it would be possible to evaluate the validity of the Sheffield Alcohol Policy Model studies conducted using Scottish data. It would also be possible to conduct a longitudinal study to evaluate the effectiveness of MUP in reducing alcohol consumption and alcohol-related morbidity and mortality. The findings of this natural experiment would have relevance elsewhere within and outside the UK.

ACKNOWLEDGEMENTS

For peer review only - http://bmjopen.bmj.com/site/about/guidelines.xhtml

We would like to thank Rebecca McDonald for advice on using the Bradford Hill criteria in a systematic review, Dr James Nicholls for advice on study interpretation, and Dr Daniel Stahl for statistical advice on some of the included studies.

CONTRIBUTIONS

SM conceived the idea. SB conducted the initial search. SM and SM contributed to independently reviewing abstracts, hand-searching reference lists, completing data extraction, and conducting quality appraisal. All authors contributed to the analysis and interpretation of the results and contributed to writing the manuscript. SB is guarantor.

FUNDING

This research received no specific grant from any funding agency in the public, commercial or not-for-profit sectors.

COMPETING INTERESTS

All authors have completed the ICMJE uniform disclosure form at www.icmje.org/coi_disclosure.pdf and declare: no support from any organisation for the submitted work; no financial relationships with any organisations that might have an interest in the submitted work in the previous three years; no other relationships or activities that could appear to have influenced the submitted work.

Two of the authors work at King's College London, which as an institution is listed as a member of the Alcohol Health Alliance. SM has received funding indirectly from UKCTAS, which as an institution is also listed as a member of the Alcohol Health Alliance. However none of the authors have any relationship with the Alcohol Health Alliance.

For peer review only

For peer review only - http://bmjopen.bmj.com/site/about/guidelines.xhtml

FIGURE LEGENDS

Figure 1: PRISMA 2009 flow diagram of studies in this systematic review

Figure 2: This model shows that different study types tended to produce evidence of effectiveness on minimum pricing in relation to different outcomes. Studies cited in the figure are key examples of the literature in that area and do not represent an exhaustive list.

REFERENCES

1. Lifestyles Statistics Team,, Health and Social Care Information Centre. Statistics on Alcohol. England, 2015 [Internet]. 2015 [cited 2016 Jun 3]. Available from: http://www.hscic.gov.uk/catalogue/PUB17712/alc-eng-2015-rep.pdf

2. Anderson P, Chisholm D, Fuhr DC. Effectiveness and cost-effectiveness of policies and programmes to reduce the harm caused by alcohol. Lancet Lond Engl. 2009 Jun 27;373(9682):2234–46.

3. Stockwell T, Auld MC, Zhao J, Martin G. Does minimum pricing reduce alcohol consumption? The experience of a Canadian province. Addiction. 2012 May 1;107(5):912–20.

4. World Health Organization. European status report on alcohol and health 2014. Pricing policies [Internet]. World Health Organization; 2014. Available from: http://www.euro.who.int/__data/assets/pdf_file/0003/244902/Pricing-policies.pdf

5. HM Government. The Government's Alcohol Strategy [Internet]. 2012 [cited 2016 Jun 3]. Available from: https://www.gov.uk/government/uploads/system/uploads/attachment_data/file/224075/alcohol-strategy.pdf

6. Home Office. Guidance on banning the sale of alcohol below the cost of duty plus VAT. For suppliers of alcohol and enforcement authorities in England and Wales [Internet]. 2015 [cited 2016 Jun 3]. Available from: https://www.gov.uk/government/uploads/system/uploads/attachment_data/file/415522/HO_Guidance_on_BBCS.pdf

7. Off Licence News. First conviction for selling alcohol "below cost" sees retailer fined [Internet]. 2016 [cited 2016 Jun 3]. Available from: http://www.offlicencenews.co.uk/news/fullstory.php/aid/15750/First_conviction_for_selling_alcohol__below_cost__sees_retailer_fined.html

8. Home Office. Duty plus VAT permitted price calculator (2015) [Internet]. 2016. Available from: https://www.gov.uk/government/publications/banning-the-sale-of-alcohol-below-the-cost-of-duty-plus-vat-march-2015

9. Fitzgerald N, Angus C. Four Nations: How Evidence-based are Alcohol Policies and Programmes across the UK?. London: [Internet]. London: Alliance for Useful Evidence/Alcohol Health Alliance.; 2015. Available from: http://www.alliance4usefulevidence.org/assets/Four-Nations-v3.pdf

For peer review only

For peer review only - http://bmjopen.bmj.com/site/about/guidelines.xhtml

10. Cochrane Handbook. 21.4 Assessment of study quality and risk of bias [Internet]. [cited 2016 Jun 2]. Available from: http://handbook.cochrane.org/chapter_21/21_4_assessment_of_study_quality_and_risk_of_bias.htm

11. Hill AB. The Environment and Disease: Association or Causation? Proc R Soc Med. 1965 May;58(5):295–300.

12. Bhattacharya J, Gathmann C, Miller G. The Gorbachev Anti-Alcohol Campaign and Russia's Mortality Crisis. Am Econ J Appl Econ. 2013;5(2):232–60.

13. Herttua K, Mäkelä P, Martikainen P. Minimum Prices for Alcohol and Educational Disparities in Alcohol-related Mortality. Epidemiology. 2015 May;26(3):337–43.

14. Stockwell T, Zhao J, Giesbrecht N, Macdonald S, Thomas G, Wettlaufer A. The Raising of Minimum Alcohol Prices in Saskatchewan, Canada: Impacts on Consumption and Implications for Public Health. Am J Public Health. 2012 Oct 18;102(12):e103–10.

15. Stockwell T, Zhao J, Martin G, Macdonald S, Vallance K, Treno A, et al. Minimum Alcohol Prices and Outlet Densities in British Columbia, Canada: Estimated Impacts on Alcohol-Attributable Hospital Admissions. Am J Public Health. 2013 Apr 18;103(11):2014–20.

16. Treisman D. Death and prices. Econ Transit. 2010 Apr 1;18(2):281–331.

17. Wald I, Moskalewicz J. Alcohol policy in a crisis situation. Br J Addict. 1984 Sep;79(3):331–5.

18. Zhao J, Stockwell T, Martin G, Macdonald S, Vallance K, Treno A, et al. The relationship between minimum alcohol prices, outlet densities and alcohol-attributable deaths in British Columbia, 2002–09. Addiction. 2013 Jun 1;108(6):1059–69.

19. Brennan A, Meng Y, Holmes J, Hill-McManus D, Meier PS. Potential benefits of minimum unit pricing for alcohol versus a ban on below cost selling in England 2014: modelling study. The BMJ. 2014 Sep 30;349:g5452.

20. Holmes J, Meng Y, Meier PS, Brennan A, Angus C, Campbell-Burton A, et al. Effects of minimum unit pricing for alcohol on different income and socioeconomic groups: a modelling study. The Lancet. 2014 May 16;383(9929):1655–64.

21. Meier PS, Purshouse R, Brennan A. Policy options for alcohol price regulation: the importance of modelling population heterogeneity. Addiction. 2010 Mar 1;105(3):383–93.

22. Purshouse RC, Meier PS, Brennan A, Taylor KB, Rafia R. Estimated effect of alcohol pricing policies on health and health economic outcomes in England: an epidemiological model. The Lancet. 2010 Apr;375(9723):1355–64.

23. Black H, Gill J, Chick J. The price of a drink: levels of consumption and price paid per unit of alcohol by Edinburgh's ill drinkers with a comparison to wider alcohol sales in Scotland. Addiction. 2011 Apr 1;106(4):729–36.

For peer review only

For peer review only - http://bmjopen.bmj.com/site/about/guidelines.xhtml

24. Callinan S, Room R, Livingston M, Jiang H. Who Purchases Low-Cost Alcohol in Australia? Alcohol Alcohol. 2015 Jun 24;agv066.

25. Casswell S, Huckle T, Wall M, Yeh LC. International alcohol control study: pricing data and hours of purchase predict heavier drinking. Alcohol Clin Exp Res. 2014 May;38(5):1425–31.

26. Crawford MJ, Parry AMH, Weston ARW, Seretis D, Zauter-Tutt M, Hussain A, et al. Relationship between price paid for off-trade alcohol, alcohol consumption and income in England: a cross-sectional survey. Alcohol Alcohol Oxf Oxfs. 2012 Dec;47(6):738–42.

27. Falkner C, Christie G. The effect of alcohol price on dependent drinkers' alcohol consumption. N Z Med J. 2015;128(1427).

28. Forsyth AJM, Ellaway A, Davidson N. How Might the Alcohol Minimum Unit Pricing (MUP) Impact upon Local Off-Sales Shops and the Communities Which They Serve? Alcohol Alcohol. 2014 Jan 1;49(1):96–102.

29. Ludbrook PA, Petrie D, McKenzie L, Farrar S. Tackling alcohol misuse: Purchasing patterns affected by minimum pricing for alcohol. Appl Health Econ Health Policy. 2012 Aug 6;10(1):51–63.

30. Sharma A, Vandenberg B, Hollingsworth B. Minimum Pricing of Alcohol versus Volumetric Taxation: Which Policy Will Reduce Heavy Consumption without Adversely Affecting Light and Moderate Consumers? PLoS ONE. 2014 Jan 22;9(1):e80936.

31. Sheron N, Chilcott F, Matthews L, Challoner B, Thomas M. Impact of minimum price per unit of alcohol on patients with liver disease in the UK. Clin Med. 2014 Aug 1;14(4):396–403.

32. Babor TF, Mendelson JH, Greenberg I, Kuehnle J. Experimental analysis of the 'happy hour": effects of purchase price on alcohol consumption. Psychopharmacology (Berl). 1978 Jun 15;58(1):35–41.

33. Seaman P, Edgar F, Ikegwuonu T. The role of alcohol price in young adult drinking cultures in Scotland. Drugs Educ Prev Policy. 2013 Aug 1;20(4):278–85.

34. Wagenaar AC, Salois MJ, Komro KA. Effects of beverage alcohol price and tax levels on drinking: a meta-analysis of 1003 estimates from 112 studies. Addict Abingdon Engl. 2009 Feb;104(2):179–90.

35. Wagenaar AC, Tobler AL, Komro KA. Effects of alcohol tax and price policies on morbidity and mortality: a systematic review. Am J Public Health. 2010 Nov;100(11):2270–8.

36. Angus C, Holmes J, Pryce R, Meier P, Brennan A. Model-based appraisal of the comparative impact of Minimum Unit Pricing and taxation policies in Scotland An adaptation of the Sheffield Alcohol Policy Model version 3 [Internet]. ScHARR, University of Sheffield; 2016 Apr [cited 2016 Apr 7]. Available from: https://www.shef.ac.uk/polopoly_fs/1.565373!/file/Scotland_report_2016.pdf

For peer review only - http://bmjopen.bmj.com/site/about/guidelines.xhtml

37. Booth A, Brennan A, Meier PS, O'Reilly D, Purshouse R, Stockwell T, et al. Independent review of the effects of alcohol pricing and promotion: part A – systematic reviews. Project Report for the Department of Health September 2008. ScHARR University of Sheffield; 2008.

38. Brennan A, Purshouse R, Taylor K, Rafia R. Independent review of the effects of alcohol pricing and promotion: part B. Modelling the Potential Impact of Pricing and Promotion Policies for Alcohol in England: Results from the Sheffield Alcohol Policy Model Version 2008(1-1) [Internet]. ScHARR University of Sheffield; 2008. Available from: https://www.shef.ac.uk/polopoly_fs/1.95621!/file/PartB.pdf

39. Hill McManus D, Brennan A, Stockwell T, Giesbrecht N, Thomas G, Zhao J, et al. Model-based appraisal of alcohol minimum pricing in Ontario and British Columbia: A Canadian adaptation of the Sheffield Alcohol Policy Model Version 2 [Internet]. 2012. Available from: http://www.uvic.ca/research/centres/carbc/assets/docs/report-model-based-appraisal.pdf

40. Institute for Fiscal Studies. The Impact of Introducing a Minimum Price on Alcohol in Britain. IFS Briefing Note BN109. 2010.

41. Institute for Fiscal Studies. Price-based measures to reduce alcohol consumption. IFS Briefing Note BN138. 2013.

42. Meng Y, Purshouse R, Brennan A, Meier PS. Model-based appraisal of alcohol minimum pricing and off-licensed trade discount bans in Scotland using the Sheffield Alcohol Policy Model (v.2): Second update based on newly available data [Internet]. 2010. Available from: http://www.shef.ac.uk/polopoly_fs/1.96510!/file/scotlandupdate.pdf

43. Rabinovich L, Brutscher P-B, de Vries H, Tiessen J, Clift J, Reding A. The affordability of alcoholic beverages in the European Union. 2012;

44. Gallet CA. The Demand for Alcohol: A Meta-Analysis of Elasticities [Internet]. Rochester, NY: Social Science Research Network; 2007 May [cited 2016 Jan 6]. Report No.: ID 985689. Available from: http://papers.ssrn.com/abstract=985689

45. Ally AK, Meng Y, Chakraborty R, Dobson PW, Seaton JS, Holmes J, et al. Alcohol tax pass-through across the product and price range: do retailers treat cheap alcohol differently? Addiction. 2014 Dec 1;109(12):1994–2002.

46. Gruenewald PJ, Ponicki WR, Holder HD, Romelsjö A. Alcohol prices, beverage quality, and the demand for alcohol: quality substitutions and price elasticities. Alcohol Clin Exp Res. 2006 Jan;30(1):96–105.

47. Judiciary of Scotland. Scotch Whisky Association and others v Lord Advocate and Advocate General for Scotland [Internet]. [cited 2016 Jul 11]. Available from: http://www.scotland-judiciary.org.uk/9/1672/Scotch-Whisky-Association-and-others-v-Lord-Advocate-and-Advocate-General-for-Scotland

48. Scotch Whisky Association. The SWA on its decision to appeal minimum unit pricing (MUP) ruling [Internet]. 2016. Available from: http://www.scotch-whisky.org.uk/news-publications/news/the-swa-on-its-decision-to-appeal-minimum-unit-pricing-(mup)-ruling/#.WDMngbKLSM_

For peer review only

For peer review only - http://bmjopen.bmj.com/site/about/guidelines.xhtml

For peer review only

For peer review only - http://bmjopen.bmj.com/site/about/guidelines.xhtml

PRISMA 2009 Flow Diagram

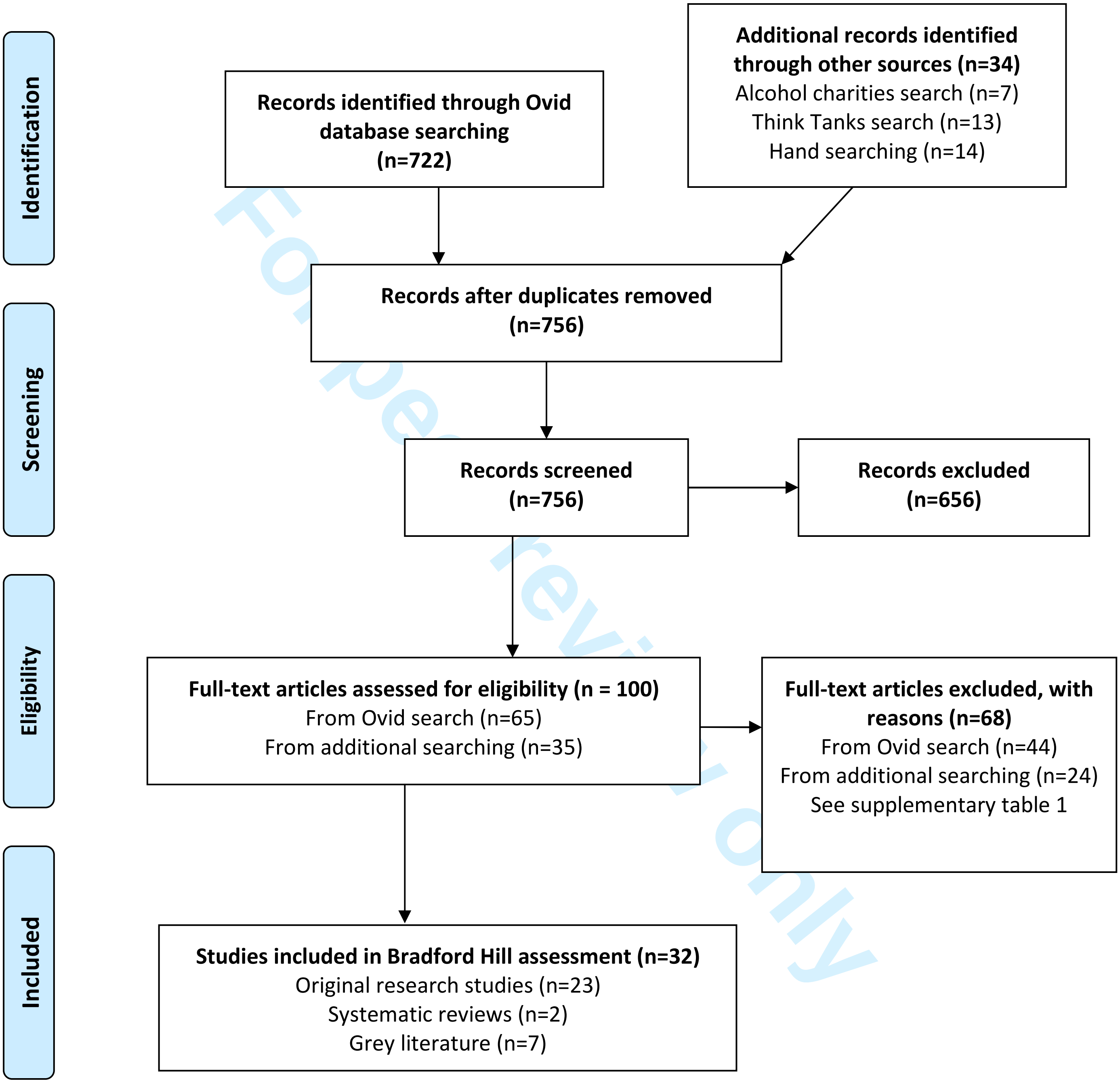

From: Moher D, Liberati A, Tetzlaff J, Altman DG, The PRISMA Group (2009). *P*referred *R*eporting *I*tems for *S*ystematic Reviews and *M*eta-*A*nalyses: The PRISMA Statement. PLoS Med 6(6): e1000097. doi:10.1371/journal.pmed1000097

For more information, visit www.prisma-statement.org.

For peer review only - http://bmjopen.bmj.com/site/about/guidelines.xhtml

Figure 2: Areas of evidence for the effectiveness of minimum pricing of alcohol, with key examples

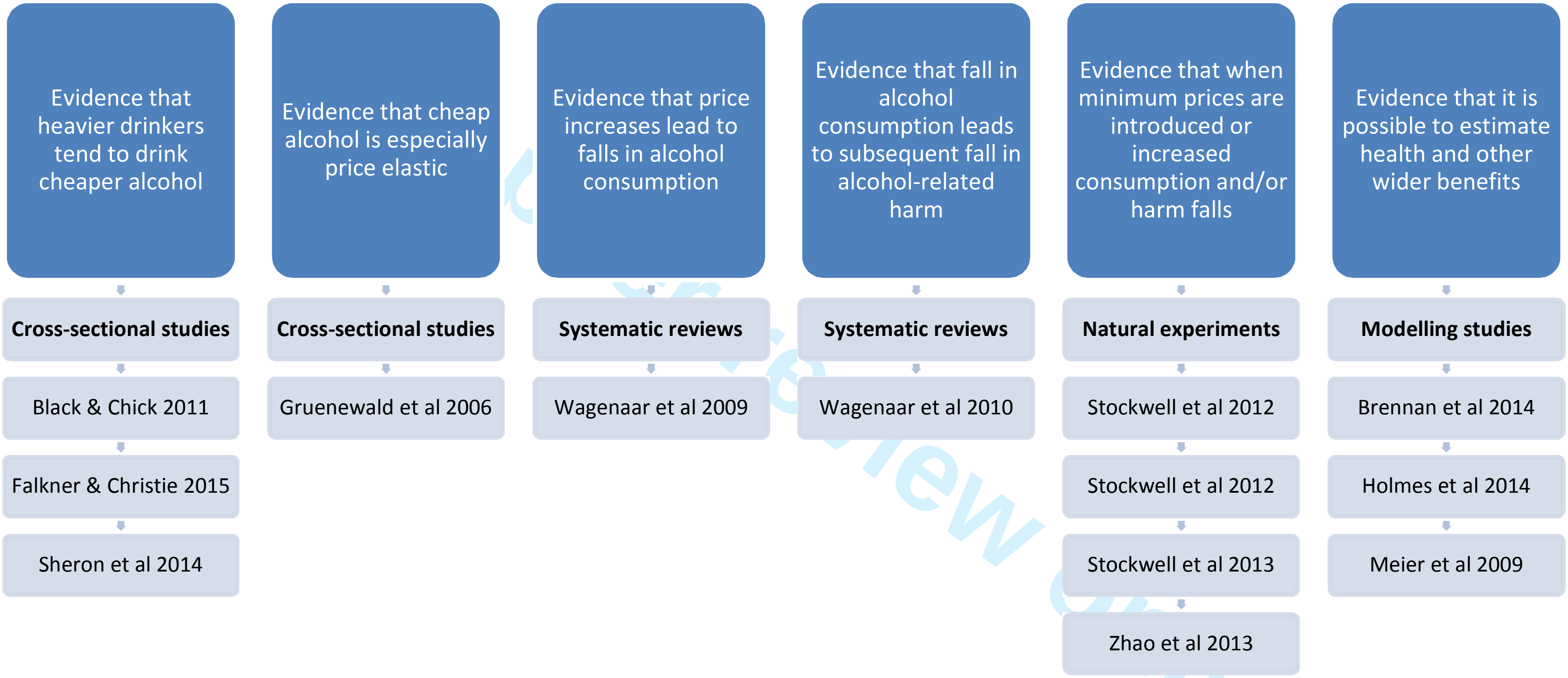

Online supplementary table: identified studies where full text assessed, then not included in review

Source	Author and year published	Study type	Reason excluded
Ovid search	Aage 2012 (1)	Time series analysis	Affordability, not price
	Ayyagari 2013 (2)	Econometric analysis of price and consumption data	Price elasticities of demand only
	Bellis 2009 (3)	Cross-sectional surveys	Association between drink type and price, and drink type and harms, but not directly reported in paper
	Bennetts 2008 (4)	Review (not systematic)	Review of a report (not a research study)
	Callinan 2015 (5)	Editorial (not a research study)	Editorial about different pricing, taxation and afforability measures, not a research study
	Chalmers 2013 (6)	Commentary (not a research study)	Is a commentary about challenges to MUP e.g. industry and the law
	Chaloupka 2002 (7)	Review (not systematic)	Review of previous economic studies (not systematic)
	Chick 2012 (8)	Editorial (not a research study)	Is about bans on multi-buys, not minimum price
	Cook 2014 (9)	Analysis of cross-sectional survey data and alcohol policies	Affordability not price (GDP PPP)
	Duffy 1981 (10)	Econometric time series analysis	Talks about elasticities in relation to duty rises not MUP. NB is M duffy, not J duffy
	Giesbrecht 2003 (11)	Review (not systematic)	Review of previous studies (not systematic)
	Giesbrecht 2015 (12)	Policy analysis	Does not relate price intervention to consumption or harm
	Gilligan 2012 (13)	Cross-sectional survey and policy analysis	Price changes, not minimum prices
	Grossman 2004 (14)	Economic analysis	Tax (federal excise tax increase) not MUP
	Hadland 2015 (15)	Policy analysis	Effects of numerous alcohol policies considered together
	Heeb 2003 (16)	Natural experiment: longitudinal survey 3 months before and 3 months after price change	Tax, not price intervention

For peer review only - http://bmjopen.bmj.com/site/about/guidelines.xhtml

	Hogan 2006 (17)	Natural experiment/evaluation	Tax, not price intervention
	Jónsson 2013 (18)	Cross-sectional surveys and routine data	Policies included tax and other supply side policies, but not MUP
	Katikireddi 2012 (19)	Commentary (not a research study)	Is a commentary about legal considerations of MUP, not effectiveness
	Katikireddi 2014 (20)	Qualitative study	Stakeholder views of MUP, not effectiveness
	Khaltourina 2015 (21)	Cross-sectional surveys and routine data	Tax, not price intervention
	Knibbe 2014 (22)	Analysis of cross-sectional survey data and alcohol policies	No price intervention studied, price investigated more as a covariate
	Lindeman 2013 (23)	Cross-sectional surveys and routine data	Tax, not price intervention
	Lonsdale 2012 (24)	Qualitative study	Public opinion of policy, nothing about effectiveness
	Mäkelä 2009 (25)	Review (not systematic) and analysis of routine data	Tax, not price intervention
	McCambridge 2014 (26)	Document analysis and qualitative interviews	Corporate lobbying, not the effectiveness of MUP
	Meng 2014 (27)	Econometric analysis using pseudo panel	Price elasticities of demand only
	Nelson 2013 (28)	Review (not systematic)	Conflict of interest (funded by International Center for Alcohol Policies).
	Nelson 2014 (affordability) (29)	Analysis of routine data and economic modelling	Affordability not price, and conflict of interest (funded by International Center for Alcohol Policies).
	Nelson 2014 (elasticities) (30)	Economic modelling	Conflict of interest (funded by International Center for Alcohol Policies).
	Nelson 2014 (gender) (31)	Systematic review	Conflict of interest (funded by International Center for Alcohol Policies).
	Nicholls & Greenaway 2015 (32)	Policy analysis	Is about policy framing, not the effectiveness of MUP
	Record 2009 (33)	Modelling study	Conflict of interest – both authors were members of the Alcohol Health Alliance which is campaigning for MUP
	Rush 1986 (34)	Analysis of routine data	Affordability, not price

For peer review only - http://bmjopen.bmj.com/site/about/guidelines.xhtml

	Shi 2011 (35)	Econometric analysis of price and consumption data	Price elasticities of demand only
	Skov 2011 (36)	Natural experiment	Tax, not price intervention
	Sloan 1994 (37)	Analysis of routine data 1982-1988	Price changes, not minimum prices
	Stockwell 2012 (38)	Letter to journal (not a research study)	Letter concerning homelessness and price paid for alcohol
	Stockwell 2013 (39)	Letter to journal (not a research study)	Letter to editor about UK industry criticism of Canadian research on MUP, not a research study
	Sutton & Godfrey 1995 (40)	Cross-sectional survey	Price changes, not minimum prices
	Tian 2011 (41)	Econometric analysis of price and consumption data	Price elasticities of demand only
	Wall & Casswell 2013 (42)	Time series analysis and econometric modelling	Price changes, not minimum prices
	Xuan 2015 (43)	Analysis of cross-sectional survey data and alcohol policies	Price and tax considered together
	Yashkin 2013 (44)	Econometric analysis of price and consumption data	Price elasticities of demand only
Hand searching & grey literature	Ally 2014 (45)	Analysis of VAT and price data	Tax, not price intervention
	Anderson 2009 (46)	Lancet 'Series' article – a commentary/review	Not an original research study.
	Byrnes 2013 (47)	Repeated cross-sectional survey	Price changes, not minimum prices
	Centre for Economics and Business Research 2010 (48)	Report (not a research study)	Conflict of interest (funded by SAB Miller). Is a critique of Sheffield modelling, not a research study.
	Craven 2013 (49)	Economic report	Published in the journal of the Institute of Economic Affairs, which has a conflict of interest.
	Duffy & Snowdon 2012 (50)	Report chapters (not a research study)	Is a critique of Sheffield modelling, not a research study.
	Fitzgerald & Angus 2015	Report (not a research study)	Is a report about use of evidence in policymaking, not

For peer review only - http://bmjopen.bmj.com/site/about/guidelines.xhtml

	(51)		about effectiveness of MUP. Also not a research study.
	Gallet 2007 (52)	Meta-analysis	Is looking at elasticities of demand for alcohol, not the effectiveness of MUP.
	Gray 2000 (53)	Natural experiment	Restrictions do not include any price interventions
	Gruenewald 2006 (54)	Time series analysis/modelling	Price changes, not minimum prices, but included in narrative
	Hilton 2014 (55)	Content analysis of UK newsprint	Media representations of MUP, not effectiveness
	Home Office 2011 (56)	'Summary review'/report (not a research study)	Replicates what we have elsewhere, but in less detail. Also not a research study.
	Institute for Fiscal Studies 2011 (57)	Report (not a research study)	Is not about effectiveness of MUP
	Institute for Fiscal Studies 2013 (58)	Report (not a research study)	Is not looking at effectiveness of MUP, is comparing Govt and industry revenue under MUP and higher tax
	Institute of Alcohol Studies (Goodliffe) 2014 (59)	Webpage	Is discussing legal issues, not effectiveness of MUP
	Kisely & Lawence 2015 (60)	Natural experiment	Tax, not price intervention
	Ludbrook 2010 (61)	Secondary analysis of Expenditure and Food Survey	Describes purchasing patterns of low price alcohol in Scotland. Not about MUP.
	Ornstein 1983 (62)	Literature review book chapter (not systematic)	Price elasticities of demand only
	Rabinovch 2012 (63)	Technical report (contains data)	Does not assess effectiveness of MUP
	Radaev 2015 (64)	Time series analysis	Paper explored the effect of price interventions on consumption of homemade alcohol, not the effectiveness of MUP
	Robinson 2013 (65)	Letter to journal (not a research study)	Letter to editor about evidence for MUP, not a research study
	Snowdon 2015 (66)	Book chapter	Is a critique of Sheffield modelling, not a research study. Institute of Economic Affairs also has a conflict of interest.

For peer review only - http://bmjopen.bmj.com/site/about/guidelines.xhtml

	Wine and Spirits Trade Association n.d. (67)	Press release	Press release critiquing Zhao 2013 paper, not a research study
	World Health Organisation 2014 (68)	Report	Does not discuss effectiveness of MUP

For peer review only

For peer review only - http://bmjopen.bmj.com/site/about/guidelines.xhtml

References

1. Aage H. Alcohol in Greenland 1951–2010: consumption, mortality, prices. Int J Circumpolar Health [Internet]. 2012 Dec 17 [cited 2016 Feb 27];71. Available from: http://www.ncbi.nlm.nih.gov/pmc/articles/PMC3525923/

2. Ayyagari P, Deb P, Fletcher J, Gallo W, Sindelar JL. Understanding heterogeneity in price elasticities in the demand for alcohol for older individuals. Health Econ. 2013 Jan;22(1):89–105.

3. Bellis MA, Phillips-Howard PA, Hughes K, Hughes S, Cook PA, Morleo M, et al. Teenage drinking, alcohol availability and pricing: a cross-sectional study of risk and protective factors for alcohol-related harms in school children. BMC Public Health. 2009 Oct 9;9(1):1–12.

4. Bennetts R. Russell Bennetts. Alcohol: Price, Policy and Public Health. Alcohol Alcohol. 2008 Mar 1;43(2):123–123.

5. Callinan S, Room R, Dietze P. Alcohol Price Policies as an Instrument of Health Equity: Differential Effects of Tax and Minimum Price Measures. Alcohol Alcohol Oxf Oxfs. 2015 Nov;50(6):629–30.

6. Chalmers J, Carragher N, Davoren S, O'Brien P. Real or perceived impediments to minimum pricing of alcohol in Australia: Public opinion, the industry and the law. Int J Drug Policy. 2013 Nov;24(6):517–23.

7. Chaloupka FJ, Grossman M, Saffer H. The effects of price on alcohol consumption and alcohol-related problems. Alcohol Res Health J Natl Inst Alcohol Abuse Alcohol. 2002;26(1):22–34.

8. Chick J. 16 for the Price of 10: Effects of a Ban on Multi-Buy Alcohol. Alcohol Alcohol. 2012 Feb 22;47(2):83–83.

9. Cook WK, Bond J, Greenfield TK. Are alcohol policies associated with alcohol consumption in low- and middle-income countries? Addict Abingdon Engl. 2014 Jul;109(7):1081–90.

10. Duffy M. The influence of prices, consumer incomes and advertising upon the demand for alcoholic drink in the United Kindgom: an econometric study. Alcohol Alcohol. 1981 Sep 21;16(4):200–9.

11. Giesbrecht N, Greenfield TK. Preventing Alcohol-Related Problems in the US Through Policy: Media Campaigns, Regulatory Approaches and Environmental Interventions. J Prim Prev. 2003 Sep;24(1):63–104.

12. Giesbrecht N, Wettlaufer A, Thomas G, Stockwell T, Thompson K, April N, et al. Pricing of alcohol in Canada: A comparison of provincial policies and harm-reduction opportunities. Drug Alcohol Rev. 2015 Nov 4;

13. Gilligan C, Kuntsche E, Gmel G. Adolescent drinking patterns across countries: associations with alcohol policies. Alcohol Alcohol Oxf Oxfs. 2012 Dec;47(6):732–7.

For peer review only - http://bmjopen.bmj.com/site/about/guidelines.xhtml

14. Grossman M. Individual Behaviors and Substance Use: The Role of Price [Internet]. National Bureau of Economic Research; 2004 Dec [cited 2016 Feb 27]. Report No.: 10948. Available from: http://www.nber.org/papers/w10948

15. Hadland SE, Xuan Z, Blanchette JG, Heeren TC, Swahn MH, Naimi TS. Alcohol Policies and Alcoholic Cirrhosis Mortality in the United States. Prev Chronic Dis [Internet]. 2015 Oct 15 [cited 2016 Feb 27];12. Available from: http://www.cdc.gov/pcd/issues/2015/15_0200.htm

16. Heeb J-L, Gmel G, Zurbrügg C, Kuo M, Rehm J. Changes in alcohol consumption following a reduction in the price of spirits: a natural experiment in Switzerland. Addict Abingdon Engl. 2003 Oct;98(10):1433–46.

17. Hogan E, BOFFA J, ROSEWARNE C, BELL S, CHEE DA. What price do we pay to prevent alcohol-related harms in Aboriginal communities? The Alice Springs trial of liquor licensing restrictions. Drug Alcohol Rev. 2006;25(3):207–212.

18. Jónsson RM, Kristjánsson S. Alcohol policy and public opinion in Iceland, 1989–2012. Nord Stud Alcohol Drugs. 2013;30(6):539–549.

19. Katikireddi SV, McLean JA. Introducing a minimum unit price for alcohol in Scotland: considerations under European Law and the implications for European public health. Eur J Public Health. 2012 Aug 1;22(4):457–8.

20. Katikireddi SV, Bond L, Hilton S. Perspectives on econometric modelling to inform policy: a UK qualitative case study of minimum unit pricing of alcohol. Eur J Public Health. 2014 Jun 1;24(3):490–5.

21. Khaltourina D, Korotayev A. Effects of Specific Alcohol Control Policy Measures on Alcohol-Related Mortality in Russia from 1998 to 2013. Alcohol Alcohol. 2015 Sep 1;50(5):588–601.

22. Knibbe RA, Derickx M, Allamani A, Massini G. Alcohol Consumption and its Related Harms in the Netherlands Since 1960: Relationships With Planned and Unplanned Factors. Subst Use Misuse. 2014 Oct 15;49(12):1589–600.

23. Lindeman M, Karlsson T, Österberg E. Public opinions, alcohol consumption and policy changes in Finland, 1993–2013. Nord Stud Alcohol Drugs. 2013;30(6):507–524.

24. Lonsdale AJ, Hardcastle SJ, Hagger MS. A minimum price per unit of alcohol: A focus group study to investigate public opinion concerning UK government proposals to introduce new price controls to curb alcohol consumption. BMC Public Health. 2012 Nov 23;12(1):1023.

25. Mäkelä P, Osterberg E. Weakening of one more alcohol control pillar: a review of the effects of the alcohol tax cuts in Finland in 2004. Addict Abingdon Engl. 2009 Apr;104(4):554–63.

26. McCambridge J, Hawkins B, Holden C. Vested Interests in Addiction Research and Policy. The challenge corporate lobbying poses to reducing society's alcohol problems: insights from UK evidence on minimum unit pricing. Addiction. 2014 Feb 1;109(2):199–205.

For peer review only - http://bmjopen.bmj.com/site/about/guidelines.xhtml

27. Meng Y, Brennan A, Purshouse R, Hill-McManus D, Angus C, Holmes J, et al. Estimation of own and cross price elasticities of alcohol demand in the UK--A pseudo-panel approach using the Living Costs and Food Survey 2001-2009. J Health Econ. 2014 Mar;34:96–103.

28. Nelson JP. Does Heavy Drinking by Adults Respond to Higher Alcohol Prices and Taxes? A Survey and Assessment. Econ Anal Policy. 2013 Dec;43(3):265–91.

29. Nelson JP. Alcohol Affordability and Alcohol Demand: Cross-Country Trends and Panel Data Estimates, 1975 to 2008. Alcohol Clin Exp Res. 2014 Apr 1;38(4):1167–75.

30. Nelson JP. Estimating the Price Elasticity of Beer: Meta-Analysis of Data with Heterogeneity, Dependence, and Publication Bias [Internet]. Rochester, NY: Social Science Research Network; 2013 Jan [cited 2016 Feb 27]. Report No.: ID 2200492. Available from: http://papers.ssrn.com/abstract=2200492

31. Nelson JP. Gender differences in alcohol demand: a systematic review of the role of prices and taxes. Health Econ. 2014 Oct;23(10):1260–80.

32. Nicholls J, Greenaway J. What is the problem?: Evidence, politics and alcohol policy in England and Wales, 2010–2014. Drugs Educ Prev Policy. 2015 Mar 4;22(2):135–42.

33. Record C, Day C. Britain's alcohol market: how minimum alcohol prices could stop moderate drinkers subsidising those drinking at hazardous and harmful levels. Clin Med. 2009 Oct 1;9(5):421–5.

34. Rush B, Steinberg M, Brook R. The relationships among alcohol availability, alcohol consumption and alcohol-related damage in the Province of Ontario and the State of Michigan 1955-1982. Adv Alcohol Subst Abuse. 1986;5(4):33–45.

35. Shi Y. Three Essays on Economics of Health Behavior in China [Internet]. 2011 [cited 2016 Mar 20]. Available from: http://www.rand.org/pubs/rgs_dissertations/RGSD287.html

36. Skov SJ, Chikritzhs TN, Kypri K, Miller PG, Hall WD, Daube MM, et al. Is the "alcopops" tax working? Probably yes but there is a bigger picture. Med J Aust [Internet]. 2011 [cited 2015 Nov 9];195(2). Available from: https://www.mja.com.au/journal/2011/195/2/alcopops-tax-working-probably-yes-there-bigger-picture

37. Sloan FA, Reilly BA, Schenzler C. Effects of prices, civil and criminal sanctions, and law enforcement on alcohol-related mortality. J Stud Alcohol. 1994 Jul;55(4):454–65.

38. Stockwell T, Williams N, Pauly B. Working and waiting: Homeless drinkers responses to less affordable alcohol. Drug Alcohol Rev. 2012 Sep 1;31(6):823–4.

39. Stockwell T, Zhao J, Martin G, Macdonald S, Vallance K, Treno A, et al. Misleading UK alcohol industry criticism of Canadian research on minimum pricing. Addiction. 2013 Jun 1;108(6):1172–3.

40. Sutton M, Godfrey C. A grouped data regression approach to estimating economic and social influences on individual drinking behaviour. Health Econ. 1995 May 1;4(3):237–47.

For peer review only - http://bmjopen.bmj.com/site/about/guidelines.xhtml

41. Tian G, Liu F. Is the demand for alcoholic beverages in developing countries sensitive to price? Evidence from China. Int J Environ Res Public Health. 2011 Jun;8(6):2124–31.

42. Wall M, Casswell S. Affordability of alcohol as a key driver of alcohol demand in New Zealand: a co-integration analysis. Addict Abingdon Engl. 2013 Jan;108(1):72–9.

43. Xuan Z, Blanchette J, Nelson TF, Heeren T, Oussayef N, Naimi TS. The alcohol policy environment and policy subgroups as predictors of binge drinking measures among US adults. Am J Public Health. 2015 Apr;105(4):816–22.

44. Yashkin A. The Dynamics of Alcohol Consumption in the Russian Federation: Implications of Using Price Related Policies to Control Alcohol Use. Grad Theses Diss [Internet]. 2013 Jan 1; Available from: http://scholarcommons.usf.edu/etd/4968

45. Ally AK, Meng Y, Chakraborty R, Dobson PW, Seaton JS, Holmes J, et al. Alcohol tax pass-through across the product and price range: do retailers treat cheap alcohol differently? Addiction. 2014 Dec 1;109(12):1994–2002.

46. Anderson P, Chisholm D, Fuhr DC. Effectiveness and cost-effectiveness of policies and programmes to reduce the harm caused by alcohol. Lancet Lond Engl. 2009 Jun 27;373(9682):2234–46.

47. Byrnes J, Shakeshaft A, Petrie D, Doran C. Can harms associated with high-intensity drinking be reduced by increasing the price of alcohol? Drug Alcohol Rev. 2013 Jan;32(1):27–30.

48. Centre for Economics and Business Research. Minimum Alcohol Pricing: A targeted measure? Report to the Scottish Parliamentary Health and Sport Committee [Internet]. London; 2010 Aug. Available from: http://www.ias.org.uk/uploads/pdf/Price%20docs/Updated-Sheffield-Scotland-v2-August-20103.pdf

49. Craven BM, Marlow ML, Shiers AF. The Economics of Minimum Pricing for Alcohol. Econ Aff. 2013 Jun 1;33(2):174–89.

50. Duffy J, Snowdon C. The minimal evidence for minimum pricing [Internet]. Adam Smith Institute; Available from: http://www.adamsmith.org/sites/default/files/research/files/ASI_SAPM.pdf

51. Fitzgerald N, Angus C. Four Nations: How Evidence-based are Alcohol Policies and Programmes across the UK?. London: [Internet]. London: Alliance for Useful Evidence/Alcohol Health Alliance.; 2015. Available from: http://www.alliance4usefulevidence.org/assets/Four-Nations-v3.pdf

52. Gallet CA. The Demand for Alcohol: A Meta-Analysis of Elasticities [Internet]. Rochester, NY: Social Science Research Network; 2007 May [cited 2016 Jan 6]. Report No.: ID 985689. Available from: http://papers.ssrn.com/abstract=985689

53. Gray D, Saggers S, Atkinson D, Sputore B, Bourbon D. Beating the grog: an evaluation of the Tennant Creek liquor licensing restrictions. Aust N Z J Public Health. 2000 Feb;24(1):39–44.

For peer review only - http://bmjopen.bmj.com/site/about/guidelines.xhtml

54. Gruenewald PJ, Ponicki WR, Holder HD, Romelsjö A. Alcohol prices, beverage quality, and the demand for alcohol: quality substitutions and price elasticities. Alcohol Clin Exp Res. 2006 Jan;30(1):96–105.

55. Hilton S, Wood K, Patterson C, Katikireddi SV. Implications for alcohol minimum unit pricing advocacy: What can we learn for public health from UK newsprint coverage of key claim-makers in the policy debate? Soc Sci Med. 2014 Feb;102:157–64.

56. Home Office. The likely impacts of increasing alcohol price: a summary review of the evidence base [Internet]. 2011 Jan. Available from: https://www.gov.uk/government/uploads/system/uploads/attachment_data/file/98100/impacts-alcohol-price-review.pdf

57. Institute for Fiscal Studies. Alcohol pricing and taxation policies. IFS Briefing Note BN124. 2011.

58. Institute for Fiscal Studies. Price-based measures to reduce alcohol consumption. IFS Briefing Note BN138. 2013.

59. Goodliffe J. Applying a minimum price to alcohol (Institute of Alcohol Studies) [Internet]. 2014. Available from: http://www.ias.org.uk/What-we-do/Publication-archive/Alcohol-Alert/October-2014/Applying-a-minimum-price-to-alcohol.aspx

60. Kisely S, Lawrence D. A time series analysis of alcohol-related presentations to emergency departments in Queensland following the increase in alcopops tax. J Epidemiol Community Health. 2015 Sep 16;jech-2015-205666.

61. Ludbrook A. Purchasing Patterns for Low Price Off Sales Alcohol: Evidence from the Expenditure and Food Survey [Internet]. Available from: http://www.shaap.org.uk/images/UserFiles/File/Reports%20and%20Briefings/Briefing%20-%20Purchase%20of%20low-price%20alcohol%20analysis.pdf

62. Ornstein SI, Levy D. Price and Income Elasticities of Demand for Alcoholic Beverages. In: Galanter M, Begleiter H, Cicero T, Deitrich R, Goodwin DW, Gottheil E, et al., editors. Genetics Behavioral Treatment Social Mediators and Prevention Current Concepts in Diagnosis [Internet]. Boston, MA: Springer US; 1983. p. 303–45. Available from: http://dx.doi.org/10.1007/978-1-4613-3617-4_18

63. Rabinovich L, Brutscher P-B, de Vries H, Tiessen J, Clift J, Reding A. The affordability of alcoholic beverages in the European Union. 2012;

64. Radaev V. Impact of a New Alcohol Policy on Homemade Alcohol Consumption and Sales in Russia. Alcohol Alcohol. 2015 May 1;50(3):365–72.

65. Robinson M, McCartney G, Beeston C. What is convincing evidence on alcohol pricing? BMJ. 2013 Aug 20;347:f5102.

66. Snowdon C. Chapter 10. Minimum unit pricing. In: Flaws and Ceilings: Price controls and the damage they cause [Internet]. [cited 2015 Nov 9]. p. 177–97. Available from:

For peer review only

For peer review only - http://bmjopen.bmj.com/site/about/guidelines.xhtml

http://www.iea.org.uk/publications/research/flaws-and-ceilings-price-controls-and-the-damage-they-cause

67. Wine and Spirits Trade Association. New claims that increasing the cost of alcohol saves lives are misleading [Internet]. Available from: http://www.wsta.co.uk/press/634-new-claims-that-increasing-the-cost-of-alcohol-saves-lives-are-misleading

68. World Health Organization. European status report on alcohol and health 2014. Pricing policies [Internet]. World Health Organization; 2014. Available from: http://www.euro.who.int/__data/assets/pdf_file/0003/244902/Pricing-policies.pdf

For peer review only

For peer review only - http://bmjopen.bmj.com/site/about/guidelines.xhtml

PRISMA 2009 Checklist

Section/topic	#	Checklist item	Reported on page #
**TITLE**			
Title	1	Identify the report as a systematic review, meta-analysis, or both.	1
**ABSTRACT**			
Structured summary	2	Provide a structured summary including, as applicable: background; objectives; data sources; study eligibility criteria, participants, and interventions; study appraisal and synthesis methods; results; limitations; conclusions and implications of key findings; systematic review registration number.	2
**INTRODUCTION**			
Rationale	3	Describe the rationale for the review in the context of what is already known.	3
Objectives	4	Provide an explicit statement of questions being addressed with reference to participants, interventions, comparisons, outcomes, and study design (PICOS).	3-4
**METHODS**			
Protocol and registration	5	Indicate if a review protocol exists, if and where it can be accessed (e.g., Web address), and, if available, provide registration information including registration number.	no published protocol
Eligibility criteria	6	Specify study characteristics (e.g., PICOS, length of follow-up) and report characteristics (e.g., years considered, language, publication status) used as criteria for eligibility, giving rationale.	3-4
Information sources	7	Describe all information sources (e.g., databases with dates of coverage, contact with study authors to identify additional studies) in the search and date last searched.	3
Search	8	Present full electronic search strategy for at least one database, including any limits used, such that it could be repeated.	3
Study selection	9	State the process for selecting studies (i.e., screening, eligibility, included in systematic review, and, if applicable, included in the meta-analysis).	3-4
Data collection process	10	Describe method of data extraction from reports (e.g., piloted forms, independently, in duplicate) and any processes for obtaining and confirming data from investigators.	4
Data items	11	List and define all variables for which data were sought (e.g., PICOS, funding sources) and any assumptions and simplifications made.	Tables 2+3
Risk of bias in individual studies	12	Describe methods used for assessing risk of bias of individual studies (including specification of whether this was done at the study or outcome level), and how this information is to be used in any data synthesis.	4
Summary measures	13	State the principal summary measures (e.g., risk ratio, difference in means).	Not stated, as all summary measures were included
Synthesis of results	14	Describe the methods of handling data and combining results of studies, if done, including measures of consistency (e.g., I^2) for each meta-analysis.	4, and Table 1

For peer review only

PRISMA 2009 Checklist

Section/topic	#	Checklist item	Reported on page #
Risk of bias across studies	15	Specify any assessment of risk of bias that may affect the cumulative evidence (e.g., publication bias, selective reporting within studies).	4
Additional analyses	16	Describe methods of additional analyses (e.g., sensitivity or subgroup analyses, meta-regression), if done, indicating which were pre-specified.	4
**RESULTS**			
Study selection	17	Give numbers of studies screened, assessed for eligibility, and included in the review, with reasons for exclusions at each stage, ideally with a flow diagram.	Figure 1 and online supplementary file
Study characteristics	18	For each study, present characteristics for which data were extracted (e.g., study size, PICOS, follow-up period) and provide the citations.	Tables 2+3
Risk of bias within studies	19	Present data on risk of bias of each study and, if available, any outcome level assessment (see item 12).	Tables 2+3
Results of individual studies	20	For all outcomes considered (benefits or harms), present, for each study: (a) simple summary data for each intervention group (b) effect estimates and confidence intervals, ideally with a forest plot.	Tables 2+3, p18-20
Synthesis of results	21	Present results of each meta-analysis done, including confidence intervals and measures of consistency.	No meta-analysis, discussed p18-20
Risk of bias across studies	22	Present results of any assessment of risk of bias across studies (see Item 15).	discussed p21
Additional analysis	23	Give results of additional analyses, if done (e.g., sensitivity or subgroup analyses, meta-regression [see Item 16]).	18-20
**DISCUSSION**			
Summary of evidence	24	Summarize the main findings including the strength of evidence for each main outcome; consider their relevance to key groups (e.g., healthcare providers, users, and policy makers).	20-21
Limitations	25	Discuss limitations at study and outcome level (e.g., risk of bias), and at review-level (e.g., incomplete retrieval of identified research, reporting bias).	20-21
Conclusions	26	Provide a general interpretation of the results in the context of other evidence, and implications for future research.	20
**FUNDING**			
Funding	27	Describe sources of funding for the systematic review and other support (e.g., supply of data); role of funders for the systematic review.	22

From: Moher D, Liberati A, Tetzlaff J, Altman DG, The PRISMA Group (2009). Preferred Reporting Items for Systematic Reviews and Meta-Analyses: The PRISMA Statement. PLoS Med 6(7): e1000097. doi:10.1371/journal.pmed1000097

For more information, visit: **www.prisma-statement.org**.

For peer review only - http://bmjopen.bmj.com/site/about/guidelines.xhtml

BMJ Open

Evidence for the effectiveness of minimum pricing of alcohol: a systematic review and assessment using the Bradford Hill criteria for causality

Journal:	*BMJ Open*
Manuscript ID	bmjopen-2016-013497.R2
Article Type:	Research
Date Submitted by the Author:	28-Feb-2017
Complete List of Authors:	Boniface, Sadie; King's College London, National Addiction Centre, Institute of Psychiatry, Psychology and Neuroscience<br>Scannell, Jack; University of Edinburgh, Innogen Institute, School of Social and Political Sciences<br>Marlow, Sally; King's College London, National Addiction Centre, Institute of Psychiatry, Psychology and Neuroscience
**Primary Subject Heading**:	Public health
Secondary Subject Heading:	Addiction
Keywords:	alcohol, policy, minimum unit pricing, PUBLIC HEALTH, Bradford Hill

SCHOLARONE™
Manuscripts

For peer review only

Evidence for the effectiveness of minimum pricing of alcohol: a systematic review and assessment using the Bradford Hill criteria for causality

Sadie Boniface,[1] Jack W Scannell,[2] Sally Marlow[1]

[1]National Addiction Centre, Institute of Psychiatry, Psychology and Neuroscience, King's College London, London, UK

[2]School of Social and Political Science, University of Edinburgh, Edinburgh, UK

Corresponding author: Dr Sadie Boniface, National Addiction Centre, Addiction Sciences Building (PO 48) Institute of Psychiatry, Psychology and Neuroscience, 16 De Crespigny Park, King's College London, SE5 8AF. sadie.boniface@kcl.ac.uk 020 7848 5097

Keywords: alcohol, policy, minimum unit pricing, public health, Bradford Hill

Word count: 3,432 (excluding title page, abstract, references, figures and tables)

For peer review only

ABSTRACT

Objectives: To assess the evidence for price-based alcohol policy interventions to determine whether minimum unit pricing is likely to be effective.

Design: Systematic review and assessment of studies according to PRISMA guidelines, against the Bradford Hill criteria for causality. Three electronic databases were searched from inception to February 2017. Additional articles were found through hand searching and grey literature searches.

Criteria for selecting studies: We included any study design that reported on the effect of price-based interventions on alcohol consumption or alcohol-related morbidity, mortality, and wider harms. Studies reporting on the effects of taxation or affordability, and studies that only investigated price elasticity of demand were beyond the scope of this review. Studies with any conflict of interest were excluded. All studies were appraised for methodological quality.

Results: Of 517 studies assessed, 33 studies were included: 26 peer-reviewed research studies and 7 from the grey literature. All nine of the Bradford Hill criteria were met, although different types of study satisfied different criteria. For example, modelling studies complied with the consistency and specificity criteria, time series analyses demonstrated the temporality and experiment criteria, and the analogy criterion was fulfilled by comparing the findings with the wider literature on taxation and affordability.

Conclusions: Overall, the Bradford-Hill criteria for causality were satisfied. There was very little evidence that minimum alcohol prices are not associated with consumption or subsequent harms. However the overall quality of the evidence was variable, a large proportion of the evidence base has been produced by a small number of research teams, and the quantitative uncertainty in many estimates or forecasts is poorly communicated in the literature. None the less, price-based alcohol policy interventions such as minimum unit pricing are likely to reduce alcohol consumption, alcohol-related morbidity and mortality.

ARTICLE SUMMARY

Strengths and limitations of this study

- This review adds to an emerging literature of systematic reviews synthesising findings using the Bradford Hill criteria for causality in research areas where traditional meta-analyses of randomised controlled trials are not possible or appropriate
- A range of study designs were included, allowing for a comprehensive review of a disparate evidence base to investigate whether minimum unit pricing of alcohol is likely to reduce alcohol consumption and alcohol-related harm
- Studies examining the effects of alcohol taxation or changes in alcohol affordability, or studies solely reporting on price elasticity of demand, were not included
- Methodological quality of studies was variable

For peer review only

INTRODUCTION

There are many policies and programmes that aim to reduce harms from alcohol (1). One of these is minimum alcohol pricing, which exists in a number of countries around the world. The most notable example of this is Canada, where there are government monopolies on alcohol sales and a variety of types of minimum pricing in operation. For example there is a minimum price per litre of a particular drink in British Columbia (2) and a (higher) minimum price linked to drink type and strength in Saskatchewan (3). Other countries with minimum alcohol pricing include Belarus, Kyrgyzstan, the Republic of Moldova, the Russian Federation and Ukraine (4). Minimum alcohol pricing is being considered by governments in the Republic of Ireland (5) and has also been reviewed in Australia (6) and New Zealand (7).

The situation with regards to minimum alcohol pricing in the United Kingdom is complex. In England and Wales there has been a ban on alcohol being sold at below cost (the total amount of 'duty plus VAT') since May 2014 (8); and the first conviction for selling alcohol below this level took place in 2016 (9). Duty plus VAT is equivalent to a 70cl bottle of vodka (37.5% ABV) costing a minimum of £8.72 (10), whereas under a minimum price of 50 pence per unit (one UK unit = 10ml or 8g ethanol) this would cost £13.13. In 2012 the UK coalition Government cited support for minimum unit pricing (MUP) in its alcohol strategy (11), and legislation to have a minimum price of £0.50 per unit was passed in Scotland the same year (4). Following the change to a Conservative majority Government in 2015, it is unclear whether there is still central Government support for MUP. In Scotland, the Scotch Whisky Association challenged the 2012 legislation in the Scottish Court of Session, which referred the case to the Court of Justice of the European Union (CJEU) in 2014 (12). In late 2015, the CJEU referred the case back to the Scottish courts to investigate proportionality (that the same objective cannot be met through increased taxation) (13), which could have implications for other EU countries considering MUP. In late 2016, the Scottish Court of Session ruled that MUP does not contravene EU law (14), however the Scotch Whisky Association then appealed to the UK Supreme Court (15).

In light of this ongoing consideration of MUP in the United Kingdom, in this paper we assess the effectiveness of minimum alcohol price interventions to reduce alcohol-related harm. Alcohol-related harm costs the NHS in England £3.5bn each year and the estimated cost to society is £21 billion per year (16). The latest annual figures for England (population of 54 million) show over one million alcohol-related hospital admissions (2013/14) and six and a half thousand alcohol-related deaths (2013); and these figures represent increases compared with a decade previously of 115% and 10% respectively (16).

We systematically review the literature on the effect of price interventions or policies such as MUP on alcohol consumption, alcohol-related morbidity and mortality, and wider harms. We use the nine Bradford Hill criteria for causality as a framework with the aim of assessing the likely effectiveness of MUP as a policy to reduce alcohol consumption and alcohol-related harm.

METHODS

A systematic literature search was performed according to Preferred Reporting Items for Systematic Reviews and Meta-Analyses (PRISMA) guidance (see Figure 1 for PRISMA flow diagram and online supplementary file for excluded studies).

For peer review only

[figure 1 to go here]

Identification of studies

Three electronic databases were searched for titles or abstracts containing ‘"minimum unit pric$" OR "minimum pric$"’ OR “floor pric$” OR “pric$ AND policy” AND alcohol. The databases were PsycINFO (1806 to February Week 2 2017), Embase (1974 to 2017 Week 07), Ovid Medline (1946 to February Week 2 2017). We also searched the websites of five alcohol charities for publications or reports related to “price”, and also searched 20 leading UK think tanks for “alcohol” or “addiction”.

Inclusion criteria were: any study design; population level studies exploring at least one aspect of the effect of interventions or policies leading to changes in the minimum price of alcohol, including but not limited to changes in alcohol sales, consumption, morbidity and mortality; individual level studies exploring minimum alcohol prices, and alcohol purchasing, consumption, morbidity and mortality; written in English.

Exclusion criteria were: studies about taxation, affordability, price elasticity of demand for alcohol, and general changes in alcohol price not the result of an intervention or policy (there is a large literature on each of these already and reviewing all of these studies was beyond the scope of this review); studies about public perceptions of MUP; studies where a conflict of interest was reported in the paper, whether this was in favour of or against MUP.

All 33 studies that met the inclusion criteria were assessed against the Bradford Hill criteria for causality and the methodological quality appraised. These included 26 original research studies and 7 studies from the grey literature, and in addition two systematic reviews pertinent to the analogy criterion were included. Of the 26 research studies, there were 9 cross sectional surveys, 8 time series analyses or similar, 7 modelling studies, one qualitative study, and one trial.

Analysis of included studies

Quality of included studies was assessed independently by two reviewers and using validated tools. Due to the wide variation in study designs among the included studies, the Effective Public Health Practice Project’s (EPHPP) tool was used for assessing all quantitative studies, as recommended by the Cochrane Handbook for assessing studies in public health (17). Qualitative studies (n=1) and systematic reviews (n=2) included in this review were not covered by the EPHPP tool and so were assessed using the Critical Appraisal Skills Programme (CASP) tools specific to these study designs.

Nine criteria in order to determine causality were suggested by Bradford Hill in an influential 1965 paper (18). Increasingly, the Bradford Hill criteria are a standard framework to assess the impact of interventions where it is not ethical or practical to conduct randomised controlled trials. Our interpretation of the Bradford Hill criteria for the purpose of this review is listed in Table 1. Two reviewers assessed each study against each of the nine criteria and agreed which studies provided relevant evidence for or against each criterion.

For peer review only

Table 1: Bradford Hill criteria for assessing causation and the definitions used in this review

Criterion	Bradford Hill criteria (1965)	Application in this review
1. Strength of the association	The strength of a supposed association between an intervention and an outcome is determined by the appropriate statistic used to measure the protective effect of an intervention (e.g. relative risk or odds ratio). This is the most important factor determining causation	A statistically significant change (P<0.05) in alcohol consumption or alcohol related harms, in the expected direction. The exact magnitude of the association was assessed on a study by study basis
2. Consistency	Has it been repeatedly observed by different persons, in different places, circumstances and times?	Whether different studies conducted in different locations, in different populations, by different investigators and at different times have reported similar findings
3. Specificity	Specificity is present when the intervention is exclusive to the outcome and when the outcome has no other known cause or associated risk factors; cautions that this criterion should not be overemphasized and that if specificity is not apparent this does not preclude causation	If pricing was the only reason that alcohol consumption or alcohol-related harm could have fallen, this adds to the argument for causality. However if a price intervention was one of a number of alcohol policy interventions, then this criterion is not satisfied
4. Temporality	Refers to temporal relationship of association between exposure and disease outcome; to infer causality, exposure must precede outcome	The pricing intervention studied must have taken place before a change in alcohol consumption or harm was observed
5. Dose-response	If the association is one in which a dose-response curve or biological gradient can be observed, this adds to the case for causality	If interventions leading to a larger increase in prices had a greater effect on alcohol consumption and alcohol related harm than interventions where the price change was small, or if studies demonstrate that different minimum prices have differing effects, in the expected direction
6. Plausibility	A likely biological mechanism linking the intervention to the observed findings helps to explain causality, plausibility depends on biological knowledge of the day	Studies that found an association between price and population-level alcohol consumption and that heavier drinkers tend to purchase the cheapest alcohol could demonstrate plausibility
7. Coherence	When the evidence from different disciplines sources "hangs well together" and does not conflict with other generally known facts, this criterion is met	Describes whether studies conducted in different settings or disciplines had complementary findings. Will not be demonstrated by a single study in isolation but rather the evidence base as a whole
8. Experiment	Experimental evidence from laboratory studies or RCTs could potentially provide strongest support for causation	In addition to laboratory studies and RCTs, natural experiments with before-and-after measures could also show the effectiveness

For peer review only

	This criterion often provides the strongest support for causation and describes whether there is empirical evidence for the association	of minimum unit pricing in a 'real world' setting
9. Analogy	Causality is supported by analogy if there are similar associations or causal relationships in other areas of relevance, weakest form of evidence of causality	Other areas of relevance include whether higher taxation on alcohol is associated with reduced alcohol consumption and alcohol related harm, and may require drawing on additional literature outside of the main systematic review

For peer review only

RESULTS

The included studies that are published in peer-reviewed journals (26 research studies and two systematic reviews) are listed by study type in Table 2 with information on study characteristics and methodological quality. Of the research studies, the methodological quality was rated as 'strong' in 15 studies, 'moderate' in 8 studies, and 'weak' in 3 studies. Both of the systematic reviews were rated 'strong'. The seven reports from the grey literature are listed in Table 3. Five of the seven were rated as of 'strong' methodological quality, with the remaining two not appropriate to rate using our critical appraisal tool.

For peer review only

Table 2: Studies published in peer-reviewed journals included in Bradford Hill criteria assessment

Study type	First author and year published	Study characteristics					Study assessment			Bradford Hill criteria met*
		Country	Study design	Population or participants	Pricing intervention studied	Outcomes studied	Peer-reviewed	Conflict of interest	Quality rating	
Natural experiments and time series analyses	Bhattacharya 2013 (19)	Russia	Time series analysis of panel data set	Populations of 77 Russian oblasts (provinces), 1970-2000	Substantial increases in administratively-set alcohol prices 1985-1988, along with 6 other anti-alcohol measures	Mortality	Yes	Not stated	Strong	SA, CON, TE, PL, CO, EX,
	Herttua 2015 (20)	Finland	Time series analysis	General population using population registry	Modelled 1% increase in the average minimum price of all alcoholic beverages based on actual price increases adjusted for inflation using Consumer Price Index	Alcohol related mortality	Yes	None	Strong	SA (not universal findings – subgroup only), CON (counter findings) TE, PL, CO, EX
	Stockwell 2012 (2)	Canada	Cross-section versus time series analysis of ecological data	Population of British Columbia	Actual minimum price increased over a 20 year period. Study modelled a 10% increase in the average minimum price of all alcoholic beverages adjusted by monthly	Alcohol consumption (measured by sales)	Yes	None	Strong	SA, CON, TE, DR, CO, EX

For peer review only - http://bmjopen.bmj.com/site/about/guidelines.xhtml

Study type	First author and year published	Study characteristics					Study assessment			Bradford Hill criteria met*
		Country	Study design	Population or participants	Pricing intervention studied	Outcomes studied	Peer-reviewed	Conflict of interest	Quality rating	
					Consumer Price Index adjusted by monthly Consumer Price Index					
	Stockwell 2012 (3)	Canada	Cross-section versus time series analysis of ecological data	Population of Saskatchewan	Actual minimum price increased over a 7 year period. Study modelled a 10% increase in the average minimum price of all alcoholic beverages adjusted by monthly Consumer Price Index adjusted by monthly Consumer Price Index	Alcohol consumption (measured by sales)	Yes	Not stated	Strong	SA, CON, TE, DR, CO, EX
	Stockwell 2013 (21)	Canada	Cross-section versus time series analysis of ecological data	Populations of 89 geographic areas in British Columbia	Actual minimum price increased over a 20 year period. Study modelled 10% increase in the average minimum price of all alcoholic beverages adjusted by monthly Consumer Price Index	Alcohol-attributable hospital admissions	Yes	Not stated	Strong	SA, CON, TE, DR, PL, CO, EX
	Treisman 2010 (22)	Russia	Secondary analysis of historical data with focus on price	Population of Russia	Price liberalisation of vodka in early 1990s - in 1993 real price of vodka was around 25% of that in 1990	Mortality	Yes	Not stated	Strong	SA, CON, TE, PL, CO, EX

For peer review only - http://bmjopen.bmj.com/site/about/guidelines.xhtml

Study type	First author and year published	Study characteristics					Study assessment			Bradford Hill criteria met*
		Country	Study design	Population or participants	Pricing intervention studied	Outcomes studied	Peer-reviewed	Conflict of interest	Quality rating	
			changes 1990-1994							
	Wald 1984 (23)	Poland	Analysis of routine data 1970-1981	Population of Poland	Poor harvest led to high prices, rationing and illegal sales	Alcohol consumption and alcohol-related hospital admissions	Yes	Not stated	Weak	CON, TE, PL, CO, EX
	Zhao 2013 (24)	Canada	Cross-section versus time series analysis of ecological data	Populations of 16 Health Service Delivery Areas in British Columbia, Canada	Actual minimum price increased over a 20 year period. Study modelled 10% increase in the average minimum price of all alcoholic beverages adjusted by monthly Consumer Price Index. Also looked at outlet density	Acute, chronic and wholly alcohol attributable mortality	Yes	None	Strong	SA, CON, TE, DR, PL, CO, EX
Modelling studies	Brennan 2014 (25)	England	Modelling study using SAPM	UK national surveys of general population (subgroups of moderate, harmful, hazardous)	MUP of £0.40, £0.45 and £0.50. Ban on below cost selling	Alcohol consumption, consumer spending, 47 health harms, QALYs	Yes	None	Strong	CON, SP, DR, PL, CO
	Holmes 2014 (26)	England	Modelling study using	UK national surveys of	MUP of 45p	Alcohol consumption,	Yes	None	Strong	CON, SP, PL,

For peer review only - http://bmjopen.bmj.com/site/about/guidelines.xhtml

Study type	First author and year published	Study characteristics					Study assessment			Bradford Hill criteria met*
		Country	Study design	Population or participants	Pricing intervention studied	Outcomes studied	Peer-reviewed	Conflict of interest	Quality rating	
			SAPM	general population (subgroups of moderate, harmful, hazardous)		consumer spending, 47 health harms, QALYs				CO
	Meier 2009 (27)	UK	Modelling study using SAPM	UK national surveys of general population (subgroups of moderate, harmful, hazardous)	10 pricing policy options, including different levels of MUP (of 33 analysed)	Alcohol consumption, consumer spending, 47 health harms, crime, employment	Yes	None	Strong	CON, SP, DR, PL, CO
	Meier 2016 (28)	England	Modelling study using SAPM	UK national surveys of general population (subgroups of moderate, increasing risk, heavy)	MUP of £0.50 compared with three alcohol taxation interventions	Alcohol consumption in different income and socio-economic groups	Yes	None	Strong	CON, SP, PL, CO
	Purshouse 2010 (29)	England	Modelling study using SAPM	UK national surveys of general population (subgroups of moderate, harmful,	18 different pricing policies (including MUP)	Alcohol consumption, consumer spending, 47 health harms, QALYs	Yes	None	Strong	CON, SP, DR, PL, CO

For peer review only - http://bmjopen.bmj.com/site/about/guidelines.xhtml

Study type	First author and year published	Study characteristics					Study assessment			Bradford Hill criteria met*
		Country	Study design	Population or participants	Pricing intervention studied	Outcomes studied	Peer-reviewed	Conflict of interest	Quality rating	
				hazardous)						
	Sharma 2016 (30)	Australia	Counterfactual analysis	Representative sample of households (n=884) completing 12-month Homescan shopping survey	MUP of A$2	Alcohol purchasing and consumption	Yes	None	Strong	CON, SP, PL, CO
	Vandenberg 2016 (31)	Australia	Modelling study	Representative sample of households (n=885) completing Homescan shopping survey	MUP of A$1 compared with a specific alcohol tax	Alcohol purchasing and consumption	Yes	None	Strong	CON, SP, PL, CO
Cross-sectional studies	Black 2011(32)	Scotland	Cross-sectional survey	377 hospital patients with serious alcohol problems	UK alcohol units purchased below proposed MUP of £0.40p/£0.50p	Alcohol consumption	Yes	None	Moderate	SA CON, DR, PL, CO
	Callinan 2015 (33)	Australia	Cross-sectional survey	Drinkers 18+ participating in Australian International Alcohol	Australian standard drinks purchased below proposed minimum prices of A$0.80/A$1.00/A$1.25	Alcohol consumption	Yes	Not stated	Moderate	SA, CON, DR, PL, CO

For peer review only - http://bmjopen.bmj.com/site/about/guidelines.xhtml

Study type	First author and year published	Study characteristics					Study assessment			Bradford Hill criteria met*
		Country	Study design	Population or participants	Pricing intervention studied	Outcomes studied	Peer-reviewed	Conflict of interest	Quality rating	
				Control study (n=1,681)						
	Cousins 2016 (34)	Ireland	Cross-sectional survey	3,187 adults in 2013 National Alcohol Diary Survey	Alcohol units purchased below proposed minimum price of €1.00	AUDIT-C score	Yes	None	Strong	SA, CON, PL, CO
	Crawford 2012 (35)	England	Cross-sectional survey	515 members of the public	UK alcohol units purchased below proposed MUP of £0.50	AUDIT score	Yes	None	Moderate	SA, CON, PL, CO
	Falkner 2015 (36)	New Zealand	Cross-sectional survey	115 adults undergoing alcohol detoxification	New Zealand standard drinks purchased below proposed minimum prices of NZ$1.00/NZ$1.10/NZ$1.20	Alcohol consumption	Yes	No	Moderate	SA, CON, PL, CO
	Forsyth 2014 (37)	Scotland	Cross-sectional survey	Shopkeepers of 144 off licences in Glasgow	MUP of £0.50	Products affected, and hospital admissions	Yes	None	Weak	CON, PL (weakly), CO
	Ludbrook 2012 (38)	UK	Cross-sectional survey	Expenditure and Food Survey data from 2006-8 (n=18,624)	Purchasers of alcohol less than £0.45 per unit	Income of purchasers of cheap alcohol	Yes	Not stated	Moderate	SA, CON, PL, CO
	Sharma 2014 (39)	Australia	Cross-sectional survey	Representative sample of households	MUP of A$1, and taxation	Alcohol consumption (measured by	Yes	None	Moderate	SA, CON, DR, PL,

For peer review only - http://bmjopen.bmj.com/site/about/guidelines.xhtml

Study type	First author and year published	Study characteristics					Study assessment			Bradford Hill criteria met*
		Country	Study design	Population or participants	Pricing intervention studied	Outcomes studied	Peer-reviewed	Conflict of interest	Quality rating	
				(n=885) completing shopping survey		projected sales)				CO
	Sheron 2014 (40)	UK	Cross-sectional survey	Adult patients in a liver unit of a hospital (n=204)	UK alcohol units purchased below £0.50	Alcohol consumption	Yes	Not stated	Moderate	SA, CON, DR, PL, CO
Intervention studies	Babor 1978 (41)	USA	Trial (not randomised)	34 male volunteers in live-in research facility	'Happy Hour' with a reduction in set price of alcohol for one group of participants	Alcohol consumption	Yes	Not stated	Weak	SA, CON, SP, TE, CO, EX
Qualitative studies	Seaman 2013 (42)	Scotland	Qualitative study	130 participants aged 16-30	Hypothetical minimum price increases	Alcohol consumption and substitution with other substances	Yes	None	Moderate	CON, CO
Systematic reviews	Wagenaar 2009 (43)	Worldwide	Systematic review and meta-analysis	Studies tended to cover general population	Alcohol price and taxation interventions studied together	Alcohol consumption (measured by alcohol sales or self-reported consumption)	Yes	None	Strong	AN
	Wagenaar 2010 (44)	Worldwide	Systematic review and	Studies tended to	Alcohol price and taxation interventions	Alcohol-related	Yes	Not stated	Strong	AN

For peer review only - http://bmjopen.bmj.com/site/about/guidelines.xhtml

Study type	First author and year published	Study characteristics					Study assessment			Bradford Hill criteria met*
		Country	Study design	Population or participants	Pricing intervention studied	Outcomes studied	Peer-reviewed	Conflict of interest	Quality rating	
			meta-analysis	cover general population	studied together	morbidity (disease, injury, suicide, traffic crashes, sexually transmitted diseases, other drug use, crime and misbehaviour) and mortality				

*Abbreviations used for the Bradford Hill criteria: strength of the association (SA), consistency (CON), specificity (SP), temporality (TE), dose-response (DR), plausibility (PL), coherence (CO), experiment (EX), analogy (AN)

For peer review only - http://bmjopen.bmj.com/site/about/guidelines.xhtml

Table 3: Studies published in the grey literature included in Bradford Hill criteria assessment

	Study characteristics					Study assessment			Bradford Hill criteria met*
**Author and year published**	**Country**	**Study design**	**Population or participants**	**Pricing intervention studied**	**Outcomes studied**	**Peer-reviewed?**	**Conflict of interest?**	**Quality rating**	
Angus 2016 (45)	Scotland	Modelling study using SAPM	Scottish general population survey (subgroups of moderate, harmful, hazardous)	MUP of 30p, 40p, 50p, 60p and 70p, compared with taxation interventions	Alcohol consumption, consumer spending, exchequer and retail revenue, 47 health harms	Not stated	None	Strong	CON, SP, DR, PL, CO
Booth 2008 (46)	Worldwide	Review of reviews and systematic review	Studies tended to cover general population	Various minimum unit prices and taxation interventions	Alcohol consumption and various measures of alcohol harm	Yes	None	Strong	AN
Brennan 2008 (47)	England	Modelling study using SAPM	Adults in England	General price increases. MUP of £0.20, £0.25, £0.30, £0.35, £0.40, £0.45, £0.50, £0.60 and £0.70. Restrictions on off-trade price promotions.	Alcohol consumption, consumer spending, sales duty and VAT, 47 health harms, crime, and employment	Not stated	None	Strong	CON, SP, DR, PL, CO
Hill McManus 2012 (48)	Canada	Modelling study using SAPM	Adults in two Canadian provinces (Ontario and British Columbia)	MUP of C$1.50	Alcohol consumption, consumer spending, hospital admissions, mortality, crime	No	None	Strong	CON, SP, PL, CO
Institute for Fiscal Studies	Great Britain	Economic modelling study	Shopping data from 25,248 British households	MUP of £0.45	Alcohol consumption	Not stated	Not stated	Not possible to rate	CON, SP, CO

For peer review only - http://bmjopen.bmj.com/site/about/guidelines.xhtml

	Study characteristics					Study assessment			Bradford Hill criteria met*
**Author and year published**	**Country**	**Study design**	**Population or participants**	**Pricing intervention studied**	**Outcomes studied**	**Peer-reviewed?**	**Conflict of interest?**	**Quality rating**	
2010 (49)		using market research data							
Institute for Fiscal Studies 2013 (50)	Great Britain	Economic analysis	Population of Great Britain	MUP of £0.45 and increased alcohol taxation	Alcohol consumption	Not stated	Not stated	Not possible to rate	CON, SP, CO
Meng 2010 (51)	Scotland	Modelling study using SAPM	Adults in Scotland	MUP of £0.20, £0.25, £0.30, £0.35, £0.40, £0.45, £0.50, £0.60 and £0.70. Restrictions on off-trade price promotions.	Alcohol consumption, consumer spending, 47 health harms, crime, employment	Not stated	None	Strong	CON, SP, DR, PL, CO

*Abbreviations used for the Bradford Hill criteria: strength of the association (SA), consistency (CON), specificity (SP), temporality (TE), dose-response (DR), plausibility (PL), coherence (CO), experiment (EX), analogy (AN)

For peer review only - http://bmjopen.bmj.com/site/about/guidelines.xhtml

Consideration against the Bradford Hill criteria for determining causality

1. Strength of the association

In 16/26 studies published in peer-reviewed journals, strength of the association between pricing and alcohol consumption or alcohol related harm was evidenced by a summary statistic such as an odds ratio, and by a test of the statistical significance of the association. As well as the statistical significance of the summary statistic, the magnitude of the effect was also considered, as a larger effect size corresponds to a greater population health impact. Studies in Canada found that 10% increases in minimum prices were associated with reductions in alcohol consumption of 3.4-8.4% (2,3), reductions in alcohol attributable hospital admissions of 9% (21), and reductions in alcohol-related mortality of 32%, each of which was statistically significant (24). Cross-sectional studies in the UK, Ireland, Australia (32–36,38–40) and one trial from the USA (41) found statistically significant associations between cheaper alcohol and heavier drinking. The magnitude of the association varied across these studies but due to different study measures and outcomes the results are not all comparable. As an indication, the odds ratio (OR) for buying alcohol below a proposed minimum price among heavier drinkers was 1.34 in Crawford's study (35), 1.50 in Cousins's study (34) and1.70 in Callinan's study (33). There was not any evidence to support this criterion from the grey literature. Overall there is reasonably good support for the strength of the association criterion.

2. Consistency

This criterion requires looking across all the studies included in the review to see whether similar conclusions have been drawn. Inverse associations between alcohol pricing and alcohol consumption or harm have been documented in countries in Europe, North America and Australia, and although most studies are from the last 10 years, there are studies from the 1970s and 1980s as well. There is evidence from different research teams and different types of study including cross-sectional studies, time series analyses and econometric modelling studies. Support for the consistency criterion is very strong.

3. Specificity

The specificity criterion relates to whether changes in alcohol consumption or harm could be attributed to anything other than the price intervention. Many studies included have statistically adjusted for confounding factors, however the best support for the specificity criterion comes from the econometric modelling studies because there is no risk of residual confounding. The Sheffield Alcohol Policy Model is one such model and has been applied in England (25–29,47), Scotland (45,51) and Canada (48) and provides very strong support for the specificity criterion. Further support is provided by other different modelling studies in the UK (49,50) and Australia (30,31) and a (non-randomised) trial in the US (41). Thus support for the specificity criteria is very strong.

4. Temporality

It is important that pricing interventions take place before changes to alcohol consumption and harm in order to attribute causality. Strong support for this criterion comes from research following the introduction of MUP in Canada, where minimum price increases preceded reductions in alcohol consumption (2,3), alcohol attributable hospital admissions (21), and alcohol-related mortality (24). Studies where price changes preceded the expected changes in alcohol consumption or harm have

For peer review only - http://bmjopen.bmj.com/site/about/guidelines.xhtml

also been conducted in Russia (19,22), Poland (23), and Finland (20). Overall there is very strong support for the temporality criterion.

5. Dose-response/biological gradient

This criterion is supported if different price levels have been found to have differing effects on consumption or harm. Many of the studies using the Sheffield Alcohol Policy Model explore the impact of a range of potential MUP options (25,27,29,45,51), and these consistently suggest that the higher the MUP, the greater the reductions in alcohol consumption or alcohol-related harms. The Canadian studies of minimum pricing lend further support for this criterion because the analysis presents the effect on consumption or harm of a modelled 1% increase in price, meaning dose-response can be inferred (2,3,21,24). Dose response is supported to a lesser extent by evidence from cross-sectional studies that heavier drinkers are more likely to pay less than a proposed MUP (32–34,39,40). Overall, there is strong support for the dose-response criterion, although the relationship is difficult to quantify.

6. Plausibility

This criterion refers to whether there is evidence that alcohol price can be used as an economic mechanism to influence consumption at a population level, and whether heavy drinkers tend to purchase cheaper alcohol. There is evidence from 21/26 research studies 4/7 studies in the grey literature that the price of alcohol is inversely related to alcohol-related morbidity, hospital admissions, or mortality. Moreover, there is also evidence from numerous cross-sectional studies in the UK, Ireland and Australia (32–36,38–40) and one trial from the USA (41) that heavier drinking was significantly associated with purchasing alcohol below specified prices, further suggesting that economic mechanisms such as minimum pricing would particularly affect the heaviest drinkers. This provides strong support for the plausibility criterion.

7. Coherence

This criterion refers to whether studies from different disciplines have had complementary findings and whether these fit or 'hang' well together. It is different to consistency, which is more concerned with reproducibility of findings. The findings of the majority of studies supported the coherence criterion in that they suggest that real-world minimum unit pricing (e.g. (2,3,21,24)) or minimum price increases (e.g. (19,20,23)) led to reductions in alcohol consumption and alcohol-related harm and cross sectional surveys find that it is the heavier drinkers that are drinking the cheapest alcohol (e.g. (32,40)). The modelling studies which utilise survey data in turn suggest heavier drinkers will be most affected by MUP (e.g. (26)), Overall the evidence base provides strong support for this criterion.

8. Experiment

We have not identified any randomised controlled trials of minimum pricing or price-based interventions to reduce alcohol consumption. There is a small (and not randomised) trial from the 1970s (41) which found participants living in controlled conditions and offered a daily 'Happy Hour' discount drank significantly more alcohol than those who were not offered the discount. There is however substantial evidence in support of the experiment criterion from time series analyses or natural experiments, for example where minimum pricing was introduced in Canada (3,21,24) and

For peer review only - http://bmjopen.bmj.com/site/about/guidelines.xhtml

where prices fluctuated in the late 1980s and early 1990s in Russia (19,22), and to a lesser extent in Finland, where minimum price increases were associated with reduced mortality only among men with a basic education (20). These studies provide tentative support for the experiment criterion.

9. Analogy

To address the analogy criterion areas related to minimum alcohol pricing must be considered. There is evidence from literature on the affordability of alcohol (52) that consumption and harm are very responsive to the affordability of alcohol. Large systematic reviews have investigated the price elasticity of demand for alcohol (53), and have found that higher alcohol pricing and taxation (considered together) are associated with reductions in alcohol consumption, alcohol-related morbidity and mortality (43,44,46). Overall the support for the analogy criterion is very strong, although Bradford Hill describes this as the weakest evidence for causality.

DISCUSSION

We assessed 26 research studies and two systematic reviews, plus a further seven studies from the grey literature in this review of the evidence for priced based interventions – such as MUP - to reduce alcohol consumption and alcohol-related harm. All nine of the Bradford Hill criteria for causality were met and the vast majority of studies offered support for price-based alcohol policy interventions. However, the evidence for two of the criteria, although present, was not as strong as it was for the other criteria. These criteria were strength of the association (criterion 1) and experiment (criterion 8), and according to Bradford Hill these are the two criteria that can provide the strongest evidence for causality. Therefore although all of the criteria were supported, we conclude that it is highly probable, but not definite, that introducing MUP for alcohol would reduce alcohol consumption and alcohol-related harms. It is also of note that different types of study tended to satisfy different Bradford Hill criteria, and that different study designs also produced evidence of the effectiveness of minimum pricing in relation to different outcomes. This is summarised in Figure 2. This underlines the importance of including a variety of study designs in this review.

[figure 2 to go here]

Strengths of this study are that this is the first to have systematically reviewed the literature relevant specifically to alcohol minimum pricing policies. We had broad inclusion criteria with regards to study design, price intervention and outcome measure, allowing for a comprehensive review of the evidence base. Application of the Bradford Hill criteria as part of a narrative systematic literature review is a useful and emergent technique for identifying causality: a PubMed search for systematic reviews with Bradford Hill' mentioned in the title or abstract yielded 28 results, 90% of which were published in the last five years. The limitations of this systematic review relate mainly to the broad range of studies included. It was not possible to conduct any kind of meta-analysis and therefore we do not present a pooled estimate for the likely effect of MUP on certain outcomes. The exact effect of any MUP would be influenced by a range of factors, including: the minimum price level chosen, how broadly it is applied, how strongly it is enforced, and contextual factors such as affordability (in the UK alcohol was 54% more affordable in 2014 than it was in 1980 (16)), other governmental regulations and the price level pre-MUP. Occasionally, minimum pricing has been implemented as

For peer review only - http://bmjopen.bmj.com/site/about/guidelines.xhtml
For peer review only

part of a range of measures (e.g. (19)), and these studies were considered alongside studies where MUP was implemented in isolation. This emphasises the importance of the specificity criterion.

There were also challenges with the quality appraisal. The EPHPP quality assessment tool was used to assess quantitative studies and the majority of studies were rated as strong or moderate. However it was not possible to appraise two of the studies from the grey literature using this tool, and there were some challenges assessing the econometric modelling studies against this framework. However overall we think that our quality appraisal across the different studies is broadly comparable. It should also be noted that although a number of studies were rated as 'strong', this is in relation to their respective study designs and does not reflect the position of the study type in the hierarchy of evidence framework.

This is the first systematic review that has addressed the effectiveness of minimum alcohol price interventions such as MUP using the Bradford Hill criteria. It was beyond the scope of this review to study the impact of generalised increases in alcohol prices (as opposed to minimum prices). However where such studies have been done, a minimum price or floor price has been recommended, for example in Gruenewald's 2006 study in Sweden which found that the lowest quality (the cheapest) alcohol has the highest price elasticity (54). Previous systematic reviews of alcohol price and consumption (43) and alcohol-related harm (44) have tended to consider the effect of price increases and increased taxation together. These reviews found significant effects on consumption and morbidity and mortality. Although price regulation and taxation are closely related policy options, evidence from surveys (55) and modelling studies (45) suggests that the effects of each are different, although it is known that the majority of tax increases are passed on as increased prices for consumers (43,56). It was beyond the scope of this review to discuss whether MUP is regressive in detail, but as it only affects the prices of the cheapest drinks, which are usually consumed by the heaviest drinkers, MUP is likely to narrow health inequalities (28,31). A recent rapid evidence review published in The Lancet examined alcohol control policies in England and recommended a combination of MUP and tax increases to reduce alcohol harm and increase government revenue, rather than either in isolation (57). It is also important to highlight that a considerable proportion of included studies were produced by a small number of research teams. Also, with regards to the econometric modelling studies, uncertainty in estimates or forecasts is often poorly communicated outside of the academic literature. The overall risk of bias in the included studies was minimised by excluding studies with a conflict of interest (either for or against MUP). It was not possible to assess publication bias using an analytical technique such as a funnel plot due to the narrative nature of the review, however we anticipate that by including grey literature in this review we have mitigated publication bias as far as reasonably possible.

Overall the findings of this review lend strong support for policies such as MUP in reducing alcohol consumption and alcohol-related harm, with all nine of the Bradford Hill criteria met, and little by way of counter findings. As it is unlikely to be feasible to conduct randomised controlled trials (RCTs) of MUP, the decision whether or not to introduce MUP will not be informed by a systematic review and meta-analysis of RCTs, and therefore this synthesis of evidence according to the Bradford Hill criteria is of value.

Unanswered questions about the effectiveness of MUP remain; for example, this review has highlighted that support was moderate or tentative for two of the Bradford Hill criteria ('strength of

For peer review only - http://bmjopen.bmj.com/site/about/guidelines.xhtml

the association' and 'experiment' respectively). There may be opportunities to explore this in countries such as Scotland if MUP is implemented. If Scotland were to implement MUP, then it would be possible to evaluate the validity of the Sheffield Alcohol Policy Model studies conducted using Scottish data. It would also be possible to conduct a longitudinal study to evaluate the effectiveness of MUP in reducing alcohol consumption and alcohol-related morbidity and mortality. The findings of this natural experiment would have relevance elsewhere within and outside the UK.

ACKNOWLEDGEMENTS

We would like to thank Rebecca McDonald for advice on using the Bradford Hill criteria in a systematic review, Dr James Nicholls for advice on study interpretation, and Dr Daniel Stahl for statistical advice on some of the included studies.

CONTRIBUTIONS

SM conceived the idea. SB conducted the initial search. SM and SM contributed to independently reviewing abstracts, hand-searching reference lists, completing data extraction, and conducting quality appraisal. All authors contributed to the analysis and interpretation of the results and contributed to writing the manuscript. SB is guarantor.

FUNDING

This research received no specific grant from any funding agency in the public, commercial or not-for-profit sectors.

COMPETING INTERESTS

All authors have completed the ICMJE uniform disclosure form at www.icmje.org/coi_disclosure.pdf and declare: no support from any organisation for the submitted work; no financial relationships with any organisations that might have an interest in the submitted work in the previous three years; no other relationships or activities that could appear to have influenced the submitted work.

Two of the authors work at King's College London, which as an institution is listed as a member of the Alcohol Health Alliance. SM has received funding indirectly from UKCTAS, which as an institution is also listed as a member of the Alcohol Health Alliance. However none of the authors have any relationship with the Alcohol Health Alliance.

For peer review only

For peer review only - http://bmjopen.bmj.com/site/about/guidelines.xhtml

FIGURE LEGENDS

Figure 1: PRISMA 2009 flow diagram of studies in this systematic review

Figure 2: This model shows that different study types tended to produce evidence of effectiveness of minimum pricing in relation to different outcomes. Studies cited in the figure are key examples of the literature in that area and do not represent an exhaustive list.

REFERENCES

1. Anderson P, Chisholm D, Fuhr DC. Effectiveness and cost-effectiveness of policies and programmes to reduce the harm caused by alcohol. Lancet Lond Engl. 2009 Jun 27;373(9682):2234–46.

2. Stockwell T, Auld MC, Zhao J, Martin G. Does minimum pricing reduce alcohol consumption? The experience of a Canadian province. Addiction. 2012 May 1;107(5):912–20.

3. Stockwell T, Zhao J, Giesbrecht N, Macdonald S, Thomas G, Wettlaufer A. The Raising of Minimum Alcohol Prices in Saskatchewan, Canada: Impacts on Consumption and Implications for Public Health. Am J Public Health. 2012 Oct 18;102(12):e103–10.

4. World Health Organization. European status report on alcohol and health 2014. Pricing policies [Internet]. World Health Organization; 2014. Available from: http://www.euro.who.int/__data/assets/pdf_file/0003/244902/Pricing-policies.pdf

5. Department of Health (Ireland). The Public Health (Alcohol) Bill 2015 [Internet]. 2015 [cited 2017 Feb 17]. Available from: http://health.gov.ie/blog/publications/public-health-alcohol-bill-2015/

6. The Guardian. Minimum alcohol price not in the public interest, says health agency. The Guardian [Internet]. 2014 May 1 [cited 2017 Feb 17]; Available from: https://www.theguardian.com/world/2014/may/01/minimum-alcohol-price-not-in-the-public-interest-says-health-agency

7. New Zealand Ministry of Justice. Alcohol minimum pricing policies [Internet]. [cited 2017 Feb 17]. Available from: https://justice.govt.nz/justice-sector-policy/key-initiatives/sale-and-supply-of-alcohol/alcohol-minimum-pricing-report/

8. Home Office. Guidance on banning the sale of alcohol below the cost of duty plus VAT. For suppliers of alcohol and enforcement authorities in England and Wales [Internet]. 2015 [cited 2016 Jun 3]. Available from: https://www.gov.uk/government/uploads/system/uploads/attachment_data/file/415522/HO_Guidance_on_BBCS.pdf

9. Off Licence News. First conviction for selling alcohol “below cost” sees retailer fined [Internet]. 2016 [cited 2016 Jun 3]. Available from: http://www.offlicencenews.co.uk/news/fullstory.php/aid/15750/First_conviction_for_selling_alcohol__below_cost__sees_retailer_fined.html

For peer review only

For peer review only - http://bmjopen.bmj.com/site/about/guidelines.xhtml

10. Home Office. Duty plus VAT permitted price calculator (2015) [Internet]. 2016. Available from: https://www.gov.uk/government/publications/banning-the-sale-of-alcohol-below-the-cost-of-duty-plus-vat-march-2015

11. HM Government. The Government's Alcohol Strategy [Internet]. 2012 [cited 2016 Jun 3]. Available from: https://www.gov.uk/government/uploads/system/uploads/attachment_data/file/224075/alcohol-strategy.pdf

12. Scottish Government. Minimum Unit Pricing [Internet]. [cited 2017 Feb 17]. Available from: http://www.gov.scot/Topics/Health/Services/Alcohol/minimum-pricing

13. Court of Justice of the European Union. Press Release 155/15: The Scottish legislation introducing a minimum price per unit of alcohol is contrary to EU law if less restrictive tax measures can be introduced [Internet]. 2015 [cited 2017 Feb 17]. Available from: http://curia.europa.eu/jcms/upload/docs/application/pdf/2015-12/cp150155en.pdf

14. Judiciary of Scotland. Scotch Whisky Association and others v Lord Advocate and Advocate General for Scotland [Internet]. [cited 2016 Jul 11]. Available from: http://www.scotland-judiciary.org.uk/9/1672/Scotch-Whisky-Association-and-others-v-Lord-Advocate-and-Advocate-General-for-Scotland

15. BBC News. Bid to appeal against minimum alcohol pricing to Supreme Court. 2016 Nov 18 [cited 2017 Feb 17]; Available from: http://www.bbc.co.uk/news/uk-scotland-scotland-politics-38026073

16. Lifestyles Statistics Team,, Health and Social Care Information Centre. Statistics on Alcohol. England, 2015 [Internet]. 2015 [cited 2016 Jun 3]. Available from: http://www.hscic.gov.uk/catalogue/PUB17712/alc-eng-2015-rep.pdf

17. Cochrane Handbook. 21.4 Assessment of study quality and risk of bias [Internet]. [cited 2016 Jun 2]. Available from: http://handbook.cochrane.org/chapter_21/21_4_assessment_of_study_quality_and_risk_of_bias.htm

18. Hill AB. The Environment and Disease: Association or Causation? Proc R Soc Med. 1965 May;58(5):295–300.

19. Bhattacharya J, Gathmann C, Miller G. The Gorbachev Anti-Alcohol Campaign and Russia's Mortality Crisis. Am Econ J Appl Econ. 2013;5(2):232–60.

20. Herttua K, Mäkelä P, Martikainen P. Minimum Prices for Alcohol and Educational Disparities in Alcohol-related Mortality. Epidemiology. 2015 May;26(3):337–43.

21. Stockwell T, Zhao J, Martin G, Macdonald S, Vallance K, Treno A, et al. Minimum Alcohol Prices and Outlet Densities in British Columbia, Canada: Estimated Impacts on Alcohol-Attributable Hospital Admissions. Am J Public Health. 2013 Apr 18;103(11):2014–20.

22. Treisman D. Death and prices. Econ Transit. 2010 Apr 1;18(2):281–331.

For peer review only

For peer review only - http://bmjopen.bmj.com/site/about/guidelines.xhtml

23. Wald I, Moskalewicz J. Alcohol policy in a crisis situation. Br J Addict. 1984 Sep;79(3):331–5.

24. Zhao J, Stockwell T, Martin G, Macdonald S, Vallance K, Treno A, et al. The relationship between minimum alcohol prices, outlet densities and alcohol-attributable deaths in British Columbia, 2002–09. Addiction. 2013 Jun 1;108(6):1059–69.

25. Brennan A, Meng Y, Holmes J, Hill-McManus D, Meier PS. Potential benefits of minimum unit pricing for alcohol versus a ban on below cost selling in England 2014: modelling study. The BMJ. 2014 Sep 30;349:g5452.

26. Holmes J, Meng Y, Meier PS, Brennan A, Angus C, Campbell-Burton A, et al. Effects of minimum unit pricing for alcohol on different income and socioeconomic groups: a modelling study. The Lancet. 2014 May 16;383(9929):1655–64.

27. Meier PS, Purshouse R, Brennan A. Policy options for alcohol price regulation: the importance of modelling population heterogeneity. Addiction. 2010 Mar 1;105(3):383–93.

28. Meier PS, Holmes J, Angus C, Ally AK, Meng Y, Brennan A. Estimated Effects of Different Alcohol Taxation and Price Policies on Health Inequalities: A Mathematical Modelling Study. PLOS Med. 2016 Feb 23;13(2):e1001963.

29. Purshouse RC, Meier PS, Brennan A, Taylor KB, Rafia R. Estimated effect of alcohol pricing policies on health and health economic outcomes in England: an epidemiological model. The Lancet. 2010 Apr;375(9723):1355–64.

30. Sharma A, Etilé F, Sinha K. The Effect of Introducing a Minimum Price on the Distribution of Alcohol Purchase: A Counterfactual Analysis. Health Econ. 2016 Sep;25(9):1182–200.

31. Vandenberg B, Sharma A. Are Alcohol Taxation and Pricing Policies Regressive? Product-Level Effects of a Specific Tax and a Minimum Unit Price for Alcohol. Alcohol Alcohol Oxf Oxfs. 2016 Jul;51(4):493–502.

32. Black H, Gill J, Chick J. The price of a drink: levels of consumption and price paid per unit of alcohol by Edinburgh's ill drinkers with a comparison to wider alcohol sales in Scotland. Addiction. 2011 Apr 1;106(4):729–36.

33. Callinan S, Room R, Livingston M, Jiang H. Who Purchases Low-Cost Alcohol in Australia? Alcohol Alcohol. 2015 Jun 24;agv066.

34. Cousins G, Mongan D, Barry J, Smyth B, Rackard M, Long J. Potential Impact of Minimum Unit Pricing for Alcohol in Ireland: Evidence from the National Alcohol Diary Survey. Alcohol Alcohol. 2016 Nov 26;51(6):734–40.

35. Crawford MJ, Parry AMH, Weston ARW, Seretis D, Zauter-Tutt M, Hussain A, et al. Relationship between price paid for off-trade alcohol, alcohol consumption and income in England: a cross-sectional survey. Alcohol Alcohol Oxf Oxfs. 2012 Dec;47(6):738–42.

36. Falkner C, Christie G. The effect of alcohol price on dependent drinkers' alcohol consumption. N Z Med J. 2015;128(1427).

For peer review only

For peer review only - http://bmjopen.bmj.com/site/about/guidelines.xhtml

37. Forsyth AJM, Ellaway A, Davidson N. How Might the Alcohol Minimum Unit Pricing (MUP) Impact upon Local Off-Sales Shops and the Communities Which They Serve? Alcohol Alcohol. 2014 Jan 1;49(1):96–102.

38. Ludbrook PA, Petrie D, McKenzie L, Farrar S. Tackling alcohol misuse: Purchasing patterns affected by minimum pricing for alcohol. Appl Health Econ Health Policy. 2012 Aug 6;10(1):51–63.

39. Sharma A, Vandenberg B, Hollingsworth B. Minimum Pricing of Alcohol versus Volumetric Taxation: Which Policy Will Reduce Heavy Consumption without Adversely Affecting Light and Moderate Consumers? PLoS ONE. 2014 Jan 22;9(1):e80936.

40. Sheron N, Chilcott F, Matthews L, Challoner B, Thomas M. Impact of minimum price per unit of alcohol on patients with liver disease in the UK. Clin Med. 2014 Aug 1;14(4):396–403.

41. Babor TF, Mendelson JH, Greenberg I, Kuehnle J. Experimental analysis of the 'happy hour": effects of purchase price on alcohol consumption. Psychopharmacology (Berl). 1978 Jun 15;58(1):35–41.

42. Seaman P, Edgar F, Ikegwuonu T. The role of alcohol price in young adult drinking cultures in Scotland. Drugs Educ Prev Policy. 2013 Aug 1;20(4):278–85.

43. Wagenaar AC, Salois MJ, Komro KA. Effects of beverage alcohol price and tax levels on drinking: a meta-analysis of 1003 estimates from 112 studies. Addict Abingdon Engl. 2009 Feb;104(2):179–90.

44. Wagenaar AC, Tobler AL, Komro KA. Effects of alcohol tax and price policies on morbidity and mortality: a systematic review. Am J Public Health. 2010 Nov;100(11):2270–8.

45. Angus C, Holmes J, Pryce R, Meier P, Brennan A. Model-based appraisal of the comparative impact of Minimum Unit Pricing and taxation policies in Scotland An adaptation of the Sheffield Alcohol Policy Model version 3 [Internet]. ScHARR, University of Sheffield; 2016 Apr [cited 2016 Apr 7]. Available from: https://www.shef.ac.uk/polopoly_fs/1.565373!/file/Scotland_report_2016.pdf

46. Booth A, Brennan A, Meier PS, O'Reilly D, Purshouse R, Stockwell T, et al. Independent review of the effects of alcohol pricing and promotion: part A – systematic reviews. Project Report for the Department of Health September 2008. ScHARR University of Sheffield; 2008.

47. Brennan A, Purshouse R, Taylor K, Rafia R. Independent review of the effects of alcohol pricing and promotion: part B. Modelling the Potential Impact of Pricing and Promotion Policies for Alcohol in England: Results from the Sheffield Alcohol Policy Model Version 2008(1-1) [Internet]. ScHARR University of Sheffield; 2008. Available from: https://www.shef.ac.uk/polopoly_fs/1.95621!/file/PartB.pdf

48. Hill McManus D, Brennan A, Stockwell T, Giesbrecht N, Thomas G, Zhao J, et al. Model-based appraisal of alcohol minimum pricing in Ontario and British Columbia: A Canadian adaptation of the Sheffield Alcohol Policy Model Version 2 [Internet]. 2012.

For peer review only

For peer review only - http://bmjopen.bmj.com/site/about/guidelines.xhtml

Available from: http://www.uvic.ca/research/centres/carbc/assets/docs/report-model-based-appraisal.pdf

49. Institute for Fiscal Studies. The Impact of Introducing a Minimum Price on Alcohol in Britain. IFS Briefing Note BN109. 2010.

50. Institute for Fiscal Studies. Price-based measures to reduce alcohol consumption. IFS Briefing Note BN138. 2013.

51. Meng Y, Purshouse R, Brennan A, Meier PS. Model-based appraisal of alcohol minimum pricing and off-licensed trade discount bans in Scotland using the Sheffield Alcohol Policy Model (v.2): Second update based on newly available data [Internet]. 2010. Available from: http://www.shef.ac.uk/polopoly_fs/1.96510!/file/scotlandupdate.pdf

52. Rabinovich L, Brutscher P-B, de Vries H, Tiessen J, Clift J, Reding A. The affordability of alcoholic beverages in the European Union. 2012;

53. Gallet CA. The Demand for Alcohol: A Meta-Analysis of Elasticities [Internet]. Rochester, NY: Social Science Research Network; 2007 May [cited 2016 Jan 6]. Report No.: ID 985689. Available from: http://papers.ssrn.com/abstract=985689

54. Gruenewald PJ, Ponicki WR, Holder HD, Romelsjö A. Alcohol prices, beverage quality, and the demand for alcohol: quality substitutions and price elasticities. Alcohol Clin Exp Res. 2006 Jan;30(1):96–105.

55. Ally AK, Meng Y, Chakraborty R, Dobson PW, Seaton JS, Holmes J, et al. Alcohol tax pass-through across the product and price range: do retailers treat cheap alcohol differently? Addiction. 2014 Dec 1;109(12):1994–2002.

56. Rabinovich L, Hunt P, Staetsky L, Goshev S, Nolte E, Pedersen JS, et al. Further study on the affordability of alcoholic beverages in the EU [Internet]. 2012 [cited 2017 Feb 17]. Available from: http://www.rand.org/pubs/technical_reports/TR1203.html

57. Burton R, Henn C, Lavoie D, O'Connor R, Perkins C, Sweeney K, et al. A rapid evidence review of the effectiveness and cost-effectiveness of alcohol control policies: an English perspective. The Lancet [Internet]. 2016 Dec 2 [cited 2017 Feb 17];0(0). Available from: http://www.thelancet.com/journals/lancet/article/PIIS0140-6736(16)32420-5/abstract

For peer review only

For peer review only - http://bmjopen.bmj.com/site/about/guidelines.xhtml

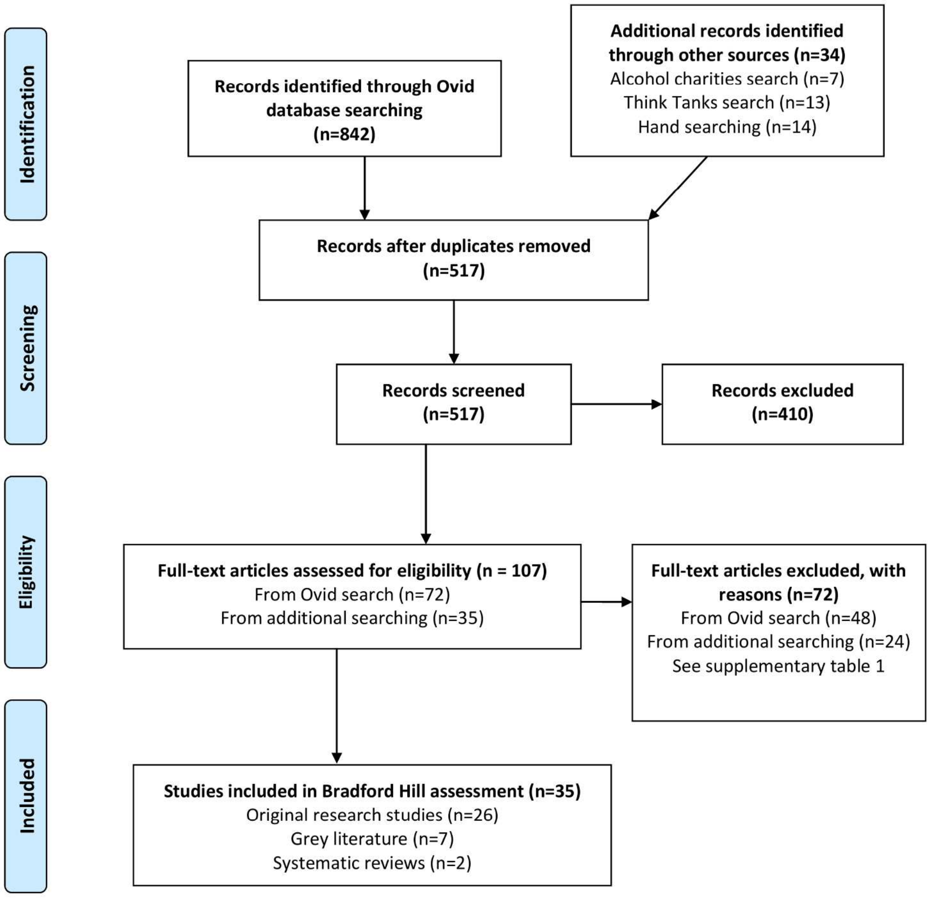

PRISMA flow diagram of studies in this systematic review

619x584mm (96 x 96 DPI)

Figure 2: Areas of evidence for the effectiveness of minimum pricing of alcohol, with key examples

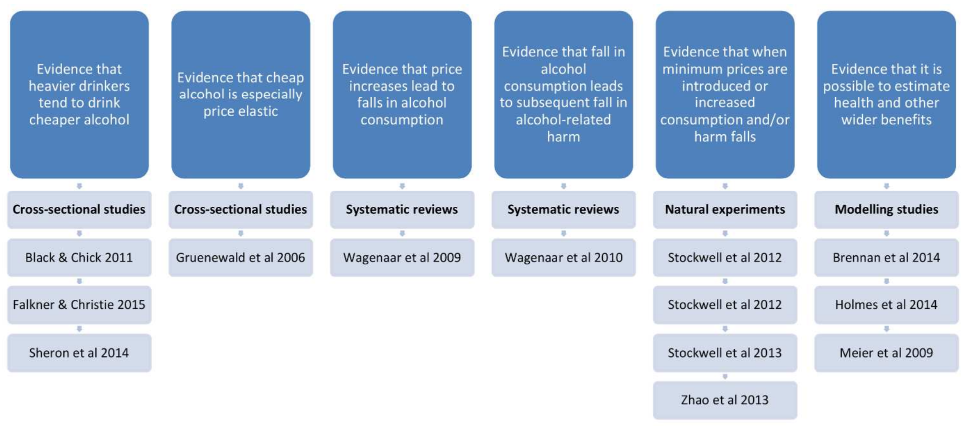

This model shows that different study types tended to produce evidence of effectiveness of minimum pricing in relation to different outcomes. Studies cited in the figure are key examples of the literature in that area and do not represent an exhaustive list.

784x438mm (96 x 96 DPI)

For peer review only - http://bmjopen.bmj.com/site/about/guidelines.xhtml

Online supplementary table: identified studies where full text assessed, then not included in review

Source	Author and year published	Study type	Reason excluded
Ovid search	Aage 2012 (1)	Time series analysis	Affordability, not price
	Ayyagari 2013 (2)	Econometric analysis of price and consumption data	Price elasticities of demand only
	Bellis 2009 (3)	Cross-sectional surveys	Association between drink type and price, and drink type and harms, but not directly reported in paper
	Bennetts 2008 (4)	Review (not systematic)	Review of a report (not a research study)
	Burton 2016 (5)	Rapid evidence review (not systematic)	Review of effectiveness and cost-effectiveness of range of policy options (including MUP), not systematic
	Callinan 2015 (6)	Editorial (not a research study)	Editorial about different pricing, taxation and afforability measures, not a research study
	Casswell 2014 (7)	Cross-sectional survey	Studies price paid for alcohol, but not in relation to a proposed minimum price (price simply dichotomised into either high or low)
	Chalmers 2013 (8)	Commentary (not a research study)	Is a commentary about challenges to MUP e.g. industry and the law
	Chaloupka 2002 (9)	Review (not systematic)	Review of previous economic studies (not systematic)
	Chick 2012 (10)	Editorial (not a research study)	Is about bans on multi-buys, not minimum price
	Cook 2014 (11)	Analysis of cross-sectional survey data and alcohol policies	Affordability not price (GDP PPP)
	Duffy 1981 (12)	Econometric time series analysis	Talks about elasticities in relation to duty rises not MUP. NB is M duffy, not J duffy
	Giesbrecht 2003 (13)	Review (not systematic)	Review of previous studies (not systematic)
	Giesbrecht 2015 (14)	Policy analysis	Does not relate price intervention to consumption or harm
	Giesbrecht 2016 (15)	Comparison of alcohol policies in different Canadian provinces	Does not relate different prices to different outcomes in terms of consumption or harm

For peer review only - http://bmjopen.bmj.com/site/about/guidelines.xhtml

For peer review only

	Gilligan 2012 (16)	Cross-sectional survey and policy analysis	Price changes, not minimum prices
	Grossman 2004 (17)	Economic analysis	Tax (federal excise tax increase) not MUP
	Hadland 2015 (18)	Policy analysis	Effects of numerous alcohol policies considered together
	Heeb 2003 (19)	Natural experiment: longitudinal survey 3 months before and 3 months after price change	Tax, not price intervention
	Hogan 2006 (20)	Natural experiment/evaluation	Tax, not price intervention
	Jónsson 2013 (21)	Cross-sectional surveys and routine data	Policies included tax and other supply side policies, but not MUP
	Katikireddi 2012 (22)	Commentary (not a research study)	Is a commentary about legal considerations of MUP, not effectiveness
	Katikireddi 2014 (23)	Qualitative study	Stakeholder views of MUP, not effectiveness
	Khaltourina 2015 (24)	Cross-sectional surveys and routine data	Tax, not price intervention
	Knibbe 2014 (25)	Analysis of cross-sectional survey data and alcohol policies	No price intervention studied, price investigated more as a covariate
	Lindeman 2013 (26)	Cross-sectional surveys and routine data	Tax, not price intervention
	Lonsdale 2012 (27)	Qualitative study	Public opinion of policy, nothing about effectiveness
	Mäkelä 2009 (28)	Review (not systematic) and analysis of routine data	Tax, not price intervention
	McCambridge 2014 (29)	Document analysis and qualitative interviews	Corporate lobbying, not the effectiveness of MUP
	Meng 2014 (30)	Econometric analysis using pseudo panel	Price elasticities of demand only
	Nelson 2013 (31)	Review (not systematic)	Conflict of interest (funded by International Center for Alcohol Policies).
	Nelson 2014 (affordability) (32)	Analysis of routine data and economic modelling	Affordability not price, and conflict of interest (funded by International Center for Alcohol Policies).
	Nelson 2014 (elasticities) (33)	Economic modelling	Conflict of interest (funded by International Center for Alcohol Policies).

For peer review only - http://bmjopen.bmj.com/site/about/guidelines.xhtml

	Nelson 2014 (gender) (34)	Systematic review	Conflict of interest (funded by International Center for Alcohol Policies).
	Nelson & McNall 2016 (35)	Review (not systematic)	Conflict of interest (funded by International Alliance for Responsible Drinking)
	Nicholls & Greenaway 2015 (36)	Policy analysis	Is about policy framing, not the effectiveness of MUP
	Record 2009 (37)	Modelling study	Conflict of interest – both authors were members of the Alcohol Health Alliance which is campaigning for MUP
	Rush 1986 (38)	Analysis of routine data	Affordability, not price
	Shi 2011 (39)	Econometric analysis of price and consumption data	Price elasticities of demand only
	Skov 2011 (40)	Natural experiment	Tax, not price intervention
	Sloan 1994 (41)	Analysis of routine data 1982-1988	Price changes, not minimum prices
	Stockwell 2012 (42)	Letter to journal (not a research study)	Letter concerning homelessness and price paid for alcohol
	Stockwell 2013 (43)	Letter to journal (not a research study)	Letter to editor about UK industry criticism of Canadian research on MUP, not a research study
	Sutton & Godfrey 1995 (44)	Cross-sectional survey	Price changes, not minimum prices
	Tian 2011 (45)	Econometric analysis of price and consumption data	Price elasticities of demand only
	Wall & Casswell 2013 (46)	Time series analysis and econometric modelling	Price changes, not minimum prices
	Xuan 2015 (47)	Analysis of cross-sectional survey data and alcohol policies	Price and tax considered together
	Yashkin 2013 (48)	Econometric analysis of price and consumption data	Price elasticities of demand only
Hand searching &	Ally 2014 (49)	Analysis of VAT and price data	Tax, not price intervention
	Anderson 2009 (50)	Lancet 'Series' article – a	Not an original research study.

For peer review only - http://bmjopen.bmj.com/site/about/guidelines.xhtml

grey literature		commentary/review	
	Byrnes 2013 (51)	Repeated cross-sectional survey	Price changes, not minimum prices
	Centre for Economics and Business Research 2010 (52)	Report (not a research study)	Conflict of interest (funded by SAB Miller). Is a critique of Sheffield modelling, not a research study.
	Craven 2013 (53)	Economic report	Published in the journal of the Institute of Economic Affairs, which has a conflict of interest.
	Duffy & Snowdon 2012 (54)	Report chapters (not a research study)	Is a critique of Sheffield modelling, not a research study.
	Fitzgerald & Angus 2015 (55)	Report (not a research study)	Is a report about use of evidence in policymaking, not about effectiveness of MUP. Also not a research study.
	Gallet 2007 (56)	Meta-analysis	Is looking at elasticities of demand for alcohol, not the effectiveness of MUP.
	Gray 2000 (57)	Natural experiment	Restrictions do not include any price interventions
	Gruenewald 2006 (58)	Time series analysis/modelling	Price changes, not minimum prices, but included in narrative
	Hilton 2014 (59)	Content analysis of UK newsprint	Media representations of MUP, not effectiveness
	Home Office 2011 (60)	'Summary review'/report (not a research study)	Replicates what we have elsewhere, but in less detail. Also not a research study.
	Institute for Fiscal Studies 2011 (61)	Report (not a research study)	Is not about effectiveness of MUP
	Institute for Fiscal Studies 2013 (62)	Report (not a research study)	Is not looking at effectiveness of MUP, is comparing Govt and industry revenue under MUP and higher tax
	Institute of Alcohol Studies (Goodliffe) 2014 (63)	Webpage	Is discussing legal issues, not effectiveness of MUP
	Kisely & Lawence 2015 (64)	Natural experiment	Tax, not price intervention
	Ludbrook 2010 (65)	Secondary analysis of Expenditure and Food Survey	Describes purchasing patterns of low price alcohol in Scotland. Not about MUP.
	Ornstein 1983 (66)	Literature review book chapter (not systematic)	Price elasticities of demand only

For peer review only - http://bmjopen.bmj.com/site/about/guidelines.xhtml

	Rabinovch 2012 (67)	Technical report (contains data)	Does not assess effectiveness of MUP
	Radaev 2015 (68)	Time series analysis	Paper explored the effect of price interventions on consumption of homemade alcohol, not the effectiveness of MUP
	Robinson 2013 (69)	Letter to journal (not a research study)	Letter to editor about evidence for MUP, not a research study
	Snowdon 2015 (70)	Book chapter	Is a critique of Sheffield modelling, not a research study. Institute of Economic Affairs also has a conflict of interest.
	Wine and Spirits Trade Association n.d. (71)	Press release	Press release critiquing Zhao 2013 paper, not a research study
	World Health Organisation 2014 (72)	Report	Does not discuss effectiveness of MUP

For peer review only

For peer review only - http://bmjopen.bmj.com/site/about/guidelines.xhtml

References

1. Aage H. Alcohol in Greenland 1951–2010: consumption, mortality, prices. Int J Circumpolar Health [Internet]. 2012 Dec 17 [cited 2016 Feb 27];71. Available from: http://www.ncbi.nlm.nih.gov/pmc/articles/PMC3525923/

2. Ayyagari P, Deb P, Fletcher J, Gallo W, Sindelar JL. Understanding heterogeneity in price elasticities in the demand for alcohol for older individuals. Health Econ. 2013 Jan;22(1):89–105.

3. Bellis MA, Phillips-Howard PA, Hughes K, Hughes S, Cook PA, Morleo M, et al. Teenage drinking, alcohol availability and pricing: a cross-sectional study of risk and protective factors for alcohol-related harms in school children. BMC Public Health. 2009 Oct 9;9(1):1–12.

4. Bennetts R. Russell Bennetts. Alcohol: Price, Policy and Public Health. Alcohol Alcohol. 2008 Mar 1;43(2):123–123.

5. Burton R, Henn C, Lavoie D, O'Connor R, Perkins C, Sweeney K, et al. A rapid evidence review of the effectiveness and cost-effectiveness of alcohol control policies: an English perspective. The Lancet [Internet]. 2016 Dec 2 [cited 2017 Feb 17];0(0). Available from: http://www.thelancet.com/journals/lancet/article/PIIS0140-6736(16)32420-5/abstract

6. Callinan S, Room R, Dietze P. Alcohol Price Policies as an Instrument of Health Equity: Differential Effects of Tax and Minimum Price Measures. Alcohol Alcohol Oxf Oxfs. 2015 Nov;50(6):629–30.

7. Casswell S, Huckle T, Wall M, Yeh LC. International alcohol control study: pricing data and hours of purchase predict heavier drinking. Alcohol Clin Exp Res. 2014 May;38(5):1425–31.

8. Chalmers J, Carragher N, Davoren S, O'Brien P. Real or perceived impediments to minimum pricing of alcohol in Australia: Public opinion, the industry and the law. Int J Drug Policy. 2013 Nov;24(6):517–23.

9. Chaloupka FJ, Grossman M, Saffer H. The effects of price on alcohol consumption and alcohol-related problems. Alcohol Res Health J Natl Inst Alcohol Abuse Alcohol. 2002;26(1):22–34.

10. Chick J. 16 for the Price of 10: Effects of a Ban on Multi-Buy Alcohol. Alcohol Alcohol. 2012 Feb 22;47(2):83–83.

11. Cook WK, Bond J, Greenfield TK. Are alcohol policies associated with alcohol consumption in low- and middle-income countries? Addict Abingdon Engl. 2014 Jul;109(7):1081–90.

12. Duffy M. The influence of prices, consumer incomes and advertising upon the demand for alcoholic drink in the United Kindgom: an econometric study. Alcohol Alcohol. 1981 Sep 21;16(4):200–9.

13. Giesbrecht N, Greenfield TK. Preventing Alcohol-Related Problems in the US Through Policy: Media Campaigns, Regulatory Approaches and Environmental Interventions. J Prim Prev. 2003 Sep;24(1):63–104.

For peer review only - http://bmjopen.bmj.com/site/about/guidelines.xhtml

14. Giesbrecht N, Wettlaufer A, Thomas G, Stockwell T, Thompson K, April N, et al. Pricing of alcohol in Canada: A comparison of provincial policies and harm-reduction opportunities. Drug Alcohol Rev. 2015 Nov 4;

15. Giesbrecht N, Wettlaufer A, Thomas G, Stockwell T, Thompson K, April N, et al. Pricing of alcohol in Canada: A comparison of provincial policies and harm-reduction opportunities. Drug Alcohol Rev. 2016 May;35(3):289–97.

16. Gilligan C, Kuntsche E, Gmel G. Adolescent drinking patterns across countries: associations with alcohol policies. Alcohol Alcohol Oxf Oxfs. 2012 Dec;47(6):732–7.

17. Grossman M. Individual Behaviors and Substance Use: The Role of Price [Internet]. National Bureau of Economic Research; 2004 Dec [cited 2016 Feb 27]. Report No.: 10948. Available from: http://www.nber.org/papers/w10948

18. Hadland SE, Xuan Z, Blanchette JG, Heeren TC, Swahn MH, Naimi TS. Alcohol Policies and Alcoholic Cirrhosis Mortality in the United States. Prev Chronic Dis [Internet]. 2015 Oct 15 [cited 2016 Feb 27];12. Available from: http://www.cdc.gov/pcd/issues/2015/15_0200.htm

19. Heeb J-L, Gmel G, Zurbrügg C, Kuo M, Rehm J. Changes in alcohol consumption following a reduction in the price of spirits: a natural experiment in Switzerland. Addict Abingdon Engl. 2003 Oct;98(10):1433–46.

20. Hogan E, BOFFA J, ROSEWARNE C, BELL S, CHEE DA. What price do we pay to prevent alcohol-related harms in Aboriginal communities? The Alice Springs trial of liquor licensing restrictions. Drug Alcohol Rev. 2006;25(3):207–212.

21. Jónsson RM, Kristjánsson S. Alcohol policy and public opinion in Iceland, 1989–2012. Nord Stud Alcohol Drugs. 2013;30(6):539–549.

22. Katikireddi SV, McLean JA. Introducing a minimum unit price for alcohol in Scotland: considerations under European Law and the implications for European public health. Eur J Public Health. 2012 Aug 1;22(4):457–8.

23. Katikireddi SV, Bond L, Hilton S. Perspectives on econometric modelling to inform policy: a UK qualitative case study of minimum unit pricing of alcohol. Eur J Public Health. 2014 Jun 1;24(3):490–5.

24. Khaltourina D, Korotayev A. Effects of Specific Alcohol Control Policy Measures on Alcohol-Related Mortality in Russia from 1998 to 2013. Alcohol Alcohol. 2015 Sep 1;50(5):588–601.

25. Knibbe RA, Derickx M, Allamani A, Massini G. Alcohol Consumption and its Related Harms in the Netherlands Since 1960: Relationships With Planned and Unplanned Factors. Subst Use Misuse. 2014 Oct 15;49(12):1589–600.

26. Lindeman M, Karlsson T, Österberg E. Public opinions, alcohol consumption and policy changes in Finland, 1993–2013. Nord Stud Alcohol Drugs. 2013;30(6):507–524.

For peer review only - http://bmjopen.bmj.com/site/about/guidelines.xhtml

27. Lonsdale AJ, Hardcastle SJ, Hagger MS. A minimum price per unit of alcohol: A focus group study to investigate public opinion concerning UK government proposals to introduce new price controls to curb alcohol consumption. BMC Public Health. 2012 Nov 23;12(1):1023.

28. Mäkelä P, Osterberg E. Weakening of one more alcohol control pillar: a review of the effects of the alcohol tax cuts in Finland in 2004. Addict Abingdon Engl. 2009 Apr;104(4):554–63.

29. McCambridge J, Hawkins B, Holden C. Vested Interests in Addiction Research and Policy. The challenge corporate lobbying poses to reducing society's alcohol problems: insights from UK evidence on minimum unit pricing. Addiction. 2014 Feb 1;109(2):199–205.

30. Meng Y, Brennan A, Purshouse R, Hill-McManus D, Angus C, Holmes J, et al. Estimation of own and cross price elasticities of alcohol demand in the UK--A pseudo-panel approach using the Living Costs and Food Survey 2001-2009. J Health Econ. 2014 Mar;34:96–103.

31. Nelson JP. Does Heavy Drinking by Adults Respond to Higher Alcohol Prices and Taxes? A Survey and Assessment. Econ Anal Policy. 2013 Dec;43(3):265–91.

32. Nelson JP. Alcohol Affordability and Alcohol Demand: Cross-Country Trends and Panel Data Estimates, 1975 to 2008. Alcohol Clin Exp Res. 2014 Apr 1;38(4):1167–75.

33. Nelson JP. Estimating the Price Elasticity of Beer: Meta-Analysis of Data with Heterogeneity, Dependence, and Publication Bias [Internet]. Rochester, NY: Social Science Research Network; 2013 Jan [cited 2016 Feb 27]. Report No.: ID 2200492. Available from: http://papers.ssrn.com/abstract=2200492

34. Nelson JP. Gender differences in alcohol demand: a systematic review of the role of prices and taxes. Health Econ. 2014 Oct;23(10):1260–80.

35. Nelson JP, McNall AD. What happens to drinking when alcohol policy changes? A review of five natural experiments for alcohol taxes, prices, and availability. Eur J Health Econ. 2016 Apr 7;1–18.

36. Nicholls J, Greenaway J. What is the problem?: Evidence, politics and alcohol policy in England and Wales, 2010–2014. Drugs Educ Prev Policy. 2015 Mar 4;22(2):135–42.

37. Record C, Day C. Britain's alcohol market: how minimum alcohol prices could stop moderate drinkers subsidising those drinking at hazardous and harmful levels. Clin Med. 2009 Oct 1;9(5):421–5.

38. Rush B, Steinberg M, Brook R. The relationships among alcohol availability, alcohol consumption and alcohol-related damage in the Province of Ontario and the State of Michigan 1955-1982. Adv Alcohol Subst Abuse. 1986;5(4):33–45.

39. Shi Y. Three Essays on Economics of Health Behavior in China [Internet]. 2011 [cited 2016 Mar 20]. Available from: http://www.rand.org/pubs/rgs_dissertations/RGSD287.html

40. Skov SJ, Chikritzhs TN, Kypri K, Miller PG, Hall WD, Daube MM, et al. Is the "alcopops" tax working? Probably yes but there is a bigger picture. Med J Aust [Internet]. 2011 [cited 2015 Nov

For peer review only - http://bmjopen.bmj.com/site/about/guidelines.xhtml

9];195(2). Available from: https://www.mja.com.au/journal/2011/195/2/alcopops-tax-working-probably-yes-there-bigger-picture

41. Sloan FA, Reilly BA, Schenzler C. Effects of prices, civil and criminal sanctions, and law enforcement on alcohol-related mortality. J Stud Alcohol. 1994 Jul;55(4):454–65.

42. Stockwell T, Williams N, Pauly B. Working and waiting: Homeless drinkers responses to less affordable alcohol. Drug Alcohol Rev. 2012 Sep 1;31(6):823–4.

43. Stockwell T, Zhao J, Martin G, Macdonald S, Vallance K, Treno A, et al. Misleading UK alcohol industry criticism of Canadian research on minimum pricing. Addiction. 2013 Jun 1;108(6):1172–3.

44. Sutton M, Godfrey C. A grouped data regression approach to estimating economic and social influences on individual drinking behaviour. Health Econ. 1995 May 1;4(3):237–47.

45. Tian G, Liu F. Is the demand for alcoholic beverages in developing countries sensitive to price? Evidence from China. Int J Environ Res Public Health. 2011 Jun;8(6):2124–31.

46. Wall M, Casswell S. Affordability of alcohol as a key driver of alcohol demand in New Zealand: a co-integration analysis. Addict Abingdon Engl. 2013 Jan;108(1):72–9.

47. Xuan Z, Blanchette J, Nelson TF, Heeren T, Oussayef N, Naimi TS. The alcohol policy environment and policy subgroups as predictors of binge drinking measures among US adults. Am J Public Health. 2015 Apr;105(4):816–22.

48. Yashkin A. The Dynamics of Alcohol Consumption in the Russian Federation: Implications of Using Price Related Policies to Control Alcohol Use. Grad Theses Diss [Internet]. 2013 Jan 1; Available from: http://scholarcommons.usf.edu/etd/4968

49. Ally AK, Meng Y, Chakraborty R, Dobson PW, Seaton JS, Holmes J, et al. Alcohol tax pass-through across the product and price range: do retailers treat cheap alcohol differently? Addiction. 2014 Dec 1;109(12):1994–2002.

50. Anderson P, Chisholm D, Fuhr DC. Effectiveness and cost-effectiveness of policies and programmes to reduce the harm caused by alcohol. Lancet Lond Engl. 2009 Jun 27;373(9682):2234–46.

51. Byrnes J, Shakeshaft A, Petrie D, Doran C. Can harms associated with high-intensity drinking be reduced by increasing the price of alcohol? Drug Alcohol Rev. 2013 Jan;32(1):27–30.

52. Centre for Economics and Business Research. Minimum Alcohol Pricing: A targeted measure? Report to the Scottish Parliamentary Health and Sport Committee [Internet]. London; 2010 Aug. Available from: http://www.ias.org.uk/uploads/pdf/Price%20docs/Updated-Sheffield-Scotland-v2-August-20103.pdf

53. Craven BM, Marlow ML, Shiers AF. The Economics of Minimum Pricing for Alcohol. Econ Aff. 2013 Jun 1;33(2):174–89.

For peer review only - http://bmjopen.bmj.com/site/about/guidelines.xhtml

54. Duffy J, Snowdon C. The minimal evidence for minimum pricing [Internet]. Adam Smith Institute; Available from: http://www.adamsmith.org/sites/default/files/research/files/ASI_SAPM.pdf

55. Fitzgerald N, Angus C. Four Nations: How Evidence-based are Alcohol Policies and Programmes across the UK?. London: [Internet]. London: Alliance for Useful Evidence/Alcohol Health Alliance.; 2015. Available from: http://www.alliance4usefulevidence.org/assets/Four-Nations-v3.pdf

56. Gallet CA. The Demand for Alcohol: A Meta-Analysis of Elasticities [Internet]. Rochester, NY: Social Science Research Network; 2007 May [cited 2016 Jan 6]. Report No.: ID 985689. Available from: http://papers.ssrn.com/abstract=985689

57. Gray D, Saggers S, Atkinson D, Sputore B, Bourbon D. Beating the grog: an evaluation of the Tennant Creek liquor licensing restrictions. Aust N Z J Public Health. 2000 Feb;24(1):39–44.

58. Gruenewald PJ, Ponicki WR, Holder HD, Romelsjö A. Alcohol prices, beverage quality, and the demand for alcohol: quality substitutions and price elasticities. Alcohol Clin Exp Res. 2006 Jan;30(1):96–105.

59. Hilton S, Wood K, Patterson C, Katikireddi SV. Implications for alcohol minimum unit pricing advocacy: What can we learn for public health from UK newsprint coverage of key claim-makers in the policy debate? Soc Sci Med. 2014 Feb;102:157–64.

60. Home Office. The likely impacts of increasing alcohol price: a summary review of the evidence base [Internet]. 2011 Jan. Available from: https://www.gov.uk/government/uploads/system/uploads/attachment_data/file/98100/impacts-alcohol-price-review.pdf

61. Institute for Fiscal Studies. Alcohol pricing and taxation policies. IFS Briefing Note BN124. 2011.

62. Institute for Fiscal Studies. Price-based measures to reduce alcohol consumption. IFS Briefing Note BN138. 2013.

63. Goodliffe J. Applying a minimum price to alcohol (Institute of Alcohol Studies) [Internet]. 2014. Available from: http://www.ias.org.uk/What-we-do/Publication-archive/Alcohol-Alert/October-2014/Applying-a-minimum-price-to-alcohol.aspx

64. Kisely S, Lawrence D. A time series analysis of alcohol-related presentations to emergency departments in Queensland following the increase in alcopops tax. J Epidemiol Community Health. 2015 Sep 16;jech-2015-205666.

65. Ludbrook A. Purchasing Patterns for Low Price Off Sales Alcohol: Evidence from the Expenditure and Food Survey [Internet]. Available from: http://www.shaap.org.uk/images/UserFiles/File/Reports%20and%20Briefings/Briefing%20-%20Purchase%20of%20low-price%20alcohol%20analysis.pdf

66. Ornstein SI, Levy D. Price and Income Elasticities of Demand for Alcoholic Beverages. In: Galanter M, Begleiter H, Cicero T, Deitrich R, Goodwin DW, Gottheil E, et al., editors. Genetics

For peer review only - http://bmjopen.bmj.com/site/about/guidelines.xhtml

Behavioral Treatment Social Mediators and Prevention Current Concepts in Diagnosis [Internet]. Boston, MA: Springer US; 1983. p. 303–45. Available from: http://dx.doi.org/10.1007/978-1-4613-3617-4_18

67. Rabinovich L, Brutscher P-B, de Vries H, Tiessen J, Clift J, Reding A. The affordability of alcoholic beverages in the European Union. 2012;

68. Radaev V. Impact of a New Alcohol Policy on Homemade Alcohol Consumption and Sales in Russia. Alcohol Alcohol. 2015 May 1;50(3):365–72.

69. Robinson M, McCartney G, Beeston C. What is convincing evidence on alcohol pricing? BMJ. 2013 Aug 20;347:f5102.

70. Snowdon C. Chapter 10. Minimum unit pricing. In: Flaws and Ceilings: Price controls and the damage they cause [Internet]. [cited 2015 Nov 9]. p. 177–97. Available from: http://www.iea.org.uk/publications/research/flaws-and-ceilings-price-controls-and-the-damage-they-cause

71. Wine and Spirits Trade Association. New claims that increasing the cost of alcohol saves lives are misleading [Internet]. Available from: http://www.wsta.co.uk/press/634-new-claims-that-increasing-the-cost-of-alcohol-saves-lives-are-misleading

72. World Health Organization. European status report on alcohol and health 2014. Pricing policies [Internet]. World Health Organization; 2014. Available from: http://www.euro.who.int/__data/assets/pdf_file/0003/244902/Pricing-policies.pdf

For peer review only

For peer review only - http://bmjopen.bmj.com/site/about/guidelines.xhtml

PRISMA 2009 Checklist

Section/topic	#	Checklist item	Reported on page #
**TITLE**			
Title	1	Identify the report as a systematic review, meta-analysis, or both.	1
**ABSTRACT**			
Structured summary	2	Provide a structured summary including, as applicable: background; objectives; data sources; study eligibility criteria, participants, and interventions; study appraisal and synthesis methods; results; limitations; conclusions and implications of key findings; systematic review registration number.	2
**INTRODUCTION**			
Rationale	3	Describe the rationale for the review in the context of what is already known.	3-4
Objectives	4	Provide an explicit statement of questions being addressed with reference to participants, interventions, comparisons, outcomes, and study design (PICOS).	4
**METHODS**			
Protocol and registration	5	Indicate if a review protocol exists, if and where it can be accessed (e.g., Web address), and, if available, provide registration information including registration number.	no published protocol
Eligibility criteria	6	Specify study characteristics (e.g., PICOS, length of follow-up) and report characteristics (e.g., years considered, language, publication status) used as criteria for eligibility, giving rationale.	4
Information sources	7	Describe all information sources (e.g., databases with dates of coverage, contact with study authors to identify additional studies) in the search and date last searched.	4
Search	8	Present full electronic search strategy for at least one database, including any limits used, such that it could be repeated.	4
Study selection	9	State the process for selecting studies (i.e., screening, eligibility, included in systematic review, and, if applicable, included in the meta-analysis).	4-6
Data collection process	10	Describe method of data extraction from reports (e.g., piloted forms, independently, in duplicate) and any processes for obtaining and confirming data from investigators.	4-6
Data items	11	List and define all variables for which data were sought (e.g., PICOS, funding sources) and any assumptions and simplifications made.	Tables 2+3
Risk of bias in individual studies	12	Describe methods used for assessing risk of bias of individual studies (including specification of whether this was done at the study or outcome level), and how this information is to be used in any data synthesis.	4
Summary measures	13	State the principal summary measures (e.g., risk ratio, difference in means).	Not stated, as all summary measures included
Synthesis of results	14	Describe the methods of handling data and combining results of studies, if done, including measures of consistency (e.g., I^2) for each meta-analysis.	4, and Table 1

For peer review only

PRISMA 2009 Checklist

Section/topic	#	Checklist item	Reported on page #
Risk of bias across studies	15	Specify any assessment of risk of bias that may affect the cumulative evidence (e.g., publication bias, selective reporting within studies).	4
Additional analyses	16	Describe methods of additional analyses (e.g., sensitivity or subgroup analyses, meta-regression), if done, indicating which were pre-specified.	4
**RESULTS**			
Study selection	17	Give numbers of studies screened, assessed for eligibility, and included in the review, with reasons for exclusions at each stage, ideally with a flow diagram.	Figure 1 and online supplementary file
Study characteristics	18	For each study, present characteristics for which data were extracted (e.g., study size, PICOS, follow-up period) and provide the citations.	Tables 2+3
Risk of bias within studies	19	Present data on risk of bias of each study and, if available, any outcome level assessment (see item 12).	Tables 2+3
Results of individual studies	20	For all outcomes considered (benefits or harms), present, for each study: (a) simple summary data for each intervention group (b) effect estimates and confidence intervals, ideally with a forest plot.	Tables 2+3, +p18-20
Synthesis of results	21	Present results of each meta-analysis done, including confidence intervals and measures of consistency.	No meta-analysis, discussed p18-20
Risk of bias across studies	22	Present results of any assessment of risk of bias across studies (see Item 15).	discussed p21
Additional analysis	23	Give results of additional analyses, if done (e.g., sensitivity or subgroup analyses, meta-regression [see Item 16]).	18-20
**DISCUSSION**			
Summary of evidence	24	Summarize the main findings including the strength of evidence for each main outcome; consider their relevance to key groups (e.g., healthcare providers, users, and policy makers).	20-21
Limitations	25	Discuss limitations at study and outcome level (e.g., risk of bias), and at review-level (e.g., incomplete retrieval of identified research, reporting bias).	20-21
Conclusions	26	Provide a general interpretation of the results in the context of other evidence, and implications for future research.	21-22
**FUNDING**			
Funding	27	Describe sources of funding for the systematic review and other support (e.g., supply of data); role of funders for the systematic review.	22

From: Moher D, Liberati A, Tetzlaff J, Altman DG, The PRISMA Group (2009). Preferred Reporting Items for Systematic Reviews and Meta-Analyses: The PRISMA Statement. PLoS Med 6(7): e1000097. doi:10.1371/journal.pmed1000097

For more information, visit: **www.prisma-statement.org**.

For peer review only - http://bmjopen.bmj.com/site/about/guidelines.xhtml
